# Supplementary material for: Histamine signaling and metabolism identify potential biomarkers and therapies for lymphangioleiomyomatosis
Source: EMBO Mol Med. 2021 Aug 11;13(9):e13929. doi: 10.15252/emmm.202113929 (PMC8422079; doi:10.15252/emmm.202113929)
Supplement: Supplementary file 2 — Appendix [file EMMM-13-e13929-s007.pdf]

Histamine signaling and metabolism identify potential biomarkers and therapies for lymphangioleiomyomatosis

Appendix

Table of contents

Appendix Figure S1: Additional monoamine metabolites studied in LAM plasma.

Appendix Figure S2: Controls of immunohistochemical assays.

Appendix Figure S3: Gene expression analyses of LAM lung nodules using the GSE12027 dataset.

Appendix Figure S4: *HRH1* expression association with breast cancer metastasis to lung.

Appendix Figure S5: Overexpression of *Slc22a2* and *Slc22a3* in *Tsc2*-deficient MEFs.

Appendix Figure S6: MAO expression and activity in 105K cells.

Appendix Figure S7: Detection of mast cells in *Tsc2*-deficient 105K tumors.

Appendix Figure S8: Quantification of rapamycin blood and tumor levels with co-administration of loratadine.

Appendix Figure S9: shRNA-mediated depletion of *Maoa* and *Hrh1* in *Tsc2*-deficient 105K cells.

Appendix Figure S10: Evaluation of the effect of shRNA-mediated depletion of *Maoa* or *Hrh1* expression, and/or rapamycin administration.

Appendix Figure S11: The *in vitro* responses of rapamycin and loratadine are positively correlated in the NCI-60 cancer cell panel.

Appendix Figure S12: Evaluation of AKT and S6 phosphorylation sites and levels in *Tsc2*-deficient cell models exposed *in vitro* to different drugs.

Appendix Figure S13: Evaluation of cell death in *Tsc2*-deficient 105K tumors.

Appendix Figure S14: Evaluation of autophagy induction in *Tsc2*-deficient 105K cells.

Appendix Figure S15: Evaluation of cell proliferation in *Tsc2*-deficient 105K tumors.

Appendix Figure S16: Overrepresented GO terms in genes differentially expressed between *Tsc2*-deficient 105K tumors treated with rapamycin and/or loratadine.

Appendix Figure S17: Representative images of hematoxylin-eosin-stained *Tsc2*-deficient 105K tumors treated with shRNA control or targeting *Maoa* or *Hrh1*.

Appendix Figure S18: Representative images of hematoxylin-eosin-stained *Tsc2*-deficient 105K tumors from mice administered a L-histidine analog or low L-histidine diet, and their corresponding controls.

Appendix Figure S19: Quantification of epithelial and mesenchymal gene marker expression in *Tsc2*-deficient 105K cells transduced *in vitro* with shRNAs against *Maoa* or *Hrh1* expression.

Appendix Figure S20: Inhibition of invasion and migration of *Tsc2*-deficient 105K cells transduced *in vitro* with shRNAs against *Maoa* or *Hrh1* expression.

Appendix Table S1A. Genes coding for enzymes and with expression negatively correlated with *TSC2* in lung-metastatic breast cancer.

Appendix Table S1B. Quantified metabolites in plasma samples from healthy women, LAM and related pulmonary disease patients.

Appendix Table S2. Clinical characteristics of LAM patients at time of sampling and used for LC/MS-MS biomarker validation assays (Spanish cohort).

Appendix Table S3. Clinical characteristics of rapamycin-off LAM patients at time of sampling (UK cohort).

Appendix Table S4. Clinical characteristics of women with LAM in the Polish cohort who were analyzed for the effect of rapamycin combined with loratadine.

Appendix Table S5. Histopathological evaluation of 105K *Tsc2*-deficient tumors treated with vehicle, drugs in monotherapy, or rapamycin combinations.

Appendix Table S6. Primers used in RT-PCR assays.

Appendix Table S7. Antibodies used in study.

Appendix Figure S1

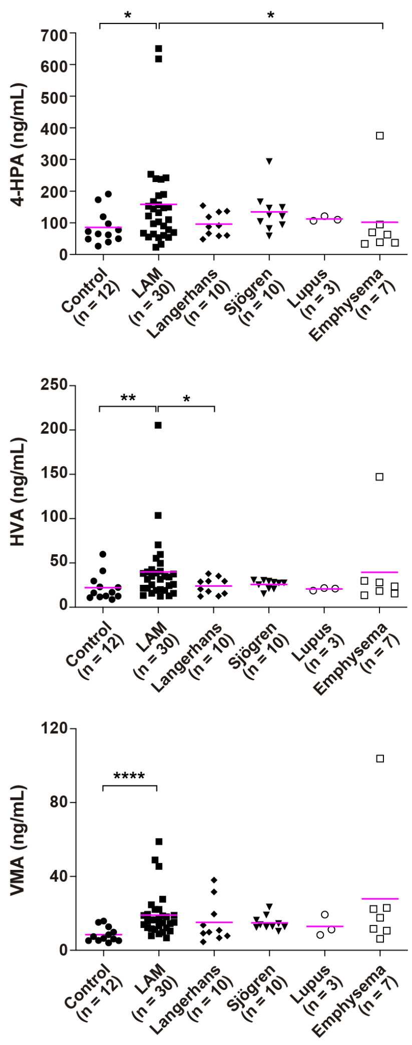

Appendix Figure S1. Additional monoamine metabolites studied in LAM plasma. Detailed results of 4-HPA, HVA, and VMA in healthy controls, LAM and related pulmonary disease patients. The asterisks indicate significant differences based on two-sided Mann-Whitney tests (4-HPA, control-LAM P = 0.024, LAM-Langerhans P = 0.09, LAM-Sjögren P = 0.48, LAM-Lupus P = 0.40, and LAM-emphysema P = 0.042; HVA, control-LAM P = 0.008, LAM-Langerhans P = 0.037, LAM-Sjögren P = 0.10, LAM-Lupus P = 0.09, and LAM-emphysema P = 0.14; and VMA, control-LAM $P = 3\times10^{-5}$, LAM-Langerhans P = 0.06, LAM-Sjögren P = 0.21, LAM-Lupus P = 0.18, and LAM-emphysema P = 0.44). Average values are indicated with lilac-colored lines.

Appendix Figure S2

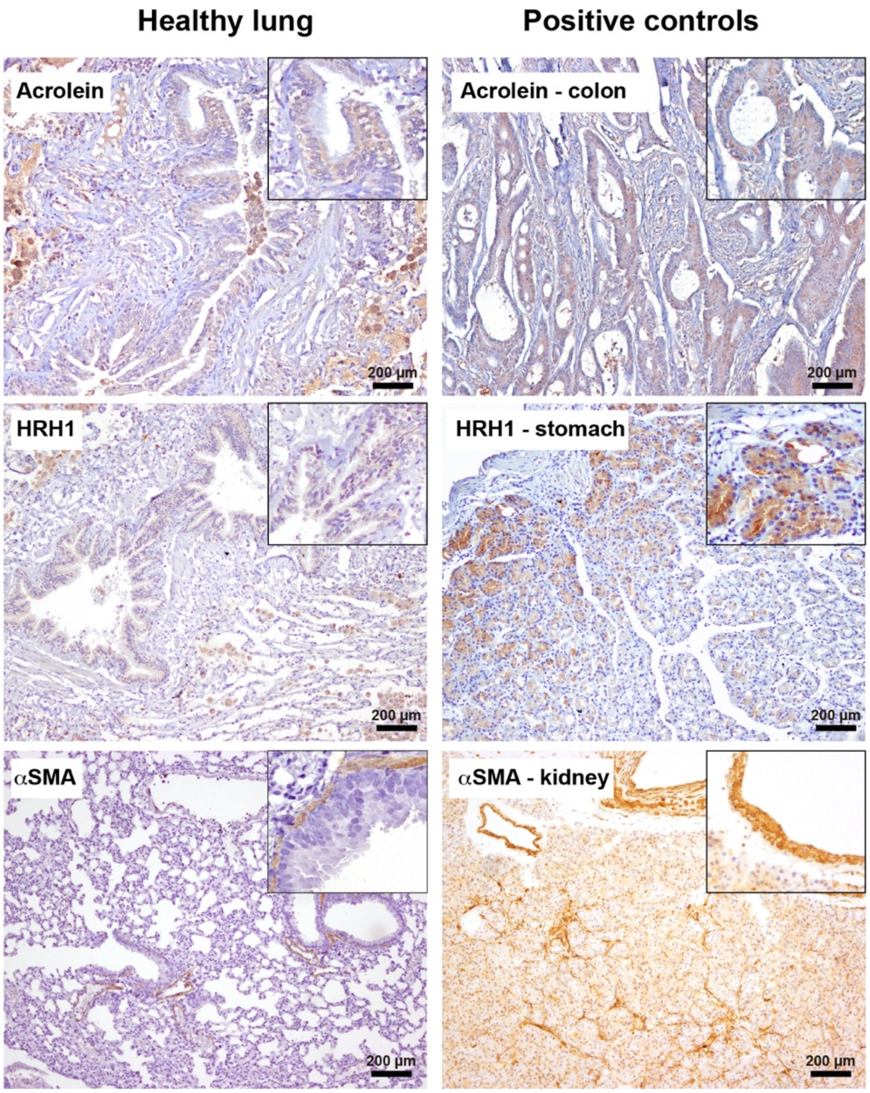

Appendix Figure S2. Controls of immunohistochemical assays. Left panels, results in healthy lung; right panels, assay-positive tissue. Acrolein and HRH1 showed positivity in the luminal layer of the bronchioles, as portrayed in the insets (left panels). Scale bars are shown.

Appendix Figure S3

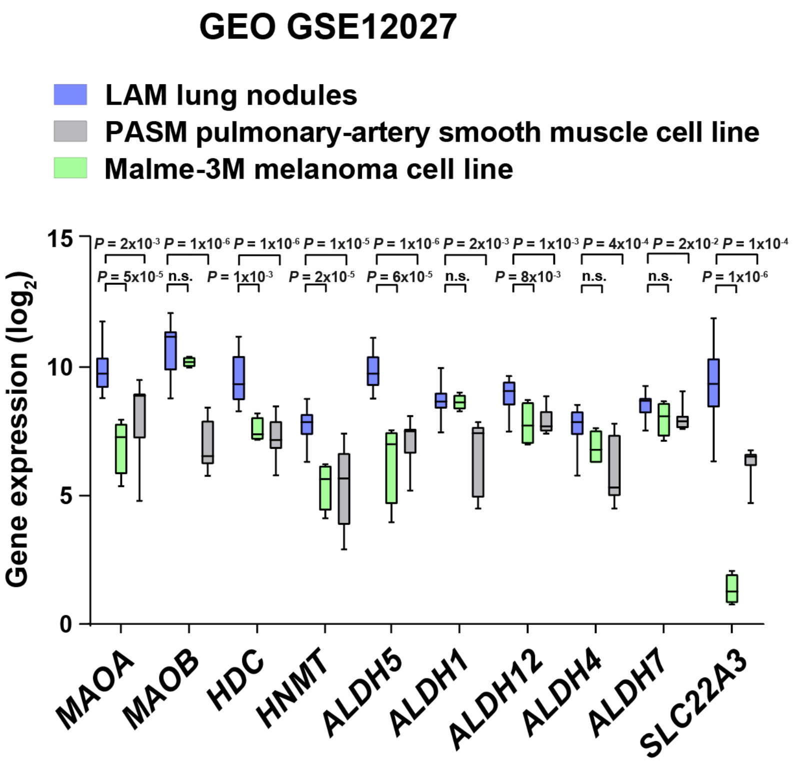

MAOA	***Monoamine oxidase A***
MAOB	***Monoamine oxidase B***
HDC	***Histidine decarboxylase***
HNMT	***Histamine N-methyltransferase***
ALDH	***Aldehyde dehydrogenase***
SLC22A3	***Solute carrier family 22 member 3 (organic cation transporter (OCT) 3)***

Appendix Figure S3. Gene expression analyses of LAM lung nodules using the GSE12027 dataset. The differences are indicated (two-sided t-test *P* values; n.s., not significant) between the LAM lung nodules and PASM (pulmonary-artery smooth muscle) or Malme-3M (melanoma) cell profiles. Boxplot with whiskers from minimum to maximum.

Appendix Figure S4

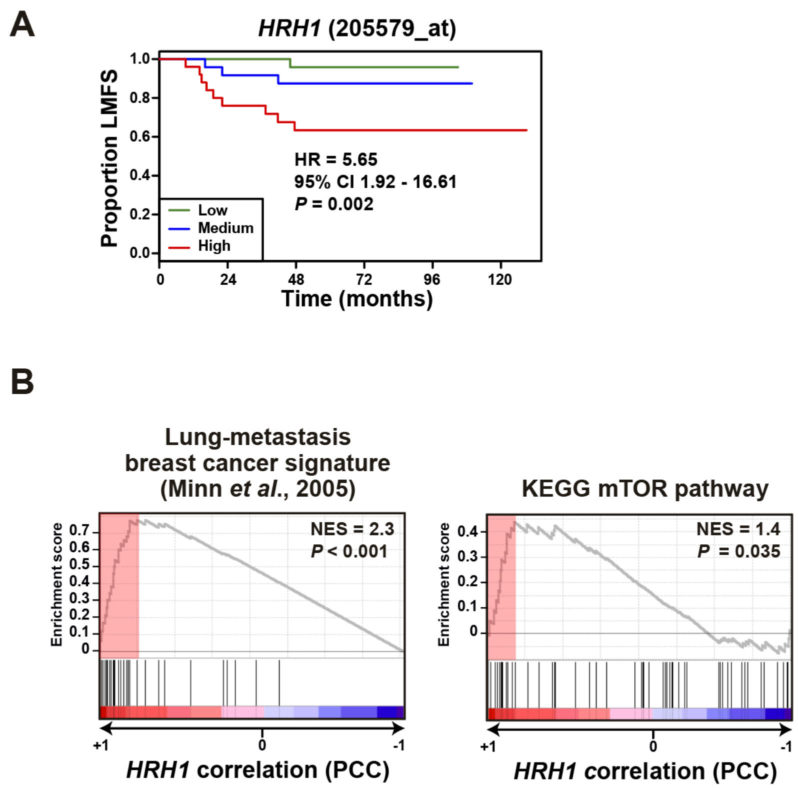

Appendix Figure S4. *HRH1* expression association with breast cancer metastasis to lung. A Kaplan-Meier lung metastasis-free survival (LMFS) curves based on categorization of *HRH1* expression in breast tumors. Multivariate Cox proportional-hazards regression results are shown: HR, hazard ratio; CI, confidence interval; and *P* value. **B** Gene Set Enrichment Analysis (GSEA) results showing positive expression correlations (based on PCCs) between *HRH1* and key gene sets as indicated. The GSEA normalized enrichment score (NES) and associated *P* values are indicated (1,000 permutations).

Appendix Figure S5

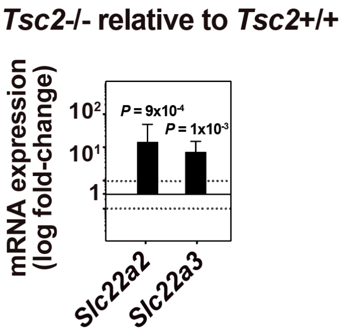

Appendix Figure S5. Overexpression of *Slc22a2* and *Slc22a3* in *Tsc2*-deficient MEFs. Gene expression level is indicated by a log_{10}-fold change relative to *Tsc2* wild-type MEFs. The significant differences correspond to two-sided t-test (*P* values are indicated; replicates/condition n = 5, independent experiments n = 2). The bars indicate mean ± SD. Dotted horizontal lines indicate 2-fold (top) and 0.5-fold (bottom).

Appendix Figure S6

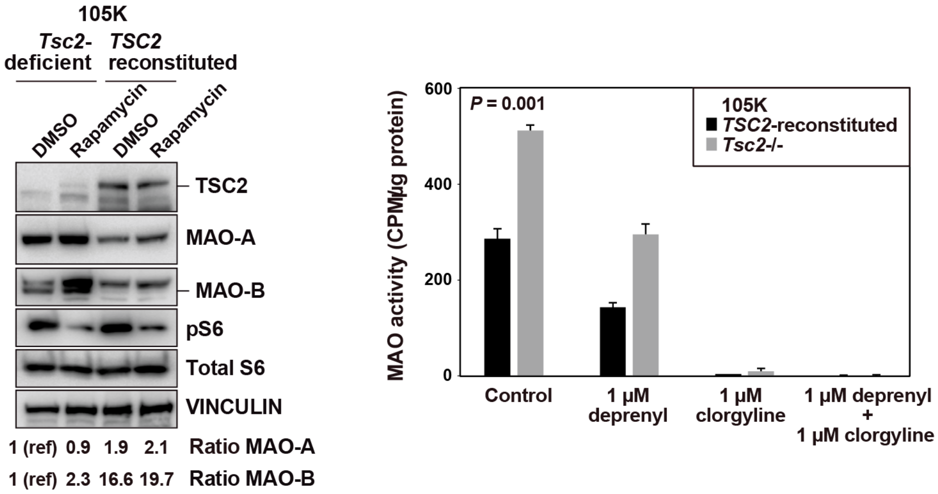

Appendix Figure S6. MAO expression and activity in 105K cells. Left panel, MAO-A/B overexpression in *Tsc2*-deficient relative to *TSC2*-reconstituted 105K cells, both grown in DMEM 10% FBS. The expression levels are indicated by the ratio relative to each loading control and basal setting (noted as 1(ref)). Right panel, higher MAO basal activity (Y-axis) in *Tsc2*-deficient relative to *TSC2*-reconstituted 105K cells, both grown in DMEM 10% FBS. The inhibitors (depicted on the X-axis) were added to cell extracts prior to activity assay to assess the contribution of each MAO isoform. The significant difference corresponds to two-way ANOVA test (replicates/condition n = 4 and independent experiments n = 2). CPM: counts per minute. The bars indicate mean ± SEM.

Appendix Figure S7

***Tsc2*-deficient 105k tumors**
Mast cells (toluidine blue)

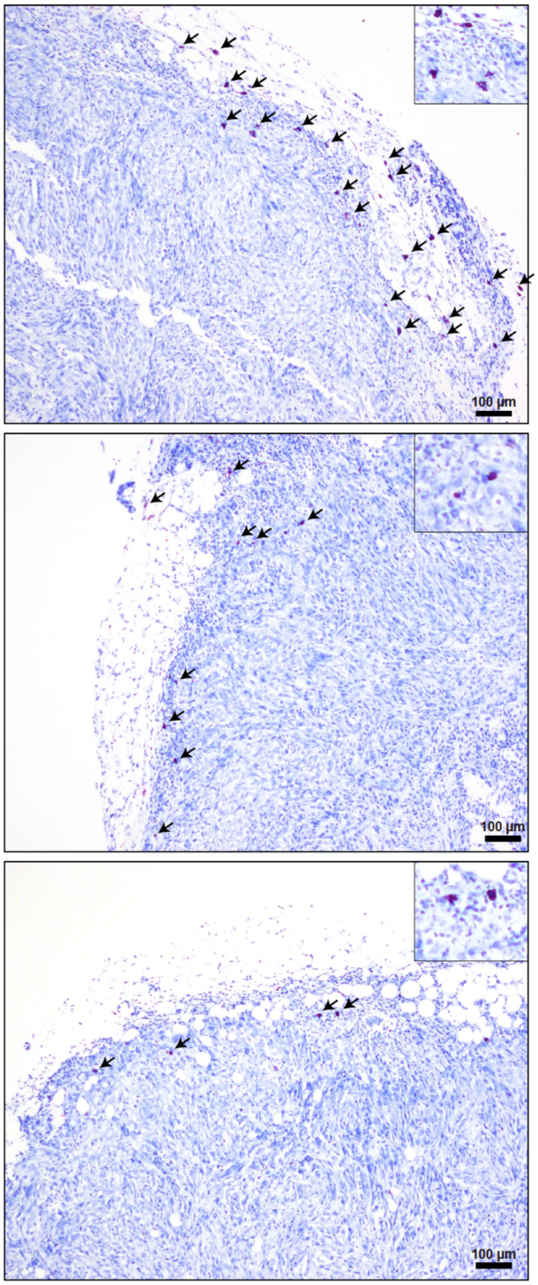

Appendix Figure S7. Detection of mast cells in *Tsc2*-deficient 105K tumors. Representative images of toluidine blue stain denoting presence of mast cells in the front (arrows) of three tumors. The insets include magnified images. Scale bars are shown.

Appendix Figure S8

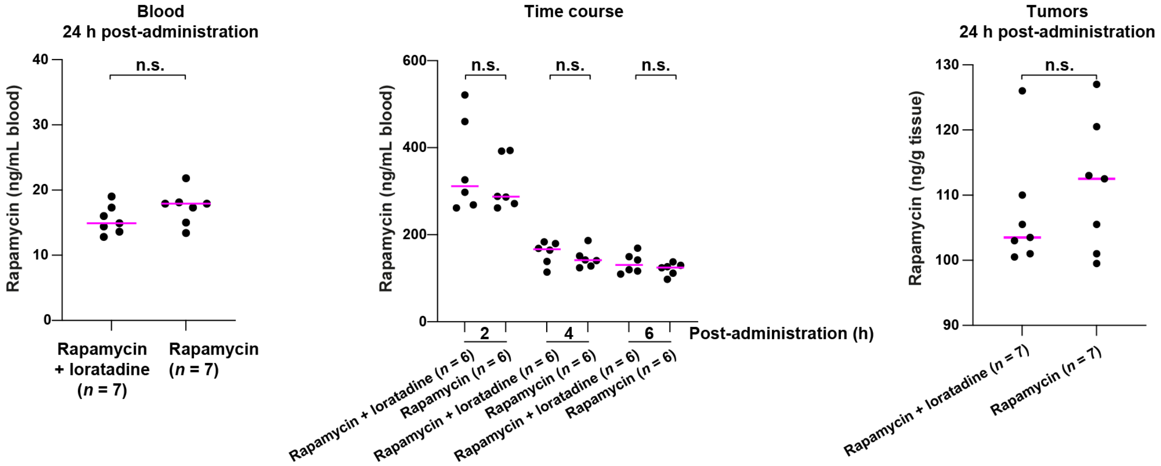

Appendix Figure S8. Quantification of rapamycin blood and tumor levels with co-administration of loratadine. *Tsc2*-deficient 105K cells were engrafted in C57BL/6J mice and, when tumors reached a volume of 100-150 mm^3, the animals were treated as described with rapamycin alone or combined with loratadine. No differences were observed in blood concentration of rapamycin either after 24 hours of drug administration (left panel) or at any of the times after administration of rapamycin and loratadine (2, 4, and 6 hours; middle panel). In addition, no differences were observed in treated tumors after 24 hours of drug administration (right panel). Significance was assessed with two-sided Mann-Whitney tests (n.s., not significant). The number (n) of samples in each group is indicated. Average values are indicated with lilac-colored lines.

Appendix Figure S9

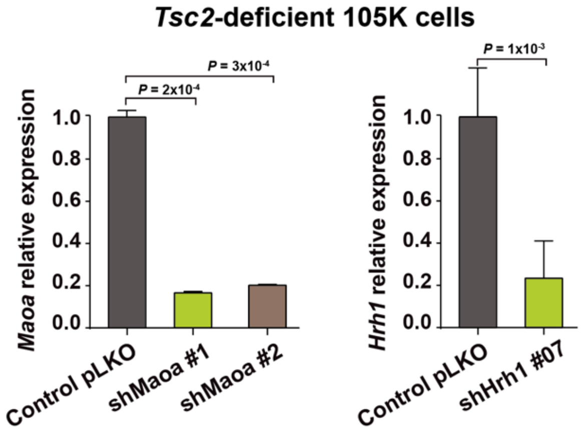

Appendix Figure S9. shRNA-mediated depletion of *Maoa* and *Hrh1* in *Tsc2*-deficient 105K cells. Graphs showing *Maoa* (left) and *Hrh1* (right) expression depletion relative to pLKO-transduced *Tsc2*-deficient 105K cells prior to injection in mice. The expression reduction relative to pLKO was determined with one-sided t-test (*P* values are indicated; replicates/condition n = 3, independent experiments n = 3). The bars indicate mean ± SD.

Appendix Figure S10

A

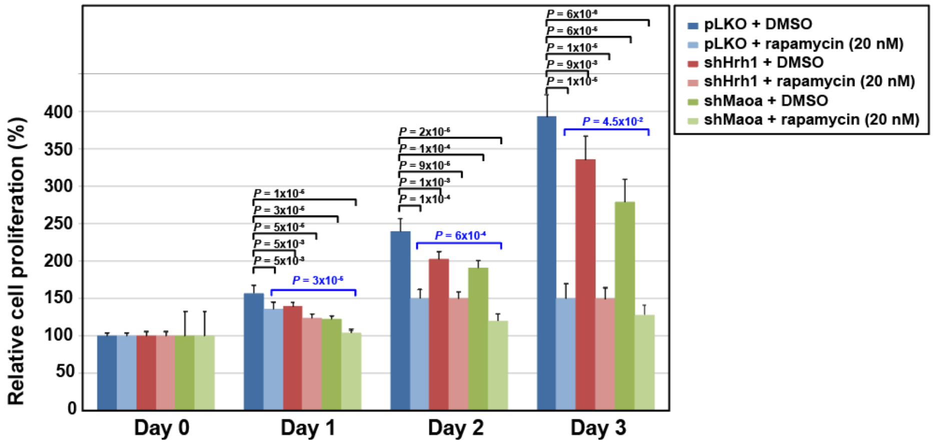

B

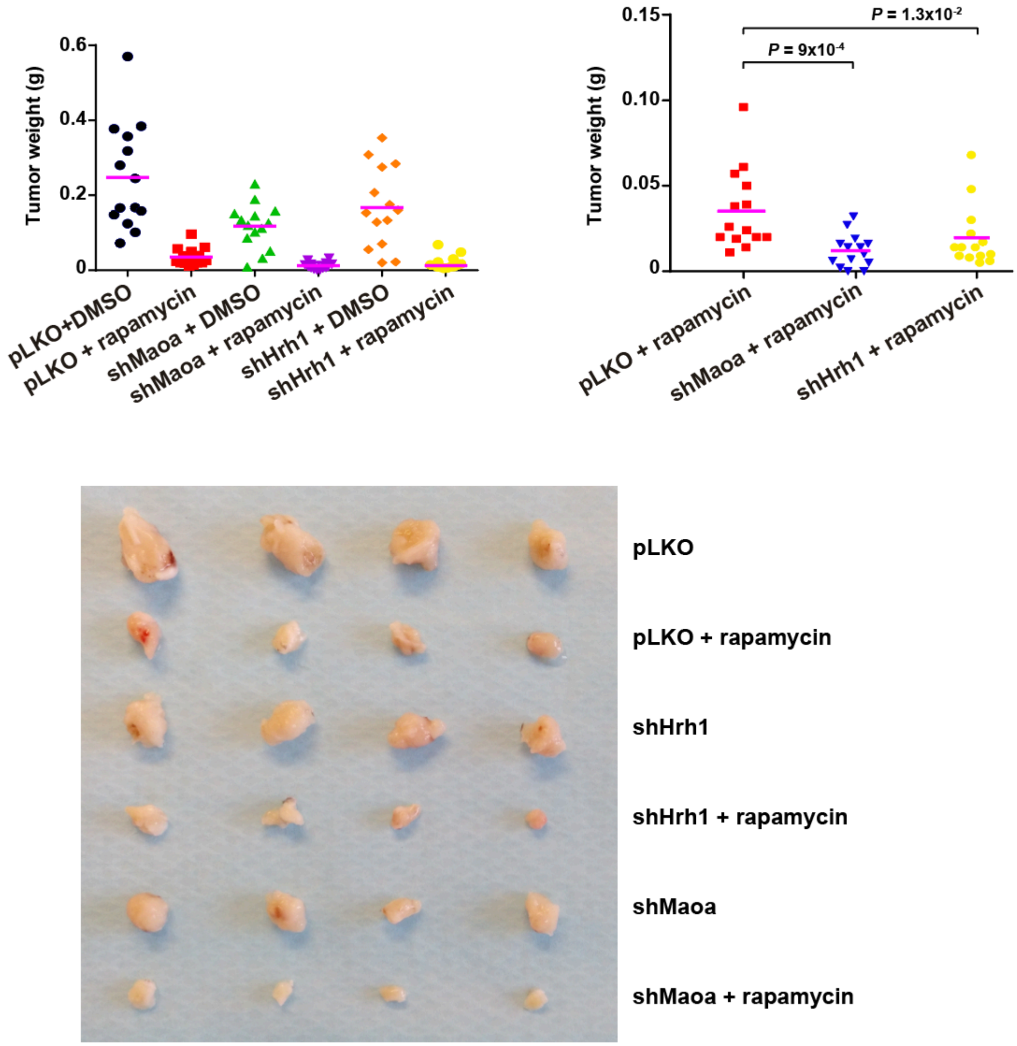

Appendix Figure S10. Evaluation of the effect of shRNA-mediated depletion of *Maoa* or *Hrh1* expression, and/or rapamycin administration. A Inhibition of *Tsc2*-deficient 105K cell viability with rapamycin and with single transduction of shRNA against *Maoa* or *Hrh1* expression, relative to pLKO control (DMSO conditions). The combination of shRNA-*Maoa* and rapamycin exposure further decreases viability relative to pLKO and rapamycin (comparison marked with blue bar/asterisk). The differences relative to pLKO-DMSO were determined with two-tailed t-test (*P* values are indicated; replicates/condition n = 6, and independent experiments n = 2). The bars indicate mean ± SEM. **B** Top panels, *Tsc2*-deficient 105K tumor weight (g) differences at the end of the depicted *in vivo* assays (X-axis; mice/group, n). Top right panel, significant reduction in the shRNA-*Maoa*/Hrh1 and rapamycin-treated groups relative to rapamycin alone; two-sided Mann-Whitney test (*P* values are indicated). Average weights are indicated with lilac-colored lines. Bottom panel, representative images of four tumors from each group.

Appendix Figure S11

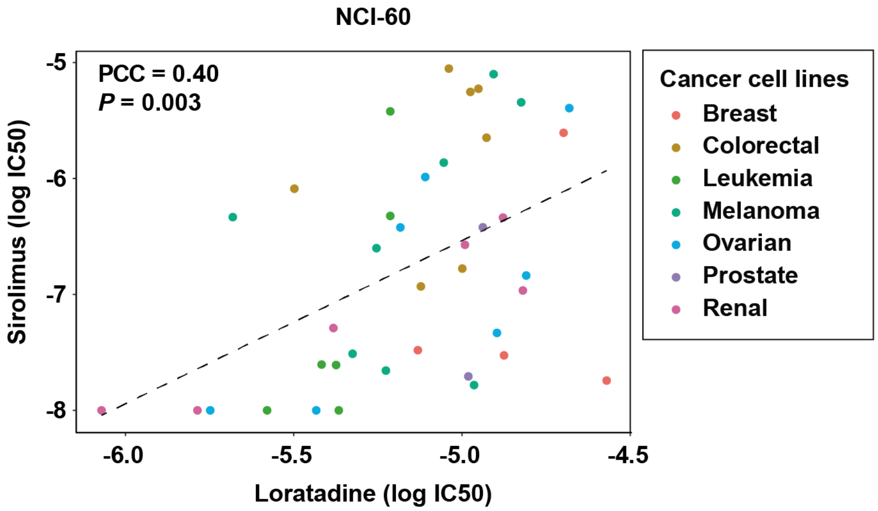

Appendix Figure S11. The *in vitro* responses of rapamycin and loratadine are positively correlated in the NCI-60 cancer cell panel. Scatter-plot of drug responses (log of half-maximal inhibitory concentration (IC50) across cancer cell lines (inset depicts cancer types). The PCC estimate and *P* value are indicated.

Appendix Figure S12

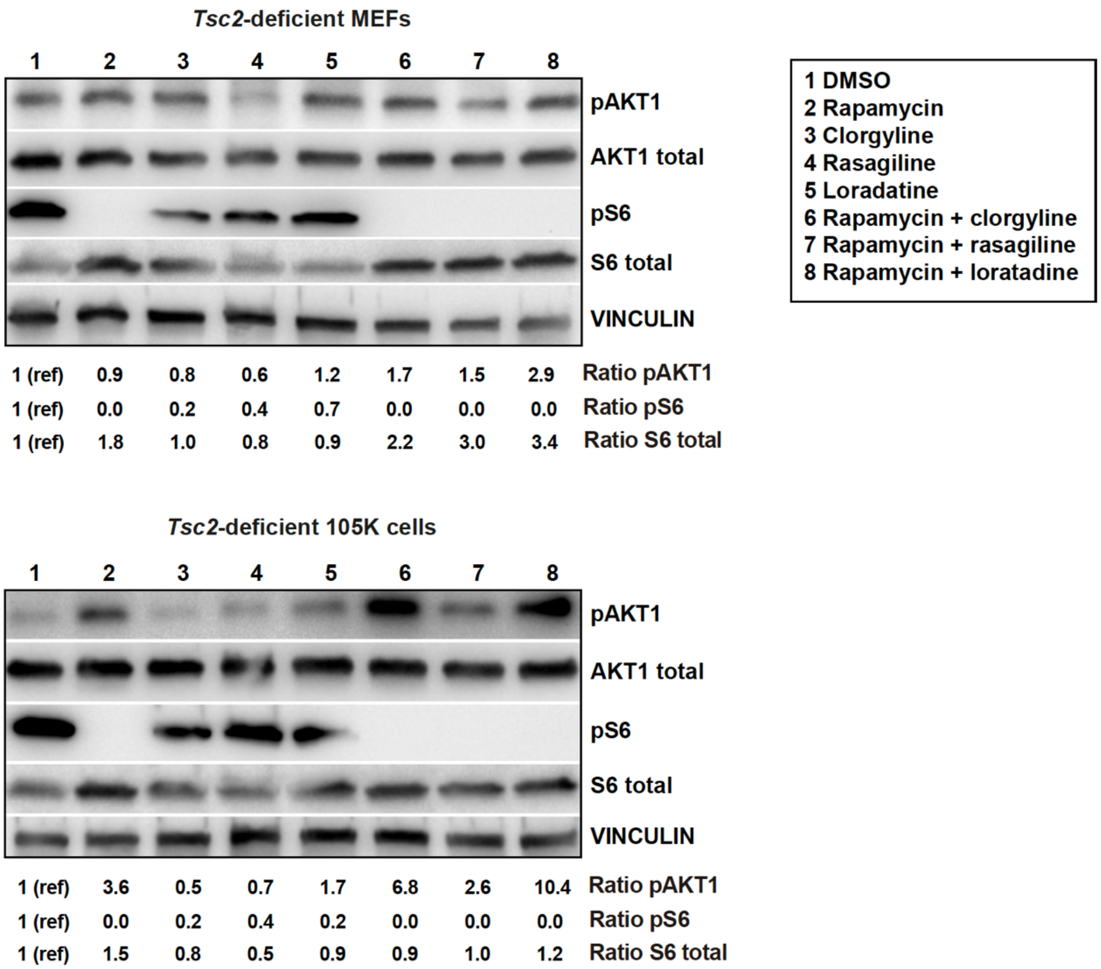

Appendix Figure S12. Evaluation of AKT and S6 phosphorylation sites and levels in *Tsc2*-deficient cell models exposed *in vitro* to different drugs. Western blot results of phospho-Ser473 and total AKT, phospho-Ser235/236 and total S6, and loading control (vinculin) in *Tsc2*-deficient MEF (top panels) and *Tsc2*-deficient 105K cells (bottom panels). The treatment conditions are depicted in the inset and the concentrations used were the same as in previous *in vitro* assays. The inferred expression level of each marker for each condition is indicated by the ratio between the corresponding phospho-signal and total expression (pAKT/total AKT, and pS6/total S6), or total S6 versus loading control (noted as 1 (reference (ref)).

Appendix Figure S13

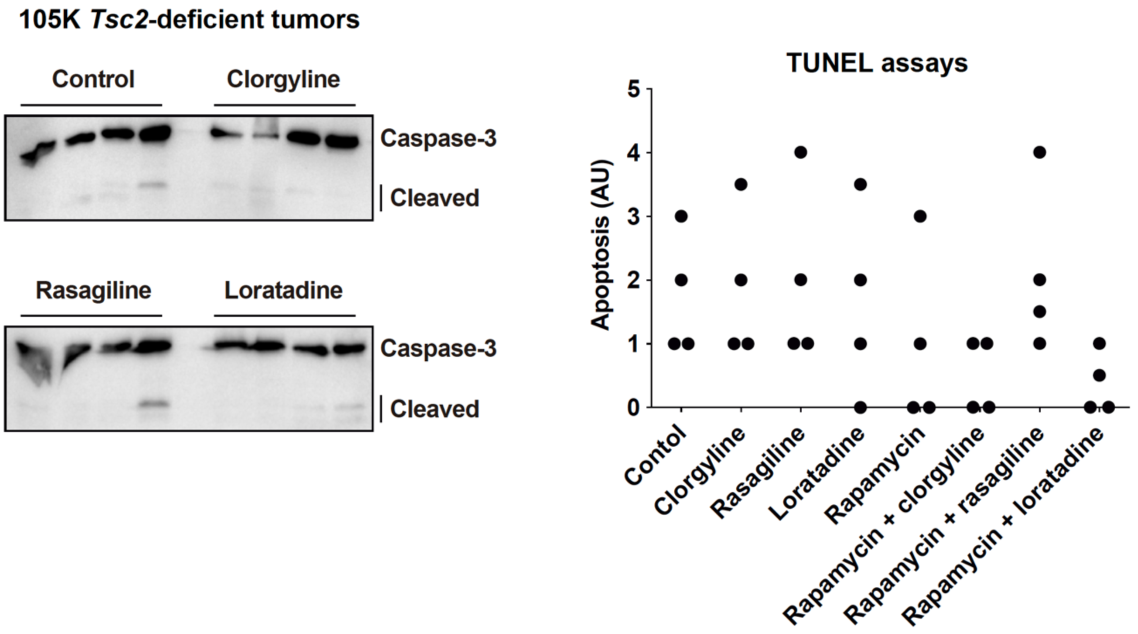

Appendix Figure S13. Evaluation of cell death in *Tsc2*-deficient 105K treated tumors. Left panels, western blot results of caspase-3 in four tumors of each treatment group, as indicated. Right panel, arbitrary quantification (score 0-5) of TUNEL-mediated detection of apoptosis in paraffin-embedded tumor tissue from controls, monotherapies, and rapamycin-combined therapies (X-axis; four tumors analyzed in each setting). No significant differences were observed.

Appendix Figure S14

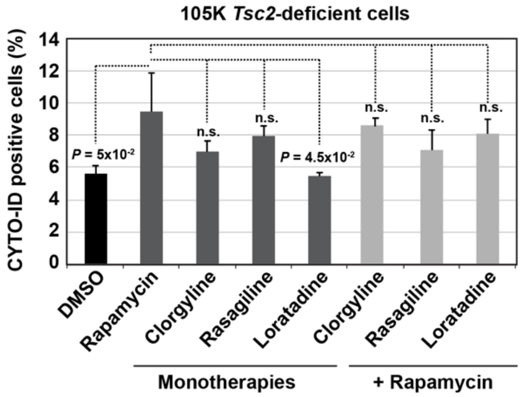

Appendix Figure S14. Evaluation of autophagy induction in *Tsc2*-deficient 105K cells. Graphs showing the percentages of CYTO-ID-positive *Tsc2*-deficient 105K cells treated for 24 hours with DMSO or drugs *in vitro* with 10% FBS complete medium. Clorgyline 1 μM, loratadine 100 nM, rasagiline 1 μM, and rapamycin 20 nM. The differences relative to rapamycin alone were determined with two-sided t-test (*P* values are indicated; n.s., not significant; replicates/condition n = 3). The bars indicate mean ± SD.

Appendix Figure S15

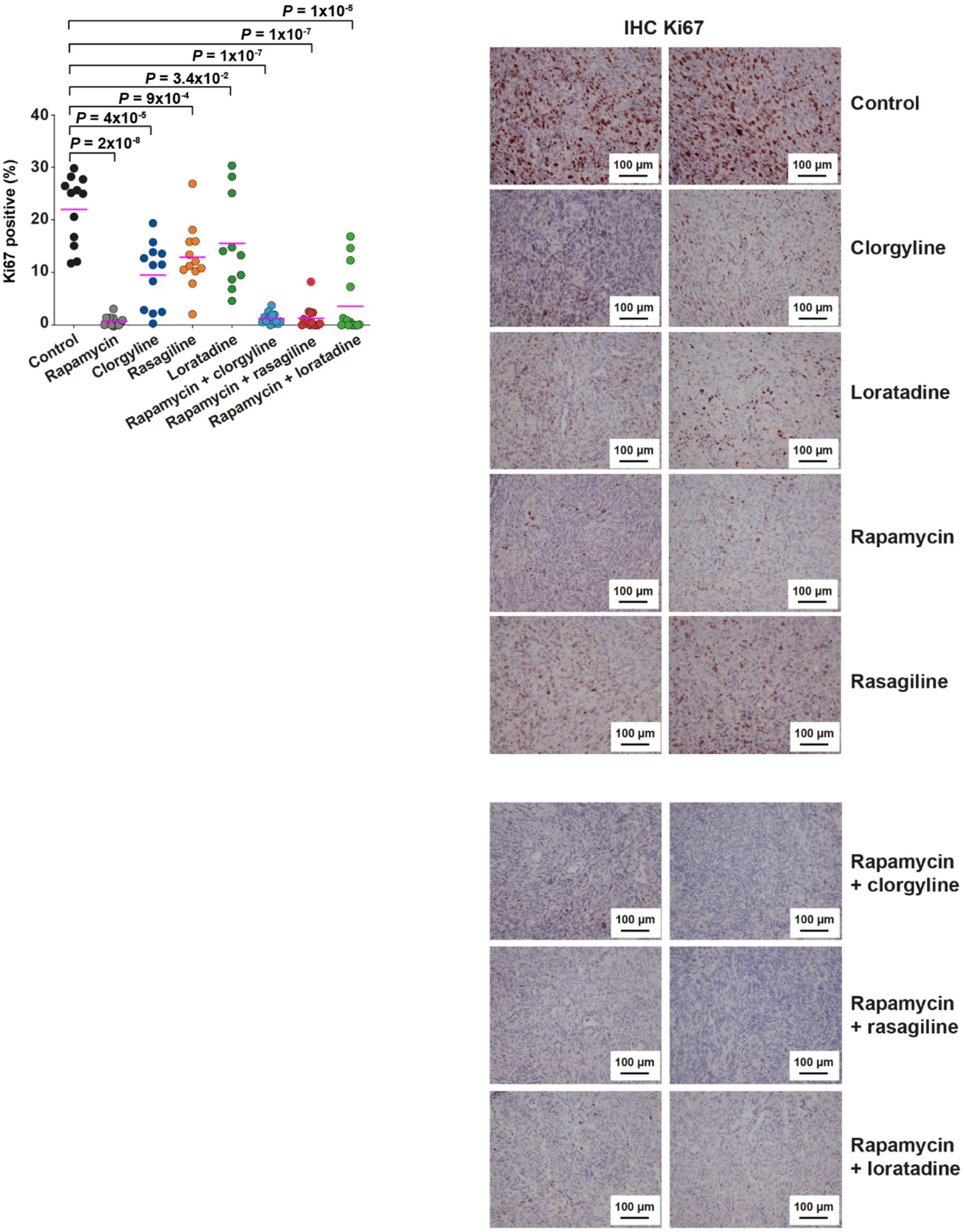

Appendix Figure S15. Evaluation of cell proliferation in *Tsc2*-deficient 105K tumors. Left panel, quantification of Ki67 immunostaining in tumors across experimental conditions (X-axis). Significant Ki67 decrease relative to the control group was determined with one-tailed t-test (*P* values are indicated). Right panels, representative images of Ki67 staining in two tumors of each treatment group. Scale bars are shown.

Appendix Figure S16

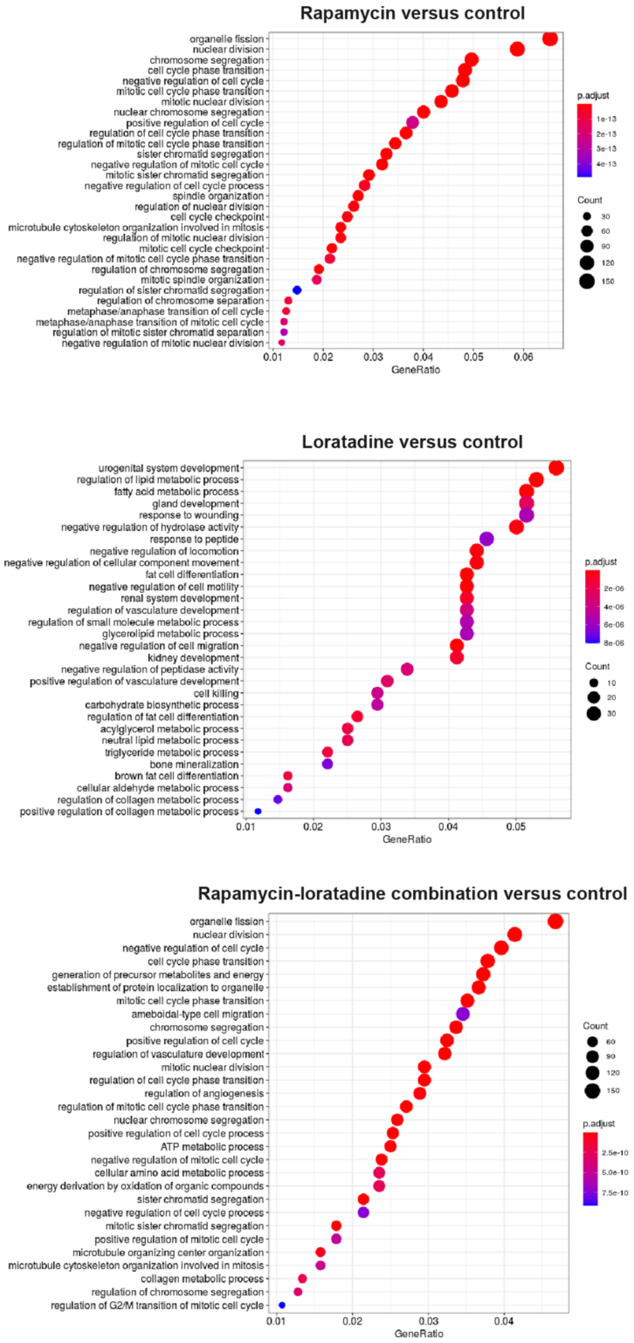

Appendix Figure S16. Overrepresented GO terms in genes differentially expressed between *Tsc2*-deficient 105K tumors treated with rapamycin and/or loratadine. Each panel shows the significant GO terms (false discovery rate adjustment) linked to differential gene expression under a given condition (rapamycin and/or loratadine) relative to control (CMC)-treated tumors.

Appendix Figure S17

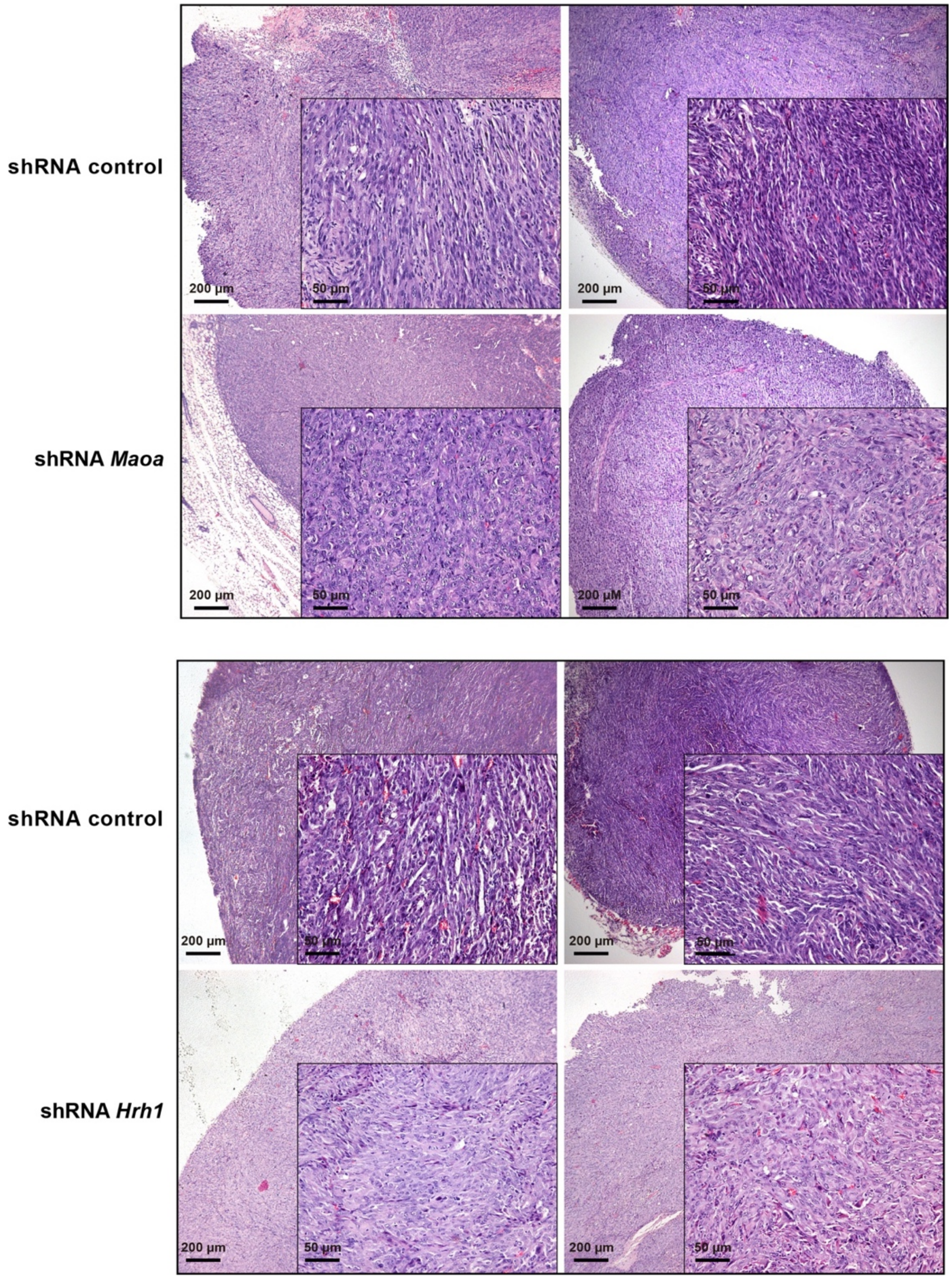

Appendix Figure S17. Representative images of hematoxylin-eosin-stained *Tsc2*-deficient 105K tumors treated with shRNA control or targeting *Maoa* or *Hrh1*. Two tumor samples are shown for each experimental condition. Targeting *Maoa* or *Hrh1* enhanced the epithelioid morphology. Scale bars are shown.

Appendix Figure S18

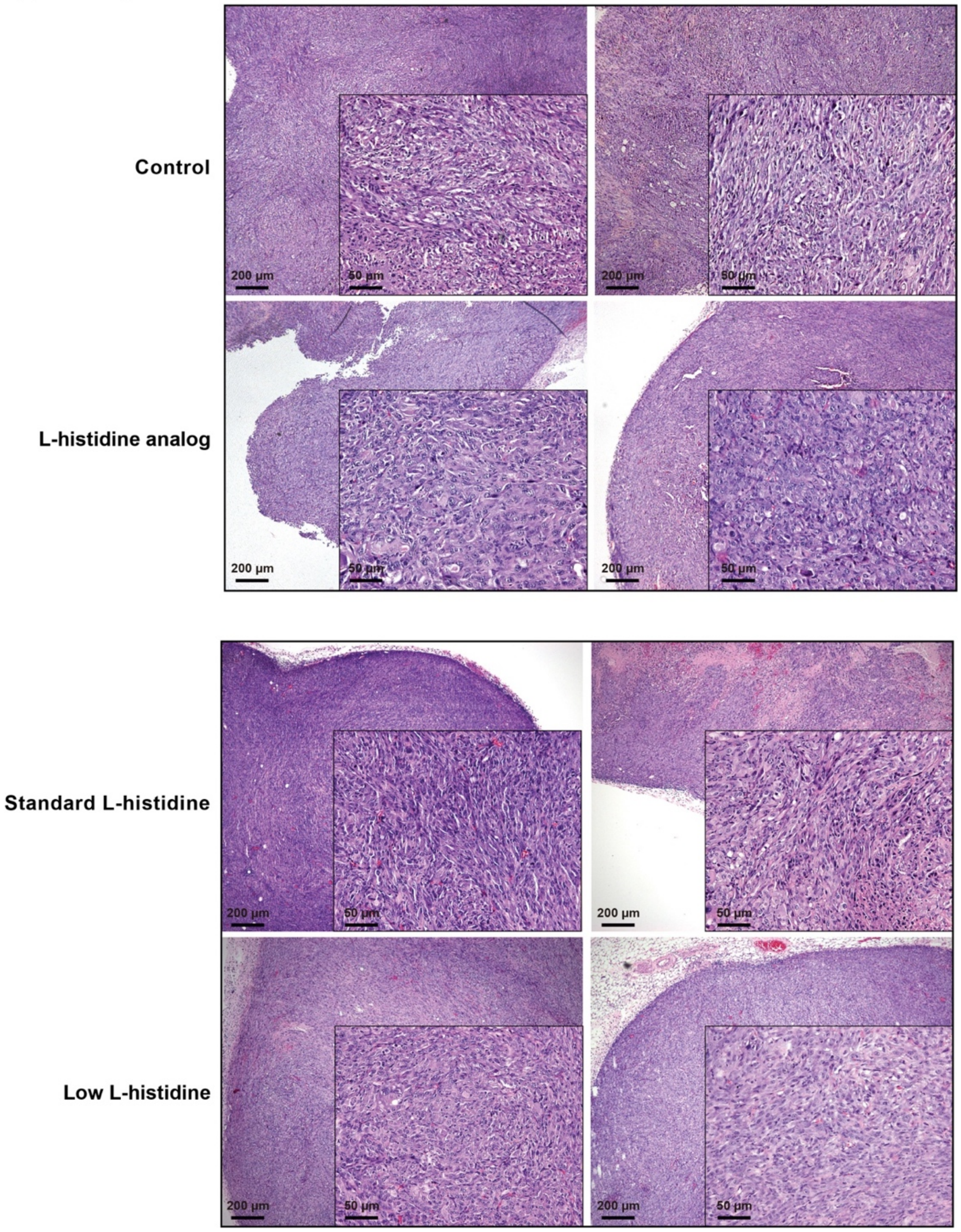

Appendix Figure S18. Representative images of hematoxylin-eosin-stained *Tsc2*-deficient 105K tumors from mice administered a L-histidine analog or low L-histidine diet, and their corresponding controls. Two tumor samples are shown for each experimental condition. Administration of an L-histidine analog or a low L-histidine diet enhanced the epithelioid morphology. Scale bars are shown.

Appendix Figure S19

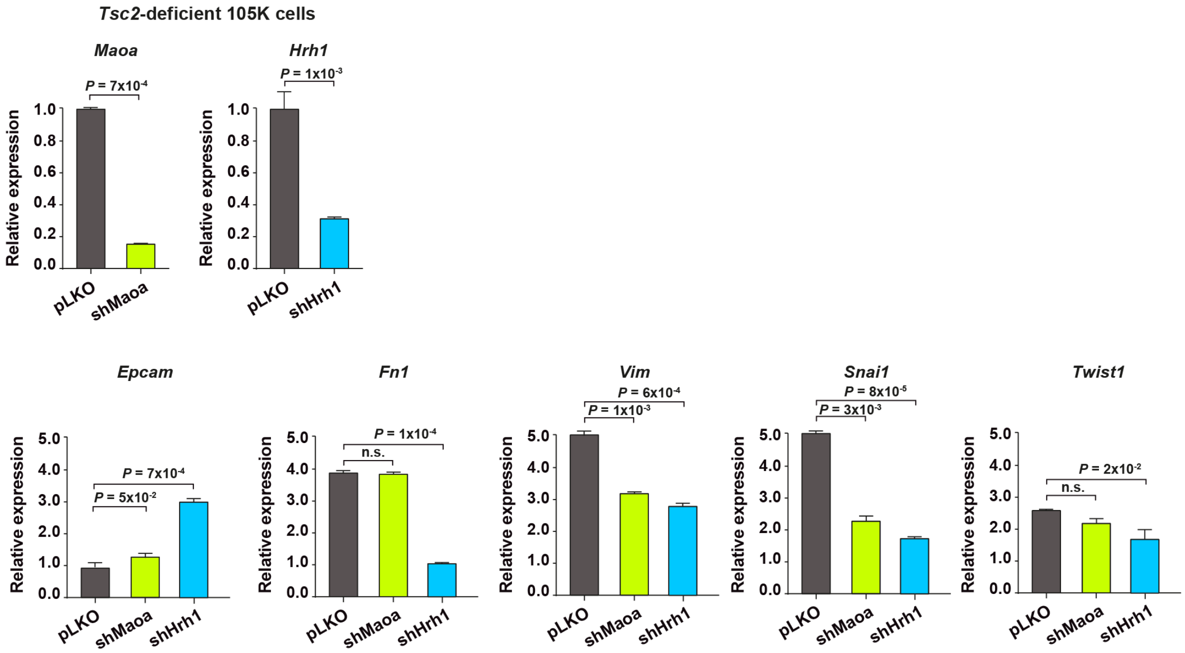

Appendix Figure S19. Quantification of epithelial and mesenchymal gene marker expression in *Tsc2*-deficient 105K cells transduced *in vitro* with shRNAs against *Maoa* or *Hrh1* expression, relative to pLKO control. Top panels, confirmation of *Maoa* and *Hrh1* expression depletion in the corresponding cell assays (two-sided t-test *P* values are indicated; replicates/condition n = 3, independent experiments n = 3). Bottom panels, over-expression of *Epcam*, and under-expression of *Fn1*, *Snai1*, *Vim*, and *Twist1* in cells with reduced expression of *Maoa* or *Hrh1* expression. The differences relative to pLKO were determined with two-sided t-test (*P* values are indicated; n.s., not significant; replicates/condition n = 3, independent experiments n = 3). The bars indicate mean ± SD.

Appendix Figure S20

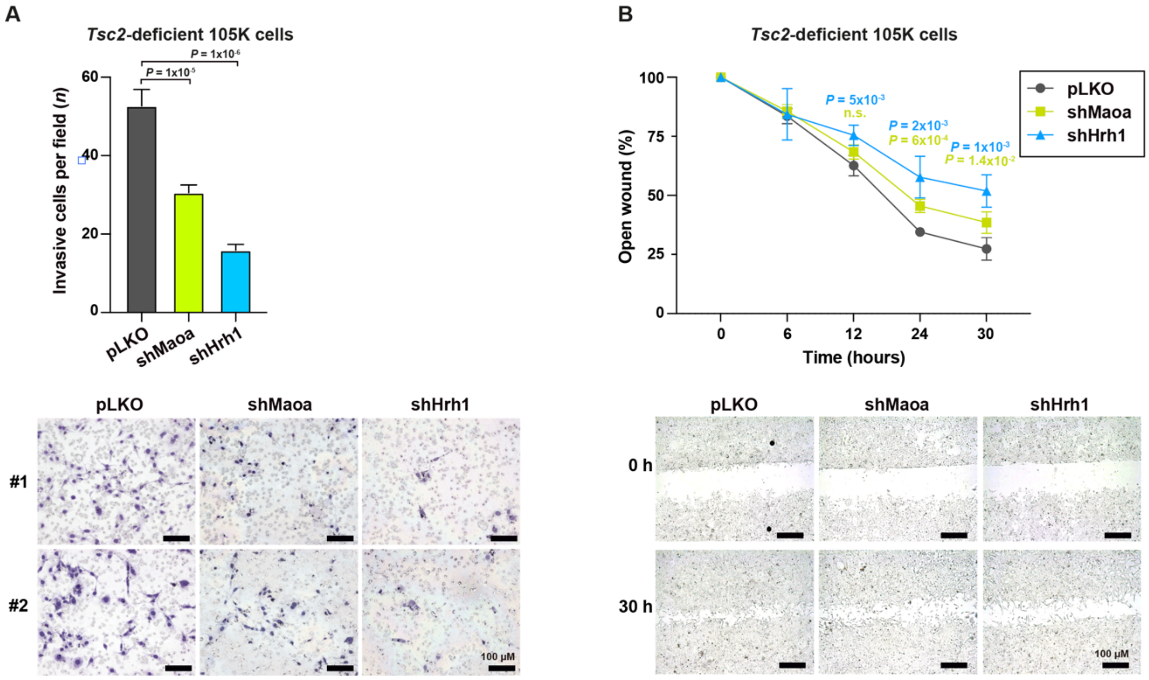

Appendix Figure S20. Inhibition of invasion and migration of *Tsc2*-deficient 105K cells transduced *in vitro* with shRNAs against *Maoa* or *Hrh1* expression, relative to pLKO control. A Graph depicting the frequency of invasive cells per field with different shRNA transductions (X-axis). The differences relative to pLKO were determined with two-sided t-test (*P* values are indicated; field/condition n = 16, independent experiments n = 2). The bars indicate mean ± SEM. **B** Graph depicting wound closure (from 0 to 30 hours) with different shRNA transductions (inset). The differences relative to pLKO were determined with two-sided t-test (*P* values are indicated; n.s., not significant; replicates/condition n = 3, independent experiments n = 2). Bottom panels, representative wound images at 0 and 30 hours. Each data point represents the mean and SD.

Appendix Table S1A. Genes coding for enzymes and with expression significantly negatively correlated with *TSC2* in lung-metastatic breast cancer.

Gene name	Protein name	Entry	EC number	Entrez
ADAMTS5 *ADAMTS11 ADMP2*	A disintegrin and metalloproteinase with thrombospondin motifs 5 (ADAM-TS 5) (ADAM-TS5) (ADAMTS-5) (EC 3.4.24.-) (A disintegrin and metalloproteinase with thrombospondin motifs 11) (ADAM-TS 11) (ADAMTS-11) (ADMP-2) (Aggrecanase-2)	Q9UNA0	3.4.24.-	11096
ADCY3 *KIAA0511*	Adenylate cyclase type 3 (EC 4.6.1.1) (ATP pyrophosphate-lyase 3) (Adenylate cyclase type III) (AC-III) (Adenylate cyclase, olfactive type) (Adenylyl cyclase 3) (AC3)	O60266	4.6.1.1	109
AKR1C1 *DDH DDH1*	Aldo-keto reductase family 1 member C1 (EC 1.1.1.-) (20-alpha-hydroxysteroid dehydrogenase) (20-alpha-HSD) (EC 1.1.1.149) (Chlordecone reductase homolog HAKRC) (Dihydrodiol dehydrogenase 1/2) (DD1/DD2) (High-affinity hepatic bile acid-binding protein) (HBAB) (Indanol dehydrogenase) (EC 1.1.1.112) (Tra	Q04828	1.1.1.-; 1.1.1.149; 1.1.1.112; 1.3.1.20	1645
AKR1C2 *DDH2*	Aldo-keto reductase family 1 member C2 (EC 1.-.-.-) (3-alpha-HSD3) (Chlordecone reductase homolog HAKRD) (Dihydrodiol dehydrogenase 2) (DD-2) (DD2) (Dihydrodiol dehydrogenase/bile acid-binding protein) (DD/BABP) (Trans-1,2-dihydrobenzene-1,2-diol dehydrogenase) (EC 1.3.1.20) (Type III 3-alpha-hydrox	P52895	1.-.-.-; 1.3.1.20; 1.1.1.357	1646
AKR1C3 *DDH1 HSD17B5 KIAA0119 PGFS*	Aldo-keto reductase family 1 member C3 (EC 1.-.-.-) (17-beta-hydroxysteroid dehydrogenase type 5) (17-beta-HSD 5) (3-alpha-HSD type II, brain) (3-alpha-hydroxysteroid dehydrogenase type 2) (3-alpha-HSD type 2) (EC 1.1.1.357) (Chlordecone reductase homolog HAKRb) (Dihydrodiol dehydrogenase 3) (DD-3) (DD	P42330	1.-.-.-; 1.1.1.357; 1.1.1.112; 1.1.1.188; 1.1.1.239; 1.1.1.64; 1.3.1.20	8644
ALDH1A3 *ALDH6*	Aldehyde dehydrogenase family 1 member A3 (EC 1.2.1.5) (Aldehyde dehydrogenase 6) (Retinaldehyde dehydrogenase 3) (RALDH-3) (RalDH3)	P47895	1.2.1.5	220
ATP10B *ATPVB KIAA0715*	Probable phospholipid-transporting ATPase VB (EC 3.6.3.1) (ATPase class V type 10B) (P4-ATPase flippase complex alpha subunit ATP10B)	O94823	3.6.3.1	23120
C1R	Complement C1r subcomponent (EC 3.4.21.41) (Complement component 1 subcomponent r) [Cleaved into: Complement C1r subcomponent heavy chain; Complement C1r subcomponent light chain]	P00736	3.4.21.41	715
CA3	Carbonic anhydrase 3 (EC 4.2.1.1) (Carbonate dehydratase III) (Carbonic anhydrase III) (CA-III)	P07451	4.2.1.1	761
MCM2 BM28 ***CCNL1*** *CDCL1 KIAA0030*	DNA replication licensing factor MCM2 (EC 3.6.4.12) (Minichromosome maintenance protein 2 homolog) (Nuclear protein BM28)	P49736	3.6.4.12	4171
CYLD *CYLD1 KIAA0849 HSPC057*	Ubiquitin carboxyl-terminal hydrolase CYLD (EC 3.4.19.12) (Deubiquitinating enzyme CYLD) (Ubiquitin thioesterase CYLD) (Ubiquitin-specific-processing protease CYLD)	Q9NQC7	3.4.19.12	1540
DDX3X *DBX DDX3*	ATP-dependent RNA helicase DDX3X (EC 3.6.4.13) (DEAD box protein 3, X-chromosomal) (DEAD box, X isoform) (Helicase-like protein 2) (HLP2)	O00571	3.6.4.13	1654
DIMT1 ***DIMT1L*** *HUSSY-05*	Probable dimethyladenosine transferase (EC 2.1.1.183) (DIM1 dimethyladenosine transferase 1 homolog) (DIM1 dimethyladenosine transferase 1-like) (Probable 18S rRNA (adenine(1779)-N(6)/adenine(1780)-N(6))-dimethyltransferase) (Probable 18S rRNA dimethylase) (Probable S-adenosylmethionine-6-N',N'-adenosy	Q9UNQ2	2.1.1.183	27292
COLGALT2 C1orf17 ***GLT25D2*** *KIAA0584*	Procollagen galactosyltransferase 2 (EC 2.4.1.50) (Collagen beta(1-O)galactosyltransferase 2) (Glycosyltransferase 25 family member 2) (Hydroxylysine galactosyltransferase 2)	Q8IYK4	2.4.1.50	23127
P3H2 ***LEPREL1*** *MLAT4*	Prolyl 3-hydroxylase 2 (EC 1.14.11.7) (Leprecan-like protein 1) (Myxoid liposarcoma-associated protein 4)	Q8IVL5	1.14.11.7	55214
MET	Hepatocyte growth factor receptor (HGF receptor) (EC 2.7.10.1) (HGF/SF receptor) (Proto-oncogene c-Met) (Scatter factor receptor) (SF receptor) (Tyrosine-protein kinase Met)	P08581	2.7.10.1	4233
NMT2	Glycylpeptide N-tetradecanoyltransferase 2 (EC 2.3.1.97) (Myristoyl-CoA:protein N-myristoyltransferase 2) (NMT 2) (Peptide N-myristoyltransferase 2) (Type II N-myristoyltransferase)	O60551	2.3.1.97	9397
NTRK3 *TRKC*	NT-3 growth factor receptor (EC 2.7.10.1) (GP145-TrkC) (Trk-C) (Neurotrophic tyrosine kinase receptor type 3) (TrkC tyrosine kinase)	Q16288	2.7.10.1	4916
PAPOLA *PAP*	Poly(A) polymerase alpha (PAP-alpha) (EC 2.7.7.19) (Polynucleotide adenylyltransferase alpha)	P51003	2.7.7.19	10914
PDGFRA *PDGFR2 RHEPDGFRA*	Platelet-derived growth factor receptor alpha (PDGF-R-alpha) (PDGFR-alpha) (EC 2.7.10.1) (Alpha platelet-derived growth factor receptor) (Alpha-type platelet-derived growth factor receptor) (CD140 antigen-like family member A) (CD140a antigen) (Platelet-derived growth factor alpha receptor) (Platelet-derived growth f	P16234	2.7.10.1	5156
CPQ LCH1 ***PGCP***	Carboxypeptidase Q (EC 3.4.17.-) (Lysosomal dipeptidase) (Plasma glutamate carboxypeptidase)	Q9Y646	3.4.17.-	10404
PRKD2 ***PKD2*** *HSPC187*	Serine/threonine-protein kinase D2 (EC 2.7.11.13) (nPKC-D2)	Q9BZL6	2.7.11.13	25865
PLA2G4A *CPLA2 PLA2G4*	Cytosolic phospholipase A2 (cPLA2) (Phospholipase A2 group IVA) [Includes: Phospholipase A2 (EC 3.1.1.4) (Phosphatidylcholine 2-acylhydrolase); Lysophospholipase (EC 3.1.1.5)]	P47712	3.1.1.4; 3.1.1.5	5321
PLD1	Phospholipase D1 (PLD 1) (hPLD1) (EC 3.1.4.4) (Choline phosphatase 1) (Phosphatidylcholine-hydrolyzing phospholipase D1)	Q13393	3.1.4.4	5337
PTGIS *CYP8 CYP8A1*	Prostacyclin synthase (EC 5.3.99.4) (Prostaglandin I2 synthase)	Q16647	5.3.99.4	5740
PTGS2 *COX2*	Prostaglandin G/H synthase 2 (EC 1.14.99.1) (Cyclooxygenase-2) (COX-2) (PHS II) (Prostaglandin H2 synthase 2) (PGH synthase 2) (PGHS-2) (Prostaglandin-endoperoxide synthase 2)	P35354	1.14.99.1	5743
RANBP2 *NUP358*	E3 SUMO-protein ligase RanBP2 (EC 6.3.2.-) (358 kDa nucleoporin) (Nuclear pore complex protein Nup358) (Nucleoporin Nup358) (Ran-binding protein 2) (RanBP2) (p270)	P49792	6.3.2.-	5903
TGFBR2	TGF-beta receptor type-2 (TGFR-2) (EC 2.7.11.30) (TGF-beta type II receptor) (Transforming growth factor-beta receptor type II) (TGF-beta receptor type II) (TbetaR-II)	P37173	2.7.11.30	7048
TPST1	Protein-tyrosine sulfotransferase 1 (EC 2.8.2.20) (Tyrosylprotein sulfotransferase 1) (TPST-1)	O60507	2.8.2.20	8460
UBE2I *UBC9 UBCE9*	SUMO-conjugating enzyme UBC9 (EC 6.3.2.-) (SUMO-protein ligase) (Ubiquitin carrier protein 9) (Ubiquitin carrier protein I) (Ubiquitin-conjugating enzyme E2 I) (Ubiquitin-protein ligase I) (p18)	P63279	6.3.2.-	7329

Appendix Table S1B. Quantified metabolites in plasma samples from healthy women, LAM and related pulmonary disease patients.

Metabolite	Acronym
3,4-Dihydroxymandelic acid	DOMA
3,4-Dihydroxyphenylacetic acid	DOPAC
Homovanillic acid	HVA
4-Hydroxyphenylacetic acid	4-HPA
3-Methoxy-4-hydroxymandelic acid	VMA
3-Methoxy-4-hydroxymandelic acid D3 (I.S.)	
Methylimidazoleacetic acid (1-Methyimidazole-4-acetic acid)	MIAA
Phenylacetic acid	PAA

Appendix Table S2. Clinical characteristics of LAM patients at time of sampling and used for LC/MS-MS biomarker validation assays (Spanish cohort).

ID	Age at sample extraction (years)	TSC	Treatment	Pneumothorax (n)	AML
14	49	No	Oxygen, rapamycin	0	No
19	46	Yes	Oxygen, progesterone, rapamycin	3	Yes
23	44	No	Oxygen, rapamycin	1	No
47	51	No	Progesterone (medroxyprogesterone acetate), formoterol	4	No
53	52	Yes	None	1	No
67	45	No	Rapamycin, bronchodilators, simvastatin, decapeptyl	0	No
79	49	No	None	7	Yes
82	56	No	Rapamycin	0	Yes
112	46	No	Rapamycin	1	No
116	42	No	Rapamycin	2	Yes
117	59	No	Oxygen, progesterone	4	No
122	65	No	Rapamycin	1	Yes
55	52	Yes	Ovariectomy, oxygen	0	Yes
1	56	No	None	0	Yes
2	51	No	Symbicort	0	No
7	55	No	Progesterone, tamoxifen	0	No
9	54	No	Progesterone, rapamycin, bronchodilators	3	No
22	56	No	Progesterone	0	No
24	51	No	Oxygen, rapamycin	0	No
44	48	No	Progesterone, rapamycin	0	Yes
51	53	No	Rapamycin	0	No
52	49	No	Bronchodilators	0	Yes
62	41	No	Rapamycin	3	Yes
70	47	Yes	Ovariectomy, rapamycin, oxygen	5	Yes
101	46	No	None	0	No
102	25	No	None	1	No
105	39	No	Progesterone, rapamycin	13	No
109	36	No	None	1	No
114	56	No	Oxygen, progesterone	1	Yes
115	47	No	Rapamycin	0	No

Appendix Table S3. Clinical characteristics of rapamycin-off LAM patients at time of sampling (UK cohort).

ID	Age at sample extraction (years)	TSC diagnosis	Pneumothorax (n)	AML diagnosis	FEV_1 (% predicted)	D_{LCO} (% predicted)	Disease burden
1	41	No	1	Yes	66	72	2
2	34	No	0	Yes	117	75	1
3	32	Yes	4	No	114	68	0
4	53	Yes	1	Yes	99	69	1
5	62	No	1	Yes	77	59	1
6	64	No	1	Yes	65	72	2
7	53	No	1	Yes	93	60	1
8	49	No	1	Yes	83	76	1
9	41	No	1	Yes	94	67	1
10	53	No	0	Yes	85	77	1
11	51	No	0	No	81	59	0
12	45	No	1	Yes	52	65	2
13	65	No	0	Yes	59	54	2
14	45	No	0	Yes	63	38	2
15	45	No	0	No	60	62	1
16	36	Yes	3	Yes	74	57	1
17	44	No	0	Yes	80	51	1
18	51	No	1	Yes	81	64	1
19	51	No	0	Yes	85	51	1
20	73	No	0	No	26	34	1

Appendix Table S4. Clinical characteristics of women with LAM in the Polish cohort who were analyzed for the effect of rapamycin combined with loratadine.

ID	Patient group	Age time of diagnosis	Age in 2019	Smoking (packs/year)	TSC diagnosis	Perivascular epithelioid cell tumor	AML	Chylothorax	Pneumothorax	Lymphangioma	Asthma
1	Sirolimus with no allergy	52	46	0	No	No	0	0	0	1	0
2	Sirolimus with no allergy	30	34	6	Yes	No	1	0	0	0	0
3	Sirolimus with no allergy	49	56	15	No	No	0	0	1	0	0
4	Sirolimus with no allergy	47	50	20	No	Yes	0	0	0	1	0
5	Sirolimus with no allergy	26	26	16	No	No	1	0	1	0	0
6	Sirolimus with no allergy	35	37	0	Yes	No	1	0	0	1	0
7	Sirolimus with no allergy	37	47	0	No	Yes	1	1	1	1	0
8	Sirolimus with no allergy	42	44	20	No	No	1	0	1	1	0
9	Sirolimus with no allergy	39	55	0	No	No	0	1	0	1	1
10	Sirolimus with no allergy	48	49	10	No	No	0	1	0	0	0
11	Sirolimus with no allergy	50	66	0	Yes	No	1	0	0	0	0
12	Sirolimus with no allergy	36	47	10	Yes	No	1	0	1	0	0
13	Sirolimus with no allergy	45	45	0	Yes	No	1	0	0	0	0
14	Sirolimus with no allergy	36	45	4	Yes	No	1	0	1	0	0
15	Sirolimus with no allergy	43	76	0	No	No	1	1	0	0	0
16	Sirolimus with no allergy	35	36	0	No	No	0	1	0	1	1
17	Sirolimus with no allergy	28	35	0	No	Yes	1	1	0	1	0
18	Sirolimus with no allergy	43	46	0	No	No	0	0	1	0	0
19	Sirolimus with no allergy	48	52	0	No	No	1	0	0	0	0
20	Sirolimus with no allergy	39	40	0	No	No	1	1	0	1	0
21	Sirolimus with no allergy	32	36	0	No	Yes	0	1	1	1	0
22	Sirolimus with no allergy	31	40	0	Yes	No	1	0	0	0	0
23	Sirolimus with no allergy	30	37	0	No	No	1	0	0	0	0
24	Sirolimus with no allergy	47	47	0	No	No	0	0	0	0	1
25	Sirolimus with no allergy	31	32	0	No	No	1	0	0	0	0
26	Sirolimus with no allergy	35	37	12	Yes	No	1	0	0	0	0
27	Sirolimus with no allergy	51	69	0	No	No	1	0	0	0	0
28	Sirolimus with no allergy	28	33	0	No	Yes	0	1	1	1	0
29	Sirolimus with no allergy	40	40	0	No	No	0	1	0	1	0
30	Sirolimus with no allergy	32	35	0	Yes	No	1	0	0	0	0
31	Sirolimus with no allergy	54	56	0	No	No	1	0	0	0	0
32	Sirolimus with no allergy	57	57	30	No	No	1	0	0	0	0
33	Sirolimus with no allergy	19	26	0	Yes	No	0	0	0	0	0
34	Sirolimus with no allergy	26	30	4	No	No	1	0	1	0	0
35	Sirolimus with no allergy	24	31	0	Yes	No	0	0	0	0	0
36	Sirolimus with no allergy	29	32	0	Yes	No	1	0	0	0	0
37	Sirolimus with no allergy	40	43	8	No	Yes	0	0	1	1	1
38	Sirolimus with no allergy	33	39	0	Yes	No	1	1	0	1	0
39	Sirolimus with no allergy	35	45	10	No	No	0	0	1	0	0
40	Sirolimus with no allergy	37	47	0	Yes	No	1	0	0	0	0
41	Sirolimus with no allergy	30	34	0	Yes	No	1	0	1	0	0
42	Sirolimus with no allergy	33	38	0	Yes	No	1	0	0	0	0
43	Sirolimus with no allergy	32	33	0	No	No	1	0	1	1	0
44	Sirolimus + loratadine	46	62	0	No	No	0	0	0	0	1
45	Sirolimus + loratadine	48	64	2	No	No	0	0	1	0	1
46	Sirolimus + loratadine	39	44	7.5	No	No	1	0	0	0	1
47	Sirolimus + loratadine	39	44	0	No	No	0	0	1	0	1
48	Sirolimus + loratadine	47	57	0	No	No	0	0	0	0	1
49	Sirolimus + loratadine	35	42	0	No	Yes	0	1	0	1	1
50	Sirolimus + loratadine	49	58	22	No	No	1	0	0	0	1
51	Sirolimus + loratadine	30	38	2	No	Yes	1	0	1	1	1
52	Sirolimus + loratadine	47	47	5	No	No	0	0	0	0	1
53	Sirolimus + loratadine	56	63	0	No	No	1	0	0	0	1
54	Sirolimus + loratadine	43	51	0	No	No	1	0	0	0	1
55	Sirolimus + loratadine	40	44	6	No	Yes	0	0	0	1	1
56	Sirolimus + loratadine	40	49	0	No	No	1	0	0	0	1
57	Sirolimus + loratadine	29	37	0	No	No	0	0	1	1	1
58	Sirolimus + loratadine	31	33	3	No	No	0	0	0	1	1
59	Sirolimus + loratadine	64	76	7	No	No	1	0	0	0	1
60	Sirolimus + loratadine	45	53	1.5	No	Yes	0	0	1	1	1
61	Sirolimus + loratadine	47	52	1	No	Yes	0	0	0	1	1
62	Sirolimus + loratadine	24	26	0	No	No	1	0	1	0	1
63	Sirolimus + loratadine	46	57	2	No	No	0	1	0	0	1
64	Sirolimus + loratadine	44	48	0	No	Yes	0	0	1	1	1
65	Sirolimus + loratadine	41	58	0	No	Yes	0	1	0	1	1

Appendix Table S5. Histopathological evaluation of 105K *Tsc2*-deficient tumors treated with drugs in monotherapy or rapamycin-combined. H&E stained slides from the tumors were evaluated by a pathologist blinded to the treatment groups. The extent of the spindle or epithelioid cell morphology, glandular differentiation, presence of pleomorphic tumor cells, necrosis, and fibrosis were classified as: 1+, < 5% of the tumor; 2+, between 5% and 50% of the tumor; and 3+, > 50% of the tumor. Cytological atypia was graded as mild (1+), moderate (2+) and severe (3+).

Treatment group	Tumor ID	Spindle	Epithelioid	Glandular	Atypia	Pleomorphic	Necrosis	Fibrosis
Vehicle	B1R	3	0	2	3	1	2	0
Vehicle	E1R	3	1	1	3	0	1	0
Vehicle	H5R	2	3	0	3	2	2	0
Vehicle	B1L	1	3	1	3	2	3	0
Vehicle	E1L1	2	2	0	3	1	0	0
Vehicle	E1L2	3	2	1	3	2	1	0
Vehicle	F5L	2	2	0	3	1	2	0
Clorgyline	B4L	2	2	0	2	1	1	0
Clorgyline	B4R1	2	2	2	2	0	0	0
Clorgyline	B4R2	2	3	1	2	0	0	0
Clorgyline	C4L	3	2	0	3	2	2	0
Clorgyline	C4R	3	2	0	3	1	2	0
Clorgyline	F4L	3	2	2	3	1	2	0
Clorgyline	F4R	3	2	0	3	2	2	0
Clorgyline	G5L	3	2	2	3	1	0	1
Clorgyline	G5R	2	3	2	3	0	2	0
Loratadine	B3L	2	2	0	3	0	2	0
Loratadine	B3R	2	2	2	3	1	0	0
Loratadine	D2L	3	0	0	2	0	3	0
Loratadine	D2R	3	0	0	2	0	0	0
Loratadine	F3L	2	3	1	3	2	2	0
Loratadine	F3R	2	3	0	3	1	0	0
Loratadine	H3L	3	0	0	3	2	2	0
Loratadine	H3R	0	3	0	3	1	0	0
Rasagiline	E4L	2	3	0	3	2	2	0
Rasagiline	G4L	2	3	3	3	2	0	0
Rasagiline	G4R	2	3	0	3	2	0	0
Rasagiline	H1L	2	2	2	3	2	2	0
Rasagiline	H1R	2	3	0	3	2	2	0
Rasagiline	H4L	3	1	2	3	0	3	0
Rasagiline	H4R	3	0	2	3	0	1	1
Rapamycin	C3L	3	0	0	3	1	2	2
Rapamycin	C3R	3	1	1	3	0	0	2
Rapamycin	D1L	3	0	2	3	0	0	2
Rapamycin	D1R	3	1	0	2	0	0	0
Rapamycin	E5L	2	3	0	3	1	0	2
Rapamycin	E5R	2	3	0	3	0	1	1
Rapamycin	G3R	3	0	0	1	0	0	0
Rapamycin	H2R	3	0	0	3	1	0	2
Rapamycin	G3L							
Rapamycin	H2L							
Rapamycin + Clorgyline	C5L	3	2	0	3	0	0	2
Rapamycin + Clorgyline	D5L	3	1	3	3	0	0	2
Rapamycin + Clorgyline	D5R	3	2	2	3	0	0	2
Rapamycin + Clorgyline	E3L	3	1	3	2	0	0	2
Rapamycin + Clorgyline	E3R	3	1	0	3	0	0	0
Rapamycin + Clorgyline	G2R	2	1	3	2	1	0	2
Rapamycin + Clorgyline	C5R							
Rapamycin + Clorgyline	G2L							
Rapamycin + Clorgyline	A4L	3	0	1	0	0	0	1
Rapamycin + Clorgyline	A4R	3	0	0	2	0	0	1
Rapamycin + Clorgyline	A5L	2	1	0	1	1	0	0
Rapamycin + Clorgyline	A5R	3	0	1	1	0	0	0
Rapamycin + Loratadine	A1L	3	0	1	1	1	0	2
Rapamycin + Loratadine	A1R	3	0	0	0	0	0	1
Rapamycin + Loratadine	A3R	3	1	1	2	0	0	3
Rapamycin + Loratadine	B5L	3	0	2	2	0	0	1
Rapamycin + Loratadine	B5R	3	2	1	2	0	1	2
Rapamycin + Loratadine	C1L	3	1	1	3	1	0	2
Rapamycin + Loratadine	C1R	3	1	0	2	0	0	0
Rapamycin + Loratadine	C2L	3	1	2	2	0	0	2
Rapamycin + Loratadine	C2R	2	2	0	2	0	0	3
Rapamycin + Loratadine	G1L	3	0	2	2	0	0	2
Rapamycin + Loratadine	G1R							
Rapamycin + Loratadine	A3L							
Rapamycin + Rasagiline	B2L	3	0	2	1	0	0	1
Rapamycin + Rasagiline	B2R	2	1	1	2	0	0	0
Rapamycin + Rasagiline	D3L	3	2	2	3	1	0	1
Rapamycin + Rasagiline	D3R	2	2	2	3	1	0	2
Rapamycin + Rasagiline	D4L	3	1	2	2	0	1	0
Rapamycin + Rasagiline	D4R	3	0	0	2	0	0	1
Rapamycin + Rasagiline	E2L	3	0	2	3	0	1	2
Rapamycin + Rasagiline	E2R	1	3	0	3	2	0	0
Rapamycin + Rasagiline	F1R	3	1	1	3	1	0	0
Rapamycin + Rasagiline	F2R	3	1	2	2	0	0	2
Rapamycin + Rasagiline	F1L							
Rapamycin + Rasagiline	F2L							

Appendix Table S6. Primers used in RT-PCR assays.

Gene	Forward	Reverse	Species
Actb	GGGGGTTGAGGTGTTGAG	GTCTCAAGTCAGTGTACAGGCC	Mouse
Aldh1a3	CACAGGCTCCATTTGGTGG	CAGCTTTTGAGGAAGAAGCC	Mouse
Aldh2	GACGCCGTCAGCAGGAAAA	CGCCAATCGGTACAACAGC	Mouse
Aldh3a1	GATGCCCATTGTGTGTGTTCG	CCACCGCTTGATGTCTCTGC	Mouse
Aldh3b1	CCTTCTCCAAGAGAAGCCAGG	GGAGAACTTGCCGTGGTACC	Mouse
Aldh3b2	GCTTTGCTGTGATGTTGGGGAGG	TCGCAGTTGTCATCCACATAGC	Mouse
Cat	AGCGACCAGATGAAGCAGTG	TCCGCTCTCTGTCAAAGTGTG	Mouse
Epcam	AGAATACTGTCATTTGCTCCAAACT	GTTCTGGATCGCCCCTTC	Mouse
Fn1	CTGGGACTGTACCTGCATCG	CTCCACTTGTCGCCAATCTT	Mouse
Hrh1	CAAGATGTGTGAGGGGAACAG	CTACCGACAGGCTGACAATGT	Mouse
Maoa	GCCCAGTATCACAGGCCAC	CGGGCTTCCAGAACCAAGA	Mouse
Maob	CCAGAATCATCTCAACAACCAA	TCACTTGACCAGATCCACCA	Mouse
Ppia	CAAATGCTGGACCAAACACAAACG	GTTCATGCCTTCTTTCACCTTCCC	Mouse
Slc22a2	CCACATACATCAGGAATCTTGC	TGAGACGGTAGACCAGGAAAG	Mouse
Slc22a3	ACGACATTACGGAACTTTGG	CAGCCGAAAGAGCAGAAAC	Mouse
Snai1	GAAGCCCAACTATAGCGAGC	AGAGTCCCAGATGAGGGTG	Mouse
Twist1	GGACAAGCTGAGCAAGATTCA	CGGAGAAGGCGTAGCTGAG	Mouse
Vdac1	CCCACATACGCCGATCTTGG	GTGGTTTCCGTGTTGGCAGA	Mouse
Vim	CGTCCACACGCACCTACAG	GGGGGATGAGGAATAGAGGCT	Mouse

Appendix Table S7. Antibodies used in study.

Antibody	Manufacturer	Catalog	Species	Application	Dilution
Anti-ACROLEIN	Abcam	ab48501	Mouse	IHC	1:100
Anti-ACTIN	Santa Cruz	sc-8432	Mouse	WB	1:1000
Anti-ALDH2	Santa Cruz	sc-166362	Mouse	WB	1:500
Anti-CAT	Abcam	ab52477	Rabbit	WB	1:1000
Anti-AKT	Cell Signaling	2920	Mouse	WB	1:1000
Anti-phospho AKT (Ser473)	Cell Signaling	4060	Rabbit	WB	1:500
Anti-DRP1 (Ser616)	Cell Signaling	3455	Rabbit	WB	1:1000
Anti-Ki67	Thermo Scientific	RM-9106-S0	Rabbit	IHC	1:100
Anti-HRH1	Thermo Scientific	PA5-27817	Rabbit	WB	1:1000
				IHC	1:500
Anti-MAO-A	Abcam	ab126751	Rabbit	WB	1:1000
				IHC	1:100
Anti-MAO-B	Thermo Scientific	HPA002328	Rabbit	WB	1:250
				IHC	1:100
Anti-PLCγ1 (Tyr783)	Cell Signaling	2821	Rabbit	WB	1:1000
Anti-ribosomal protein S6	Santa Cruz	sc-74459	Mouse	WB	1:500
Anti-pS6 (Ser235/ 236)	Cell Signaling	2211	Rabbit	WB	1:1000
Anti-αSMA	Sigma-Aldrich	A2547	Mouse	IHC	1:100
Anti-TUBULIN	Abcam	ab44928	Mouse	WB	1:1000
Anti-VDAC1	Abcam	ab15895	Rabbit	WB	1:1000
Anti-VINCULIN	Sigma-Aldrich	V9131	Mouse	WB	1:1000
