# Supplementary material for: Histamine signaling and metabolism identify potential biomarkers and therapies for lymphangioleiomyomatosis
Source: EMBO Mol Med. 2021 Aug 11;13(9):e13929. doi: 10.15252/emmm.202113929 (PMC8422079; doi:10.15252/emmm.202113929)
Supplement: Supplementary file 4 — Source Data for Figure 1 [file EMMM-13-e13929-s003.zip › EMM-2021-13929_Fig1/EMM-2021-13929_Fig1B/EMM-2021-13929_Fig1B.pdf]

Metabolomic Data Analysis with MetaboAnalyst 3.0

User ID: guest5505071873880559094

March 7, 2016

1 Background

MESA or Metabolite Set Enrichment Analysis is a way to identify biologically meaningful patterns that are significantly enriched in quantitative metabolomic data. In conventional approaches, metabolites are evaluated individually for their significance under conditions of study. Those compounds that have passed certain sigificance level are then combined to see if any meaningful patterns can be discerned. In contrast, MSEA directly investigates if a set of functionally related metabolites without the need to preselect compounds based on some arbituary cut-off threshold. It has the potentail to identify subtle but consistent changes among a group of related compounds, which may go undetected with the conventional approaches.

Essentially, MSEA is a metabolomic version of the popular GSEA (Gene Set Enrichment Analysis) software with its own collection of metabolite set libraries as well as an implementation of user-friendly web-interfaces. GSEA is widely used in genomics data analysis and has proven to be a powerful alternative to conventional approaches. For more information, please refer to the original paper by Subramanian A, and a nice review paper by Nam D, Kim SY.

2 MSEA Overview

Metabolite set enrichment analysis consists of four steps - data input, data processing, data analysis, and results download. Different analysis procedures are performed based on different input types. In addition, users can also browse and search the metabolite set libraries as well as upload their self-defined metabolite sets for enrichment analysis. Users can also perform metabolite name mapping between a variety of compound names, synonyms, and major database identifiers.

3 Data Input

There are three enrichment analysis algorithms offered by MSEA. Accordingly, three different types of data inputs are required by these three approaches:

- A list of important compound names - entered as a one column data (*Over Representation Analysis (ORA)*);
- A single measured biofluid (urine, blood, CSF) sample- entered as tab separated two-column data with the first column for compound name, and the second for concentration values (*Single Sample Profiling (SSP)*);
- A compound concentration table - entered as a comma separated (.csv) file with the each sample per row and each metabolite concentration per column. The first column is sample names and the second column for sample phenotype labels (*Quantitative Enrichment Analysis (QEA)*)

You selected Over Representation Analysis (ORA) which requires a list of compound names as input.

4 Data Process

The first step is to standardize the compound labels. It is an essential step since the compound labels will be subsequently compared with compounds contained in the metabolite set library. MSEA has a built-in tool to convert between compound common names, synonyms, identifiers used in HMDB ID, PubChem, ChEBI, BiGG, METLIN, KEGG, or Reactome. **Table 1** shows the conversion results. Note: *1* indicates exact match, *2* indicates approximate match, and *0* indicates no match. A text file contain the result can be found the downloaded file *name_map.csv*

Table 1: Result from Compound Name Mapping

	Query	Match	HMDB	PubChem	KEGG	Comment
1	C00004	NADH	HMDB01487	928	C00004	1
2	C01161	3,4-Dihydroxybenzeneacetic acid	HMDB01336	547	C01161	1
3	C00003	NAD	HMDB00902	5893	C00003	1
4	C04043	3,4-Dihydroxyphenylacetaldehyde	HMDB03791	119219	C04043	1
5	C05580	3,4-Dihydroxymandelic acid	HMDB01866	85782	C05580	1
6	C05577	3,4-Dihydroxymandelaldehyde	HMDB06242	151725	C05577	1
7	C05582	Homovanillic acid	HMDB00118	1738	C05582	1
8	C05581	Homovanillin	HMDB05175	151276	C05581	1
9	C05584	Vanillylmandelic acid	HMDB00291	736172	C05584	1
10	C05583	3-Methoxy-4-hydroxyphenylglycolaldehyde	HMDB04061	440729	C05583	1
11	C00005	NADPH	HMDB00221	22833512	C00005	1
12	C00006	NADP	HMDB00217	5886	C00006	1
13	C00642	p-Hydroxyphenylacetic acid	HMDB00020	127	C00642	1
14	C03765	4-Hydroxyphenylacetaldehyde	HMDB03767	440113	C03765	1
15	C00002	Adenosine triphosphate	HMDB00538	5957	C00002	1
16	C00013	Pyrophosphate	HMDB00250	644102	C00013	1
17	C00575	Cyclic AMP	HMDB00058	6076	C00575	1
18	C04042	20a-Dihydroprogesterone	HMDB03069	92747	C04042	1
19	C00410	Progesterone	HMDB01830	5994	C00410	1
20	C00033	Acetic acid	HMDB00042	176	C00033	1
21	C00084	Acetaldehyde	HMDB00990	177	C00084	1
22	C00160	Glycolic acid	HMDB00115	757	C00160	1
23	C00266	Glycolaldehyde	HMDB03344	756	C00266	1
24	C00818	D-Glucaric acid	HMDB29881	607	C00818	1
25	C02670	D-Glucurono-6,3-lactone	HMDB06355	2724333	C02670	1
26	C00186	L-Lactic acid	HMDB00190	107689	C00186	1
27	C00424	Lactaldehyde	HMDB03052	439231	C00424	1
28	C00256	D-Lactic acid	HMDB01311	61503	C00256	1
29	C00937	D-Lactaldehyde	HMDB06458	439350	C00937	1
30	C00670	Glycerophosphocholine	HMDB00086	71920	C00670	1
31	C04230	LysoPC(16:0)	HMDB10382	460602	C04230	1
32	C05828	Methylimidazoleacetic acid	HMDB02820	75810	C05828	1
33	C05827	Methylimidazole acetaldehyde	HMDB04181	193545	C05827	1
34	C00114	Choline	HMDB00097	305	C00114	1
35	C00157	PC(18:0/18:0)	HMDB08036	94190	C00157	1
36	C00427	Prostaglandin H2	HMDB01381	445049	C00427	1
37	C01312	Prostaglandin I2	HMDB01335	5282411	C01312	1
38	C00219	Arachidonic acid	HMDB01043	444899	C00219	1
39	C07086	Phenylacetic acid	HMDB00209	999	C07086	1
40	C00601	Phenylacetaldehyde	HMDB06236	998	C00601	1
41	C13856	2-Arachidonylglycerol	HMDB04666	5282280	C13856	1
42	C00137	Myoinositol	HMDB00211		C00137	1
43	C01194	1-Phosphatidyl-D-myo-inositol	HMDB06953	53477912	C01194	1
44	C00065	L-Serine	HMDB00187	5951	C00065	1
45	C02737	PS(16:0/16:0)	HMDB00614	3081382	C02737	1
46	C06428	Eicosapentaenoic acid	HMDB01999	446284	C06428	1
47	C03242	8,11,14-Eicosatrienoic acid	HMDB02925	5280581	C03242	1

The second step is to check concentration values. For SSP analysis, the concentration must be measured in *umol* for blood and CSF samples. The urinary concentrations must be first converted to *umol/mmol_creatinine* in order to compare with reported concentrations in literature. No missing or negative values are allowed in SSP analysis. The concentration data for QEA analysis is more flexible. Users can upload either the original concentration data or normalized data. Missing or negative values are allowed (coded as *NA*) for QEA. Please note, MSEA does not perform data normalization. If normalization is important, you should first normalize your data before upload. You can use our companion website **MetaboAnalyst** *www.metaboanalyst.ca* for a variety of data processing and normalization methods.

5 Selection of Metabolite Set Library

Before proceeding to enrichment analysis, a metabolite set library has to be chosen. There are seven built-in libraries offered by MSEA:

- Metabolic pathway associated metabolite sets (*currently contains 88 entries*);
- Disease associated metabolite sets (reported in blood) (*currently contains 416 entries*);
- Disease associated metabolite sets (reported in urine) (*currently contains 346 entries*)
- Disease associated metabolite sets (reported in CSF) (*currently contains 124 entries*)
- Metabolite sets associated with SNPs (*currently contains 4500 entries*)
- Predicted metabolite sets based on computational enzyme knockout model (*currently contains 912 entries*)
- Metabolite sets based on locations (*currently contains 57 entries*)

In addition, MSEA also allows user-defined metabolite sets to be uploaded to perform enrichment analysis on arbitrary groups of compounds which researchers want to test. The metabolite set library is simply a two-column comma separated text file with the first column for metabolite set names and the second column for its comound names (**must use HMDB compound name**) separated by "; ". Please note, the built-in libraries are mainly from human studies. The functional grouping of metabolites may not be valid. Therefore, for data from subjects other than human being, users are suggested to upload their self-defined metabolite set libraries for enrichment analysis.

6 Enrichment Analysis

Over Representation Analysis (ORA) is performed when a list of compound names is provided. The list of compound list can be obtained through conventional feature selection methods, or from a clustering algorithm, or from the compounds with abnormal concentrations detected in SSP, to investigate if some biologically meaningful patterns can be identified.

ORA was implemented using the *hypergeometric test* to evaluate whether a particular metabolite set is represented more than expected by chance within the given compound list. One-tailed p values are provided after adjusting for multiple testing. **Figure 2** below summarizes the result.

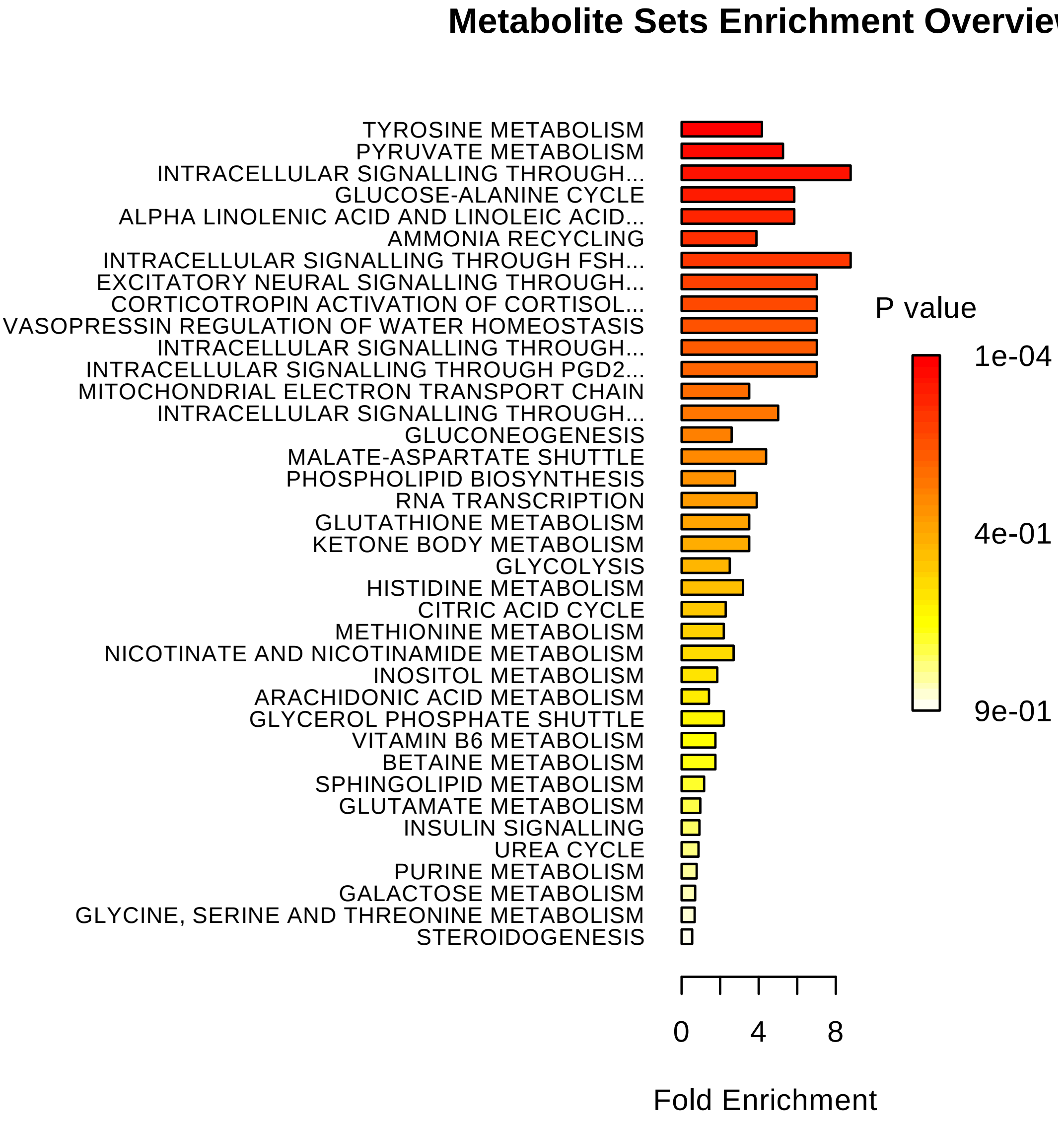

Figure 1: Summary Plot for Over Representation Analysis (ORA)

Table 2: Result from Over Representation Analysis

	total	expected	hits	Raw p	Holm p	FDR
TYROSINE METABOLISM	38	2.16	9	1.36E-04	1.09E-02	1.09E-02
PYRUVATE METABOLISM	20	1.14	6	5.24E-04	4.14E-02	2.09E-02
INTRACELLULAR SIGNALLING THROUGH PROSTACYCLIN RECEPTOR AND PROSTACYCLIN	6	0.34	3	3.08E-03	2.40E-01	6.55E-02
GLUCOSE-ALANINE CYCLE	12	0.68	4	3.28E-03	2.52E-01	6.55E-02
ALPHA LINOLENIC ACID AND LINOLEIC ACID METABOLISM	9	0.51	3	1.14E-02	8.69E-01	1.83E-01
AMMONIA RECYCLING	18	1.03	4	1.57E-02	1.00E+00	1.90E-01
INTRACELLULAR SIGNALLING THROUGH FSH RECEPTOR AND FOLLICLE STIMULATING HORMONE | INTRACELLULAR SIGNALLING THROUGH LHCGR RECEPTOR AND LUTEINIZING HORMONE/CHORIOGONADOTROPIN	4	0.23	2	1.77E-02	1.00E+00	1.90E-01
EXCITATORY NEURAL SIGNALLING THROUGH 5-HTR 4 AND SEROTONIN | EXCITATORY NEURAL SIGNALLING THROUGH 5-HTR 7 AND SEROTONIN | EXCITATORY NEURAL SIGNALLING THROUGH 5-HTR 6 AND SEROTONIN	5	0.28	2	2.85E-02	1.00E+00	1.90E-01
CORTICOTROPIN ACTIVATION OF CORTISOL PRODUCTION	5	0.28	2	2.85E-02	1.00E+00	1.90E-01
VASOPRESSIN REGULATION OF WATER HOMEOSTASIS	5	0.28	2	2.85E-02	1.00E+00	1.90E-01
INTRACELLULAR SIGNALLING THROUGH HISTAMINE H2 RECEPTOR AND HISTAMINE	5	0.28	2	2.85E-02	1.00E+00	1.90E-01
INTRACELLULAR SIGNALLING THROUGH PGD2 RECEPTOR AND PROSTAGLANDIN D2	5	0.28	2	2.85E-02	1.00E+00	1.90E-01
MITOCHONDRIAL ELECTRON TRANSPORT CHAIN	15	0.85	3	4.87E-02	1.00E+00	2.99E-01
INTRACELLULAR SIGNALLING THROUGH ADENOSINE RECEPTOR A2A AND ADENOSINE | INTRACELLULAR SIGNALLING THROUGH ADENOSINE RECEPTOR A2B AND ADENOSINE	7	0.40	2	5.56E-02	1.00E+00	3.17E-01
GLUCONEOGENESIS	27	1.54	4	6.17E-02	1.00E+00	3.29E-01
MALATE-ASPARTATE SHUTTLE	8	0.46	2	7.14E-02	1.00E+00	3.57E-01
PHOSPHOLIPID BIOSYNTHESIS	19	1.08	3	8.84E-02	1.00E+00	3.94E-01
RNA TRANSCRIPTION	9	0.51	2	8.86E-02	1.00E+00	3.94E-01
GLUTATHIONE METABOLISM	10	0.57	2	1.07E-01	1.00E+00	4.27E-01
KETONE BODY METABOLISM	10	0.57	2	1.07E-01	1.00E+00	4.27E-01
GLYCOLYSIS	21	1.20	3	1.12E-01	1.00E+00	4.27E-01
HISTIDINE METABOLISM	11	0.63	2	1.26E-01	1.00E+00	4.58E-01
CITRIC ACID CYCLE	23	1.31	3	1.38E-01	1.00E+00	4.80E-01
METHIONINE METABOLISM	24	1.37	3	1.52E-01	1.00E+00	5.05E-01
NICOTINATE AND NICOTINAMIDE METABOLISM	13	0.74	2	1.66E-01	1.00E+00	5.32E-01
INOSITOL METABOLISM	19	1.08	2	2.95E-01	1.00E+00	9.09E-01
ARACHIDONIC ACID METABOLISM	37	2.11	3	3.54E-01	1.00E+00	1.00E+00
GLYCEROL PHOSPHATE SHUTTLE	8	0.46	1	3.76E-01	1.00E+00	1.00E+00
VITAMIN B6 METABOLISM	10	0.57	1	4.46E-01	1.00E+00	1.00E+00
BETAINE METABOLISM	10	0.57	1	4.46E-01	1.00E+00	1.00E+00
SPHINGOLIPID METABOLISM	15	0.85	1	5.88E-01	1.00E+00	1.00E+00
GLUTAMATE METABOLISM	18	1.03	1	6.56E-01	1.00E+00	1.00E+00
INSULIN SIGNALLING	19	1.08	1	6.76E-01	1.00E+00	1.00E+00
UREA CYCLE	20	1.14	1	6.95E-01	1.00E+00	1.00E+00
PURINE METABOLISM	45	2.56	2	7.43E-01	1.00E+00	1.00E+00
GALACTOSE METABOLISM	25	1.42	1	7.74E-01	1.00E+00	1.00E+00
GLYCINE, SERINE AND THREONINE METABOLISM	26	1.48	1	7.88E-01	1.00E+00	1.00E+00
STEROIDOGENESIS	32	1.82	1	8.53E-01	1.00E+00	1.00E+00

The report was generated on Mon Mar 7 12:36:58 2016 with R version 3.2.2 (2015-08-14). Thank you for using MetaboAnalyst! For suggestions and feedback please contact Jeff Xia (*jeff.xia@mcgill.ca*).
