# Supplementary figures and images for: Histamine signaling and metabolism identify potential biomarkers and therapies for lymphangioleiomyomatosis
Source: EMBO Mol Med. 2021 Aug 11;13(9):e13929. doi: 10.15252/emmm.202113929 (PMC8422079; doi:10.15252/emmm.202113929)

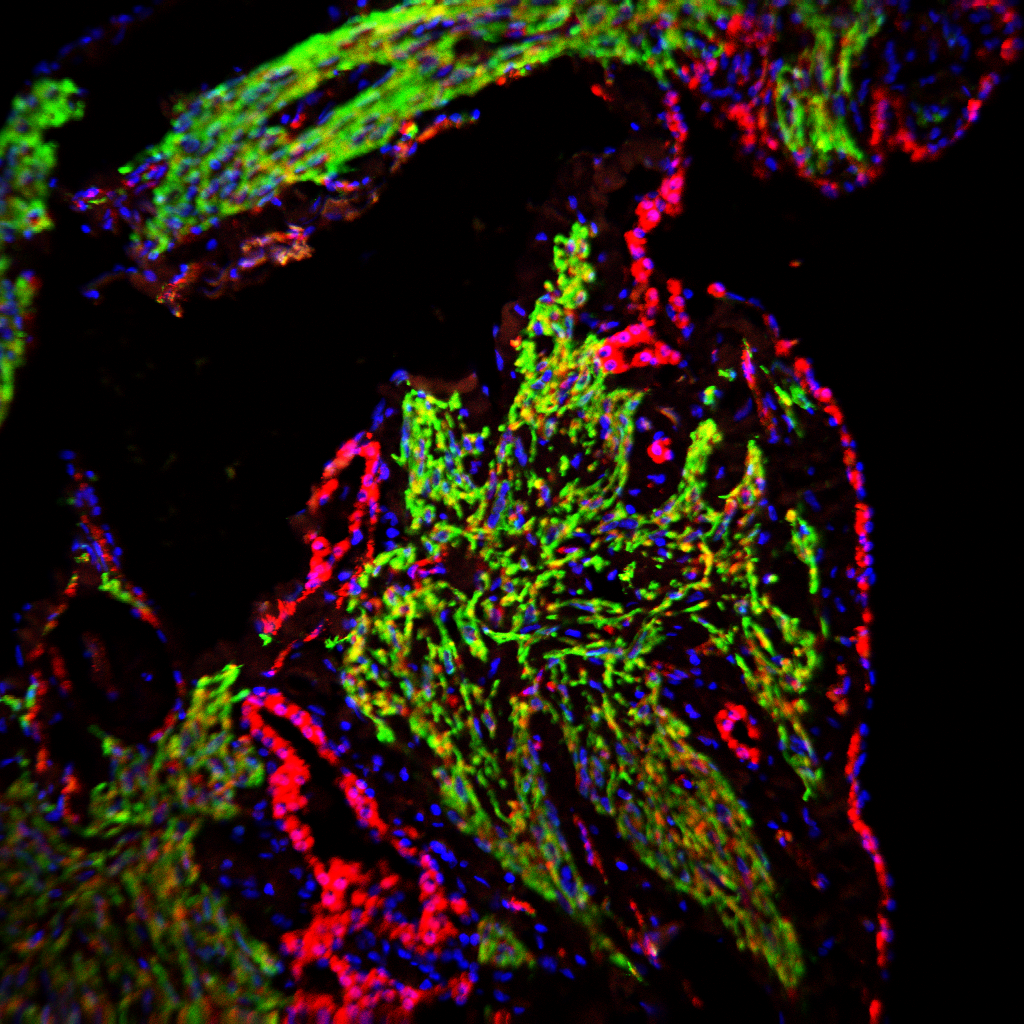

Supplement: Supplementary file 6 — Source Data for Figure 3 [file EMMM-13-e13929-s005.zip › EMM-2021-13929_Fig3/EMM-2021-13929_Fig3B/EMM-2021-13929_Fig3B_2018-05-25-T08-20879-A5-SMA+MAOA-20X1_z1.tif]

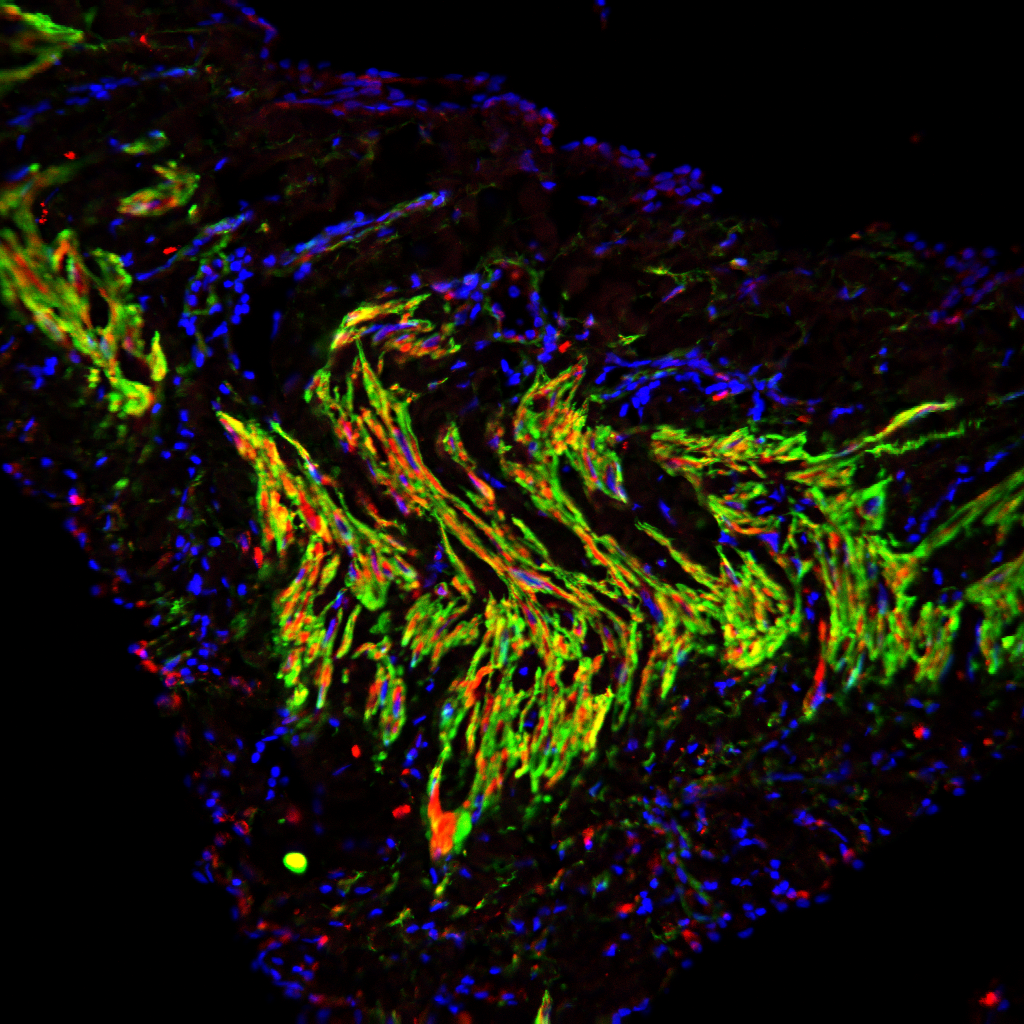

Supplement: Supplementary file 6 — Source Data for Figure 3 [file EMMM-13-e13929-s005.zip › EMM-2021-13929_Fig3/EMM-2021-13929_Fig3B/EMM-2021-13929_Fig3B_2018-05-25-T08-20879-A5-SMA+MAOB-20X_z1.tif]

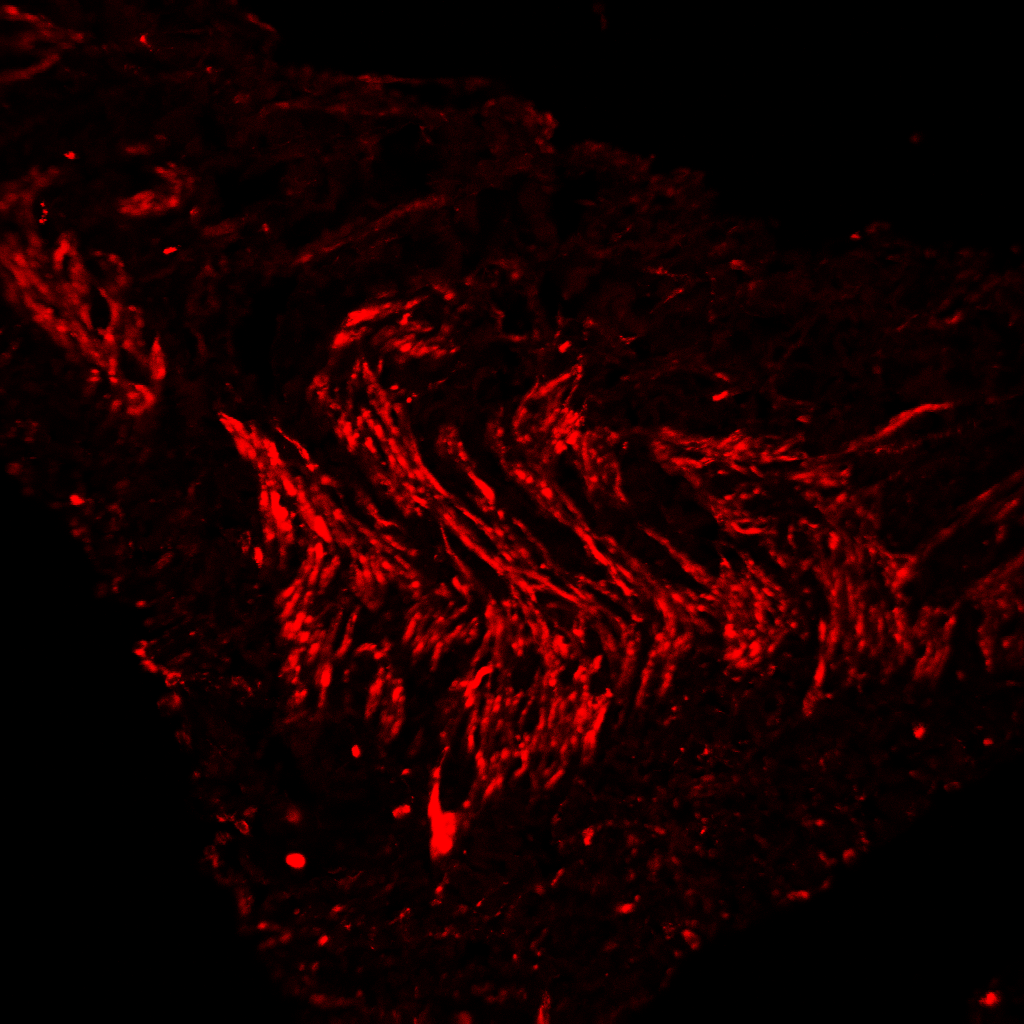

Supplement: Supplementary file 6 — Source Data for Figure 3 [file EMMM-13-e13929-s005.zip › EMM-2021-13929_Fig3/EMM-2021-13929_Fig3B/EMM-2021-13929_Fig3B_2018-05-25-T08-20879-A5-SMA+MAOB-20X_z1_ch02.tif]

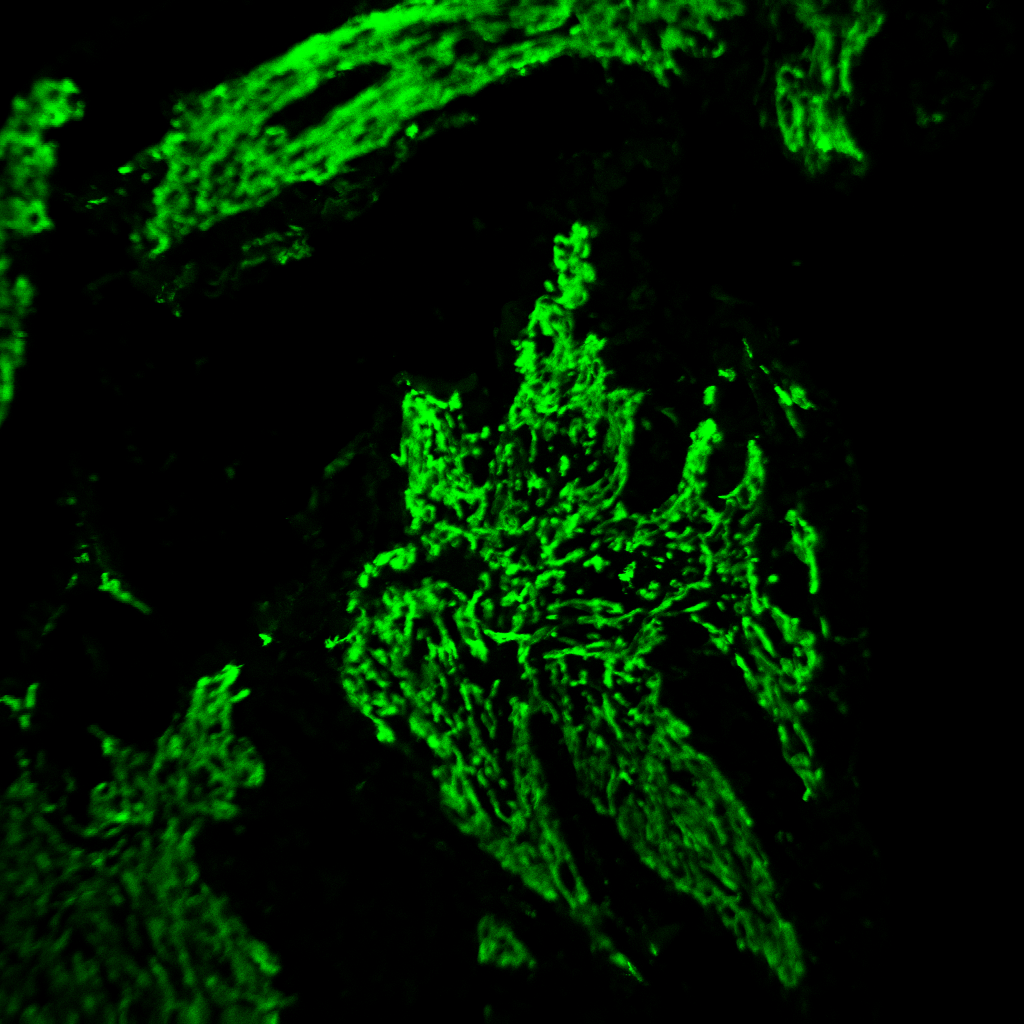

Supplement: Supplementary file 6 — Source Data for Figure 3 [file EMMM-13-e13929-s005.zip › EMM-2021-13929_Fig3/EMM-2021-13929_Fig3B/EMM-2021-13929_Fig3B_2018-05-25-T08-20879-A5-SMA+MAOA-20X1_z1_ch01.tif]

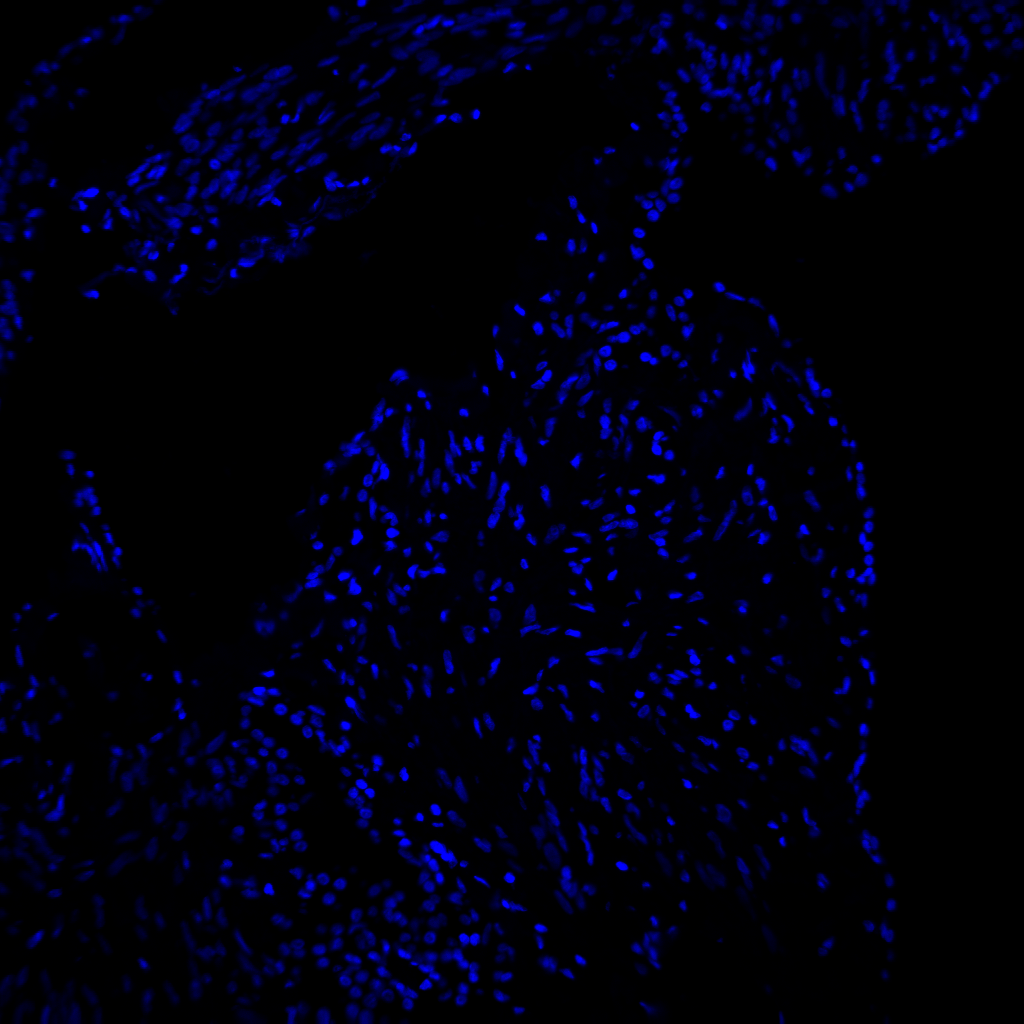

Supplement: Supplementary file 6 — Source Data for Figure 3 [file EMMM-13-e13929-s005.zip › EMM-2021-13929_Fig3/EMM-2021-13929_Fig3B/EMM-2021-13929_Fig3B_2018-05-25-T08-20879-A5-SMA+MAOA-20X1_z1_ch00.tif]

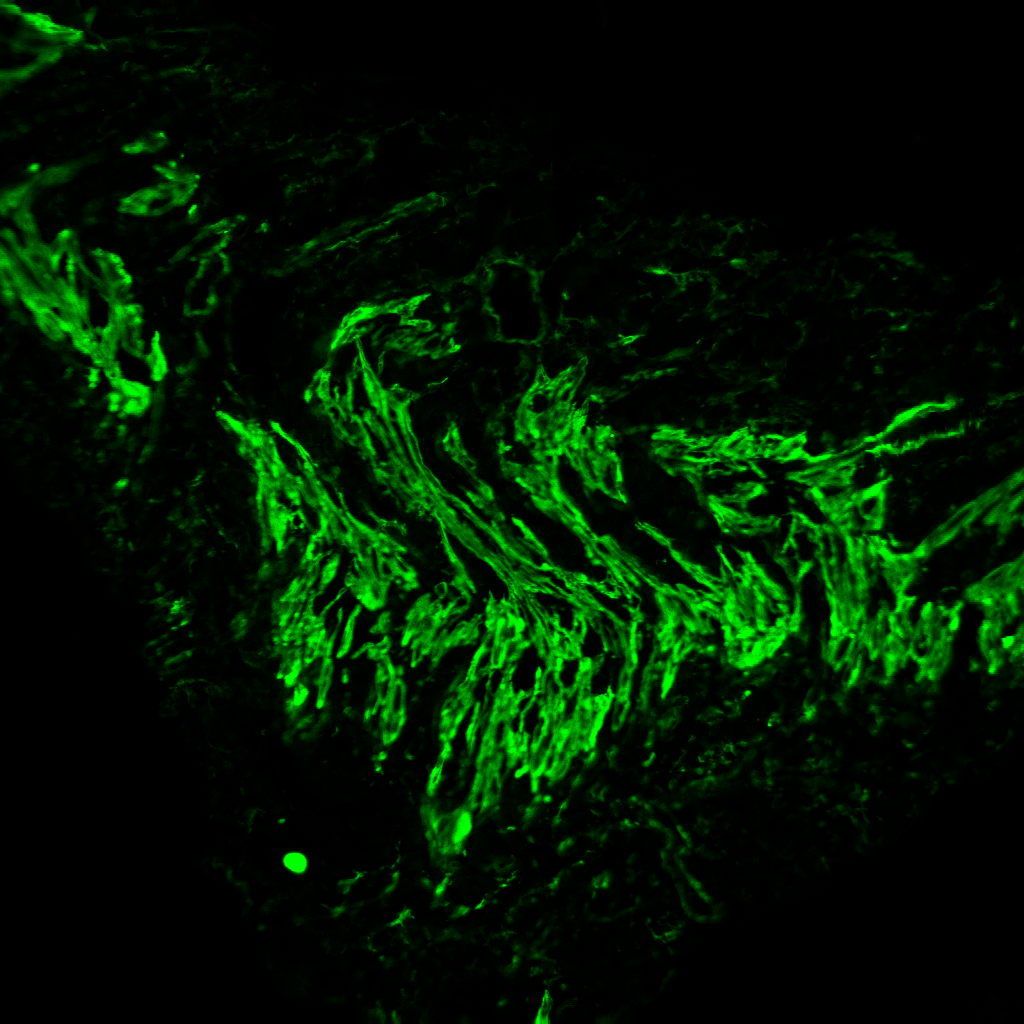

Supplement: Supplementary file 6 — Source Data for Figure 3 [file EMMM-13-e13929-s005.zip › EMM-2021-13929_Fig3/EMM-2021-13929_Fig3B/EMM-2021-13929_Fig3B_2018-05-25-T08-20879-A5-SMA+MAOB-20X_z1_ch01.tif]

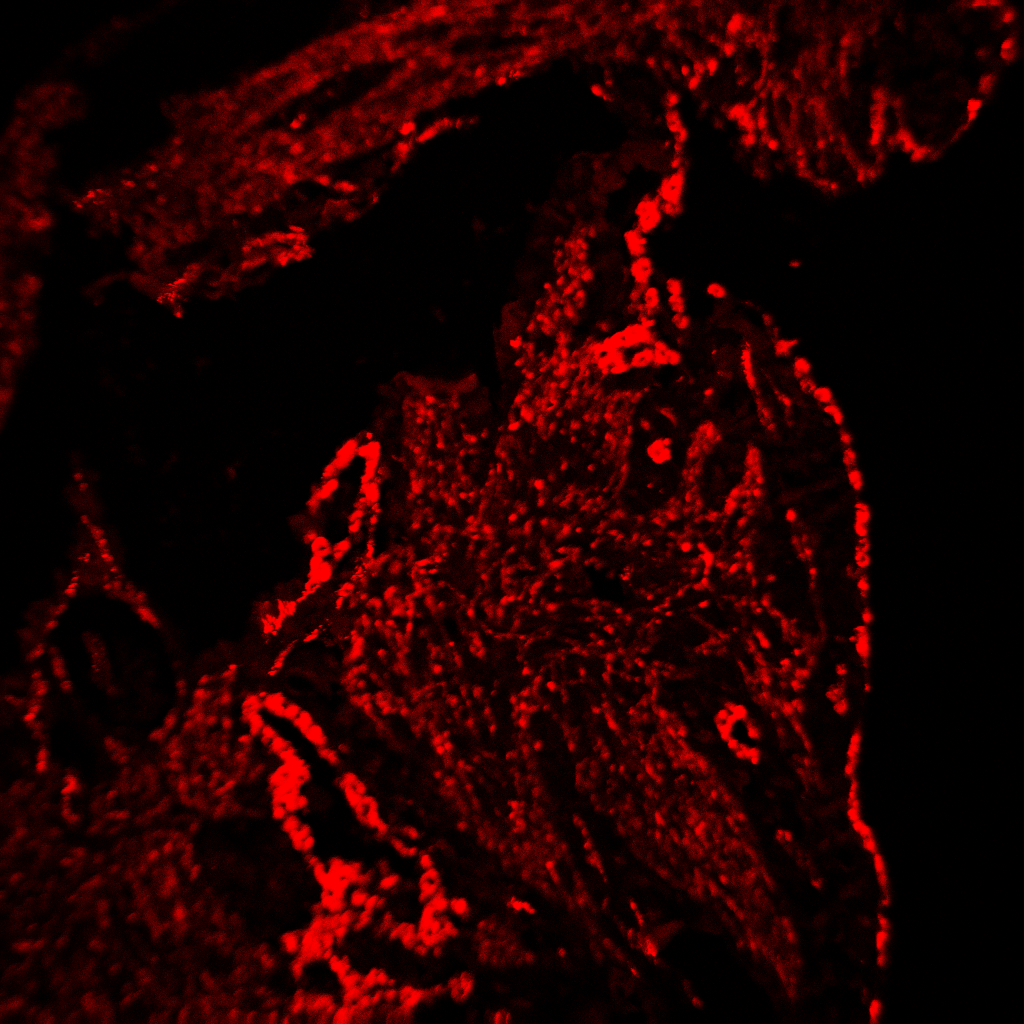

Supplement: Supplementary file 6 — Source Data for Figure 3 [file EMMM-13-e13929-s005.zip › EMM-2021-13929_Fig3/EMM-2021-13929_Fig3B/EMM-2021-13929_Fig3B_2018-05-25-T08-20879-A5-SMA+MAOA-20X1_z1_ch02.tif]

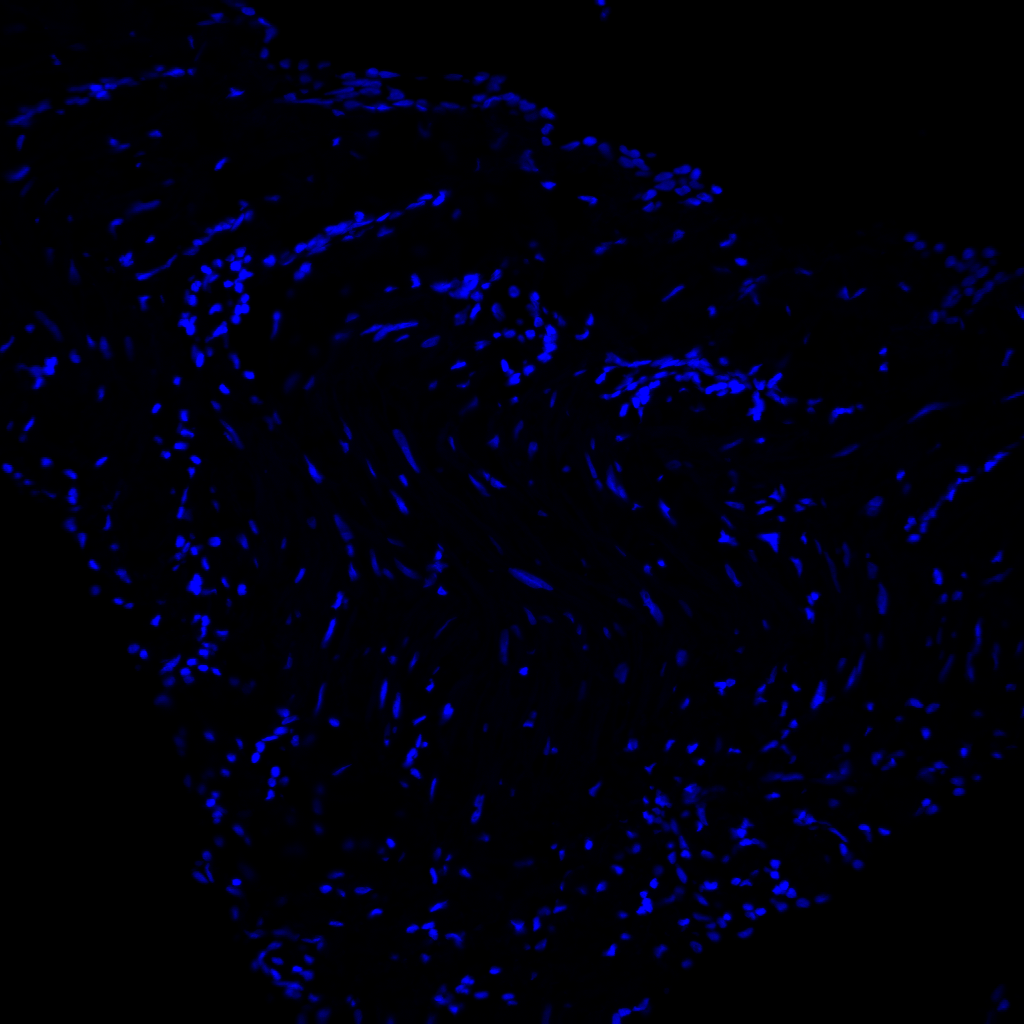

Supplement: Supplementary file 6 — Source Data for Figure 3 [file EMMM-13-e13929-s005.zip › EMM-2021-13929_Fig3/EMM-2021-13929_Fig3B/EMM-2021-13929_Fig3B_2018-05-25-T08-20879-A5-SMA+MAOB-20X_z1_ch00.tif]

## Data Set 2: FC17278 2017.12.13 ALDH +++ 014 • Event Count: 21.779

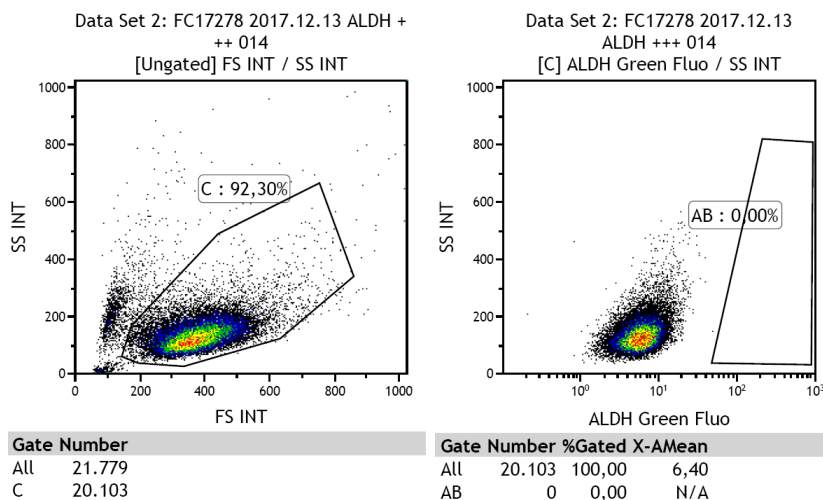

## Data Set 4: FC17278 2017.12.13 ALDH --- 016 • Event Count: 22.627

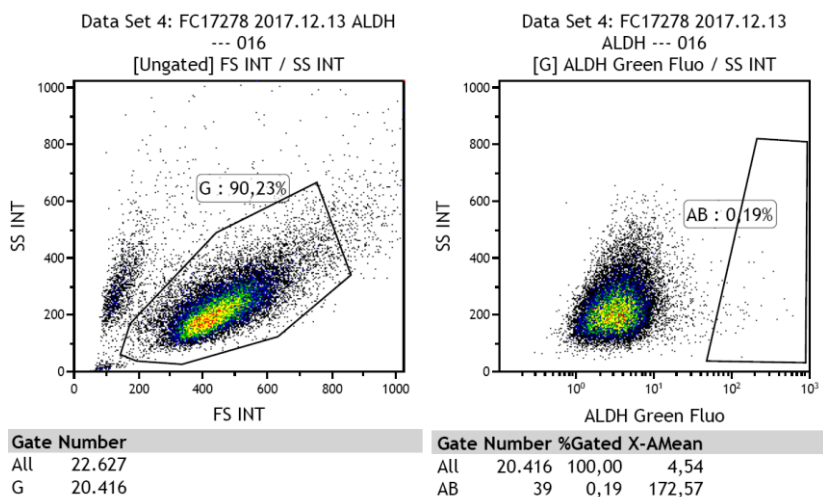

Supplement: Supplementary file 6 — Source Data for Figure 3 [file EMMM-13-e13929-s005.zip › EMM-2021-13929_Fig3/EMM-2021-13929_Fig3E/EMM-2021-13929_Fig3_2017-12-13_ALDHFLUOR.pdf]

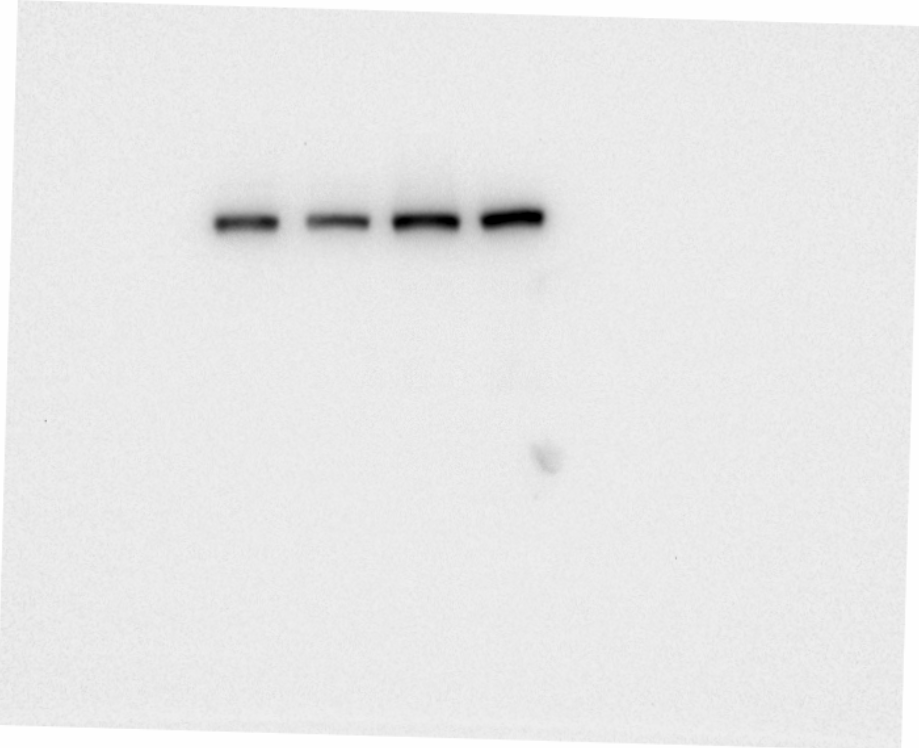

Supplement: Supplementary file 6 — Source Data for Figure 3 [file EMMM-13-e13929-s005.zip › EMM-2021-13929_Fig3/EMM-2021-13929_Fig3D/EMM-2021-13929_Fig3D_right_MAOA.tif]

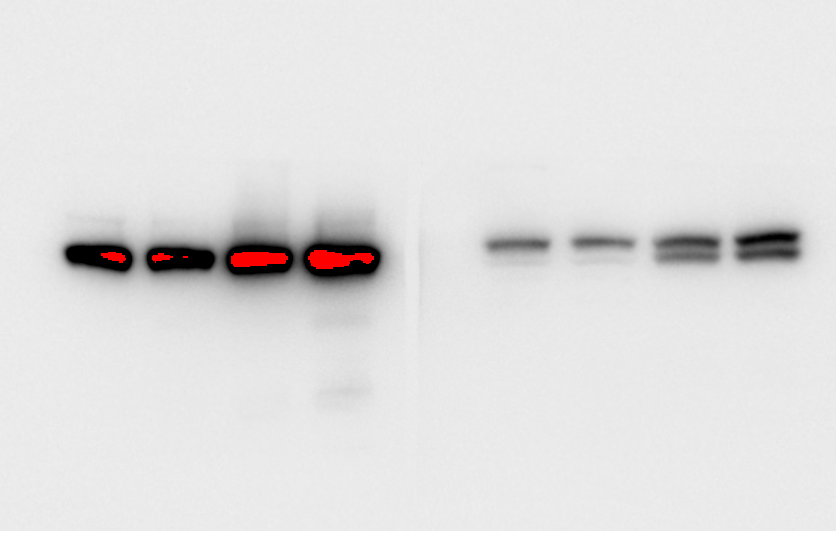

Supplement: Supplementary file 6 — Source Data for Figure 3 [file EMMM-13-e13929-s005.zip › EMM-2021-13929_Fig3/EMM-2021-13929_Fig3D/EMM-2021-13929_Fig3D_right_MAOB.tif]

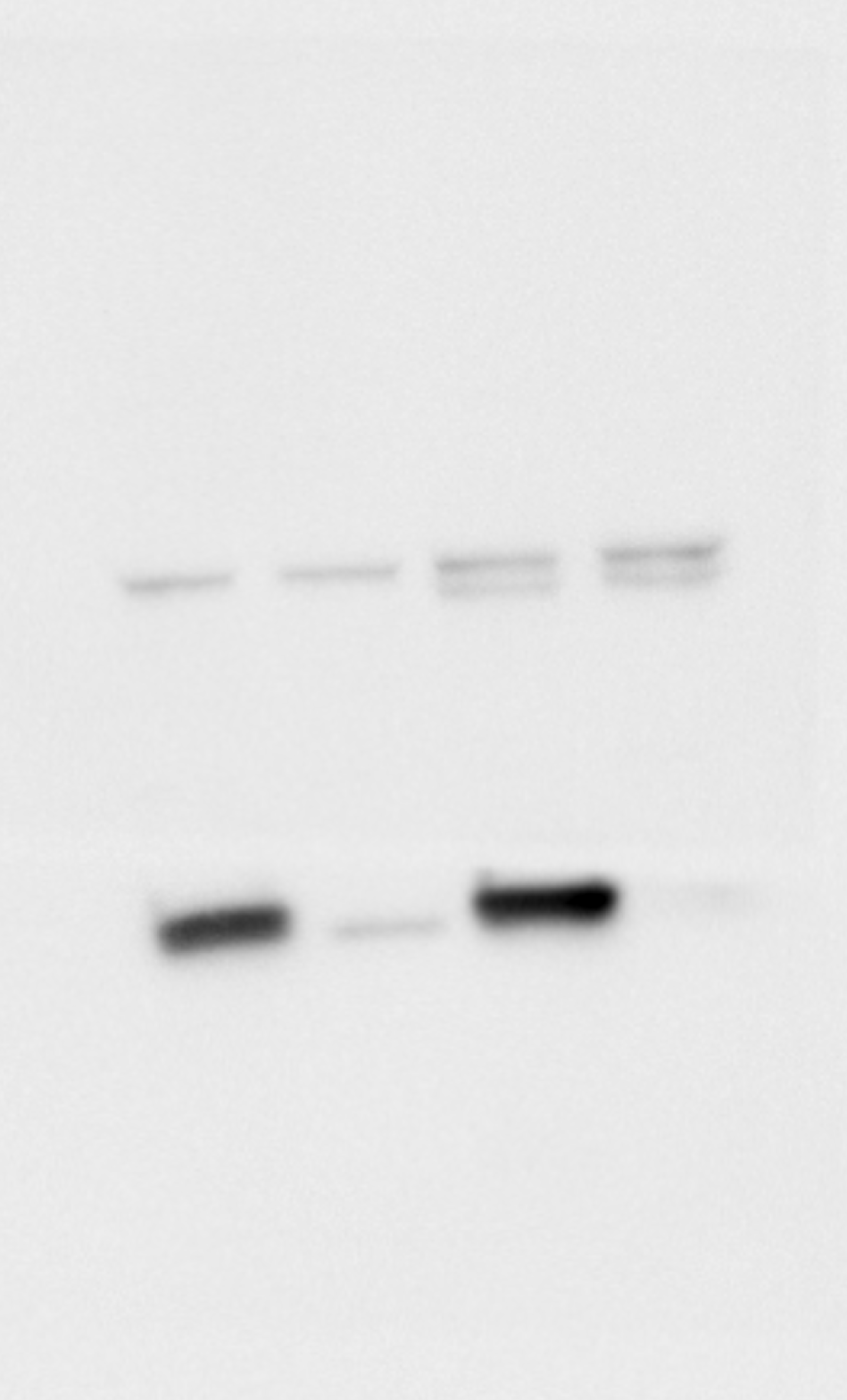

Supplement: Supplementary file 6 — Source Data for Figure 3 [file EMMM-13-e13929-s005.zip › EMM-2021-13929_Fig3/EMM-2021-13929_Fig3D/EMM-2021-13929_Fig3D_left_pS6.tif]

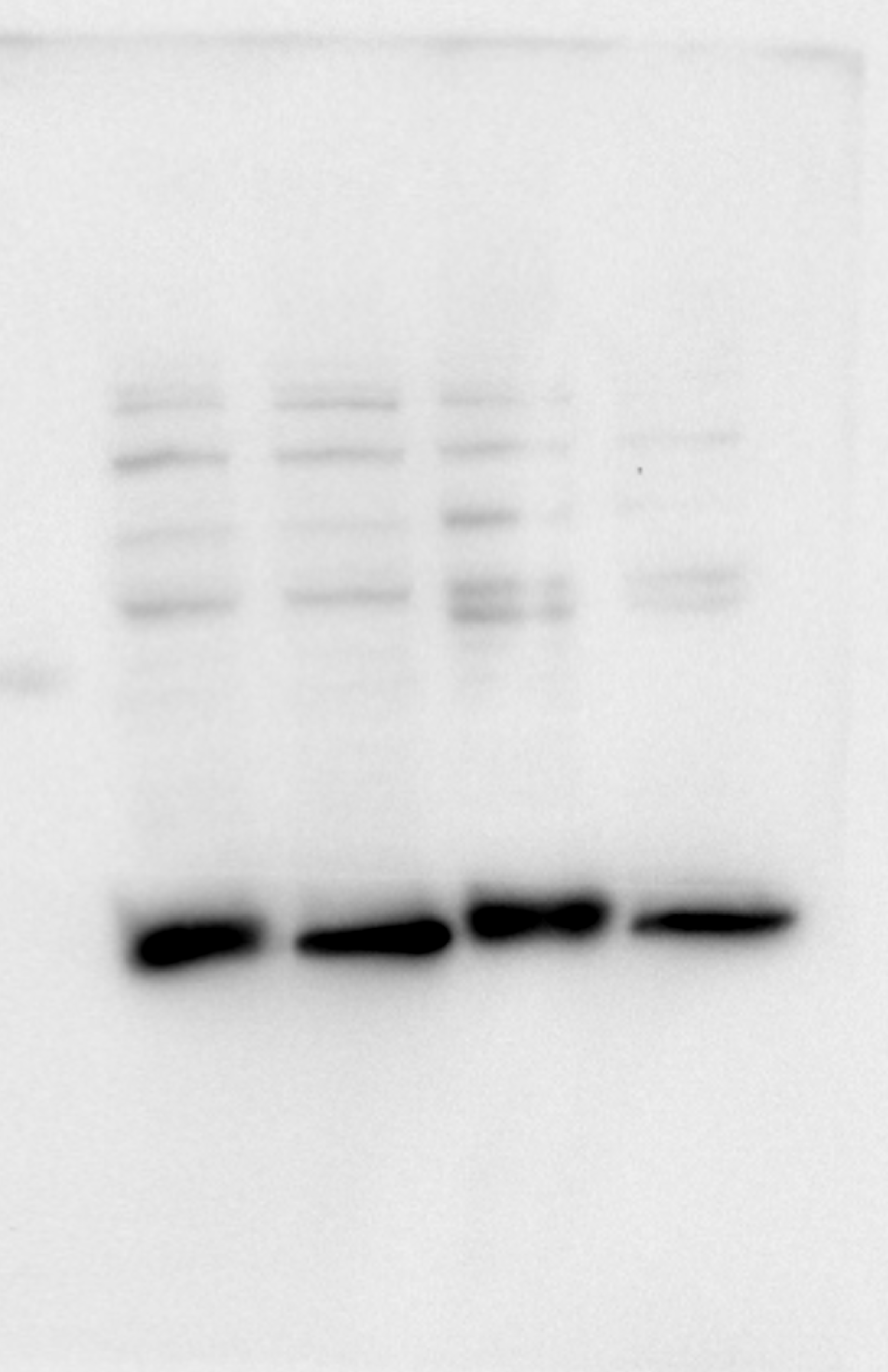

Supplement: Supplementary file 6 — Source Data for Figure 3 [file EMMM-13-e13929-s005.zip › EMM-2021-13929_Fig3/EMM-2021-13929_Fig3D/EMM-2021-13929_Fig3D_left_total-S6.tif]

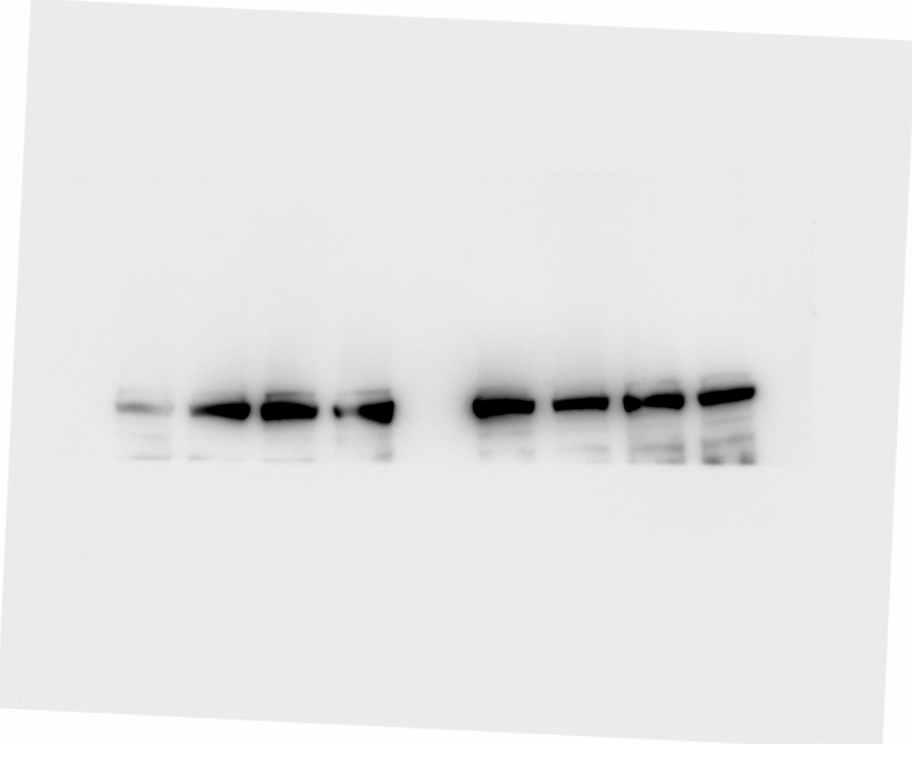

Supplement: Supplementary file 6 — Source Data for Figure 3 [file EMMM-13-e13929-s005.zip › EMM-2021-13929_Fig3/EMM-2021-13929_Fig3D/EMM-2021-13929_Fig3D_right_ACTIN.tif]

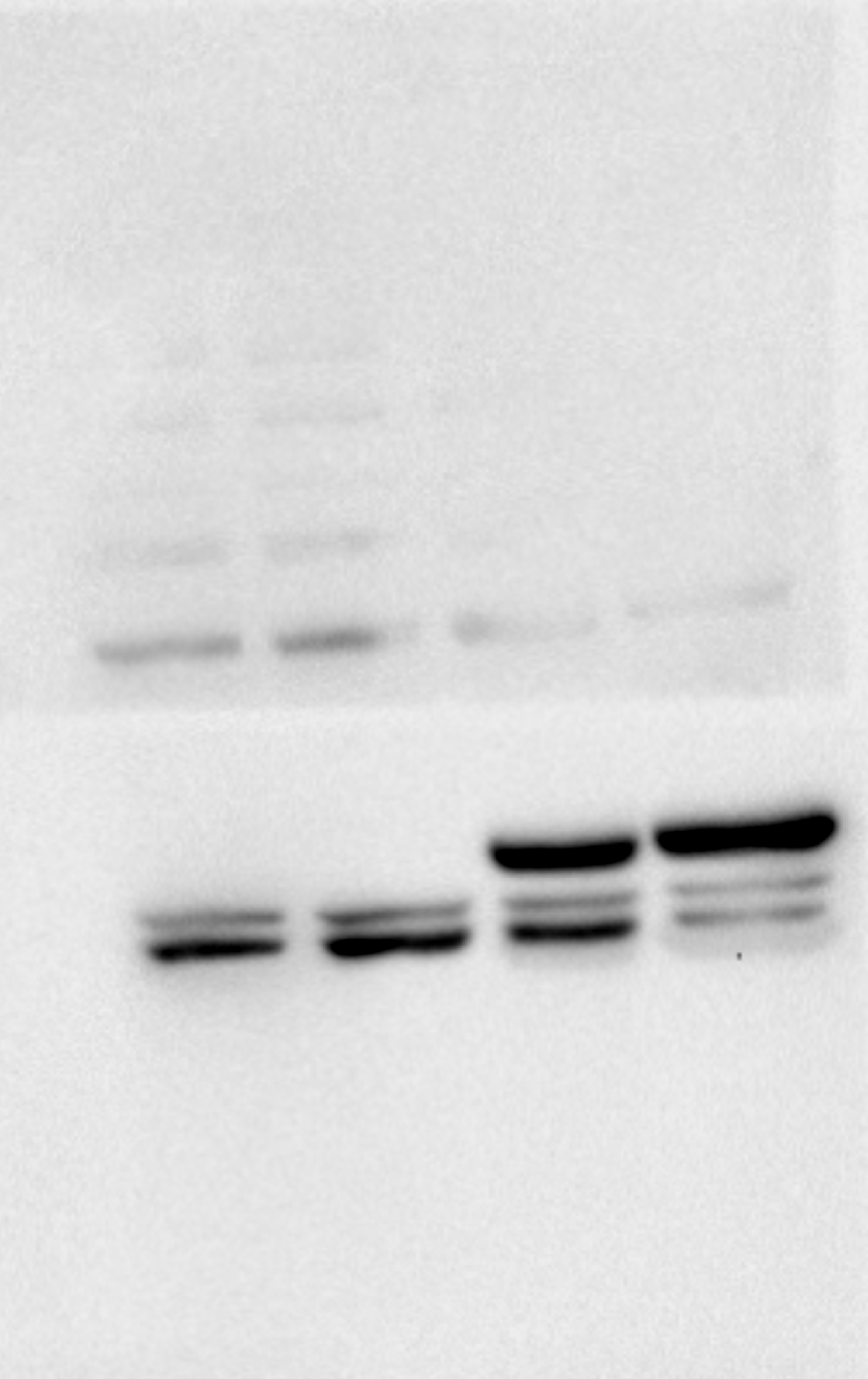

Supplement: Supplementary file 6 — Source Data for Figure 3 [file EMMM-13-e13929-s005.zip › EMM-2021-13929_Fig3/EMM-2021-13929_Fig3D/EMM-2021-13929_Fig3D_left_ALDH2.tif]

## Slide 1
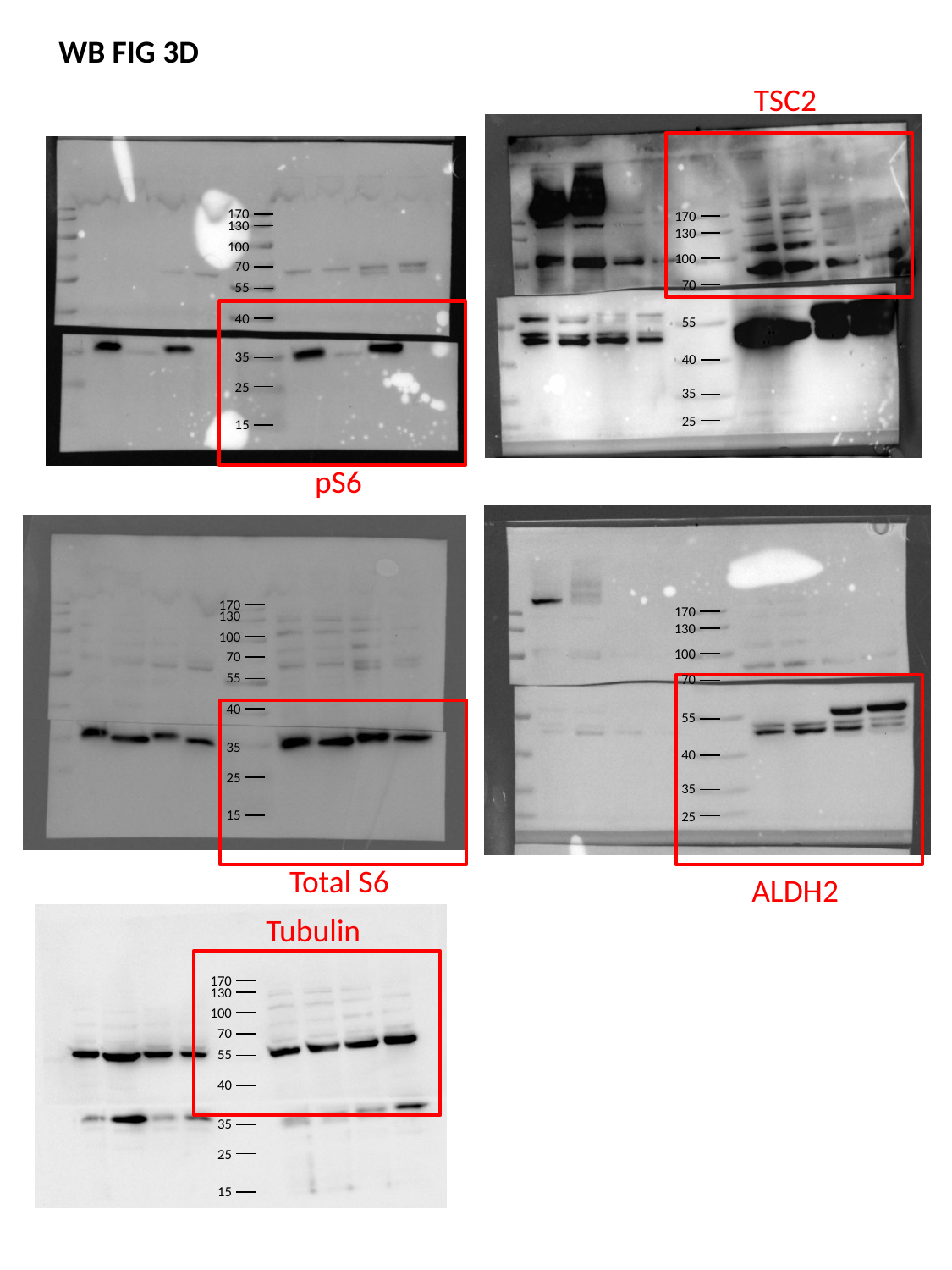

WB FIG 3D
TSC2
170
130
100
70
55
40
35
25
15
170
130
100
70
55
40
35
25
pS6
170
130
100
70
55
40
35
25
15
170
130
100
70
55
40
35
25
Total S6
ALDH2
Tubulin
170
130
100
70
55
40
35
25
15

Supplement: Supplementary file 6 — Source Data for Figure 3 [file EMMM-13-e13929-s005.zip › EMM-2021-13929_Fig3/EMM-2021-13929_Fig3D/EMM-2021-13929_Fig3D-left_withMARKERS_Total-pS6-ALDH2-TSC2-Tubulin.pptx]

## Slide 1
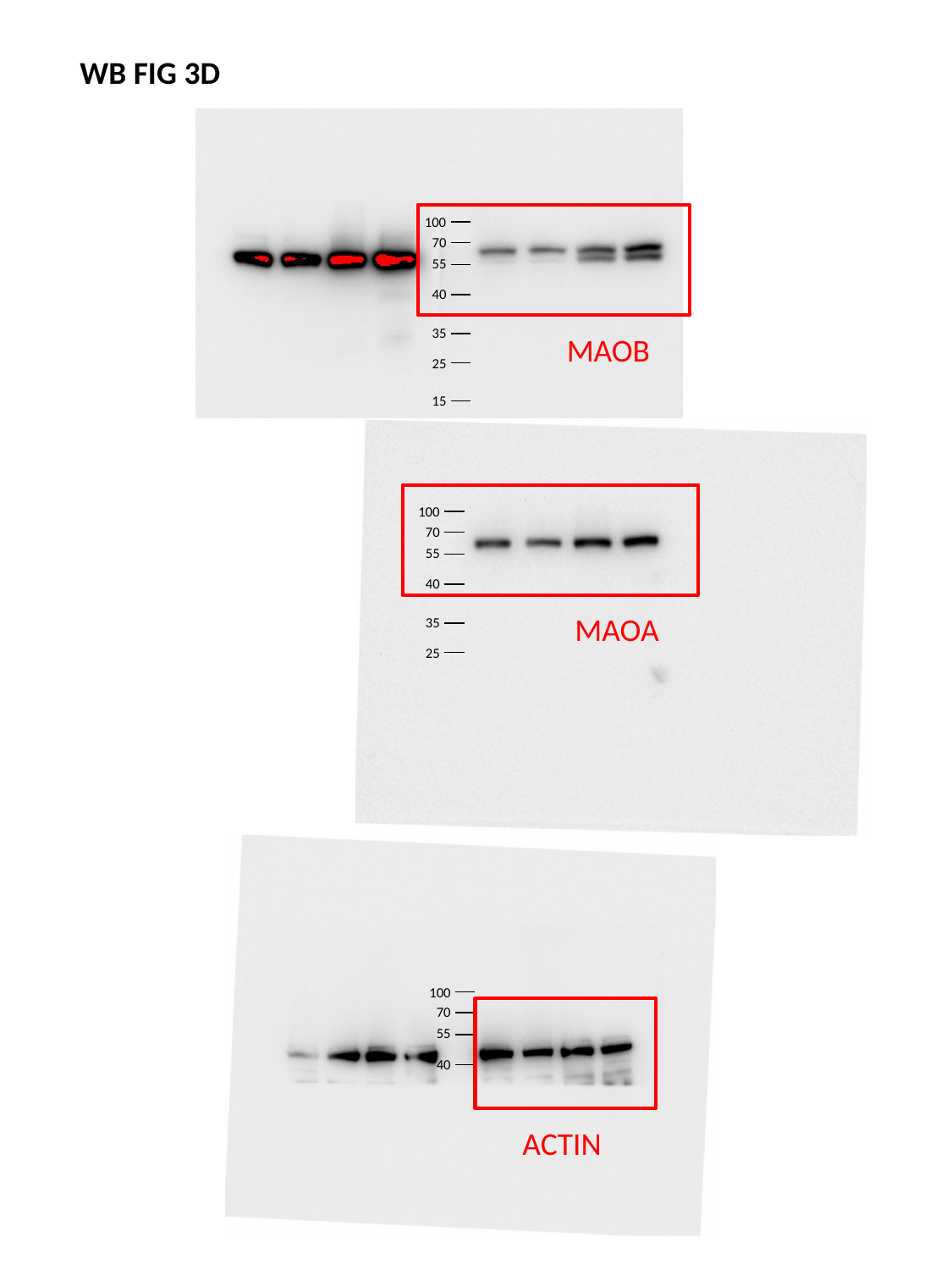

WB FIG 3D
100
70
55
40
35
25
15
MAOB
100
70
55
40
35
25
MAOA
100
70
55
40
ACTIN

Supplement: Supplementary file 6 — Source Data for Figure 3 [file EMMM-13-e13929-s005.zip › EMM-2021-13929_Fig3/EMM-2021-13929_Fig3D/EMM-2021-13929_Fig3D-right_withMARKERS_MAOA-MAOB-Actin.pptx]

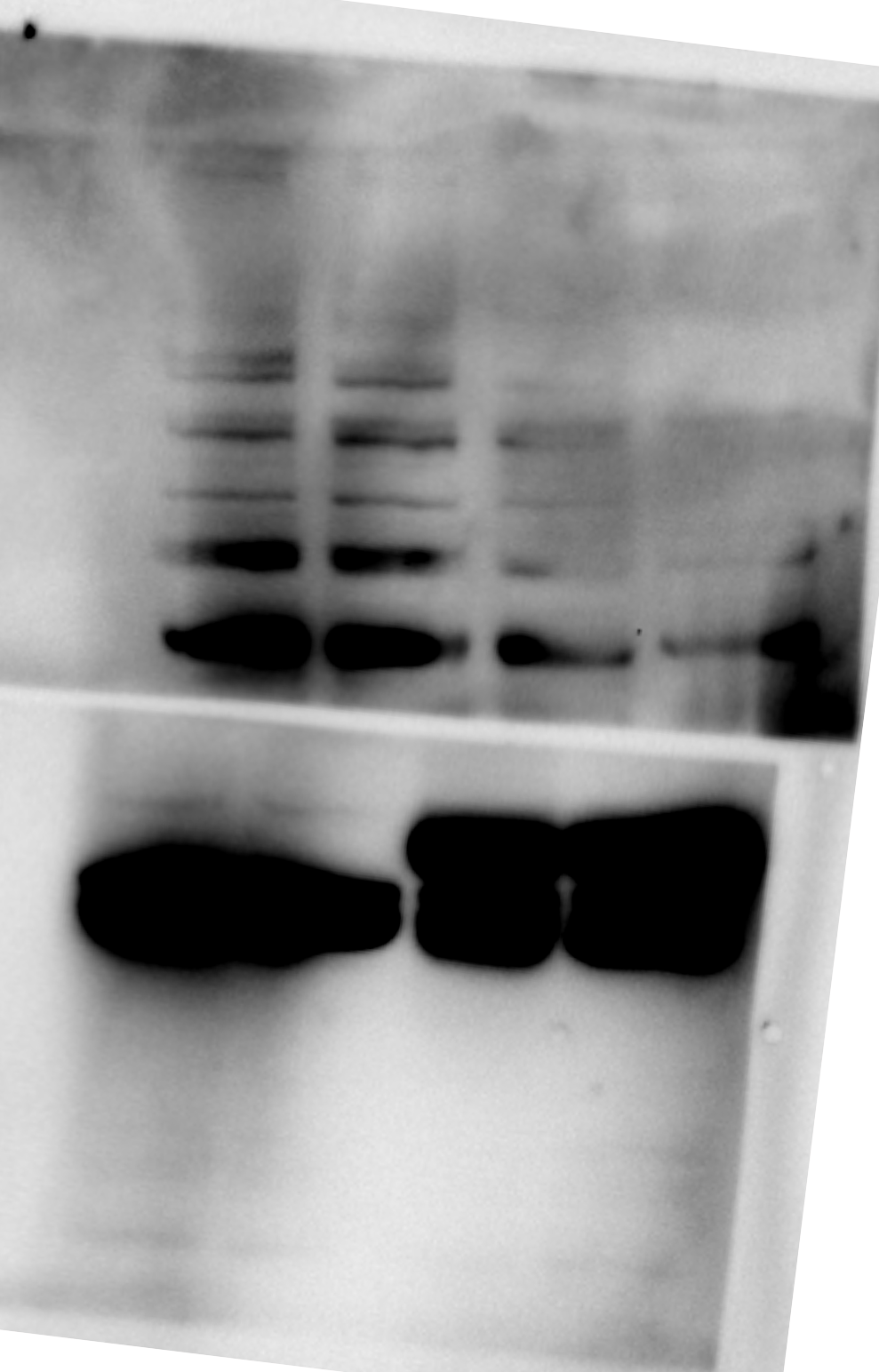

Supplement: Supplementary file 6 — Source Data for Figure 3 [file EMMM-13-e13929-s005.zip › EMM-2021-13929_Fig3/EMM-2021-13929_Fig3D/EMM-2021-13929_Fig3D_left_TSC2.tif]

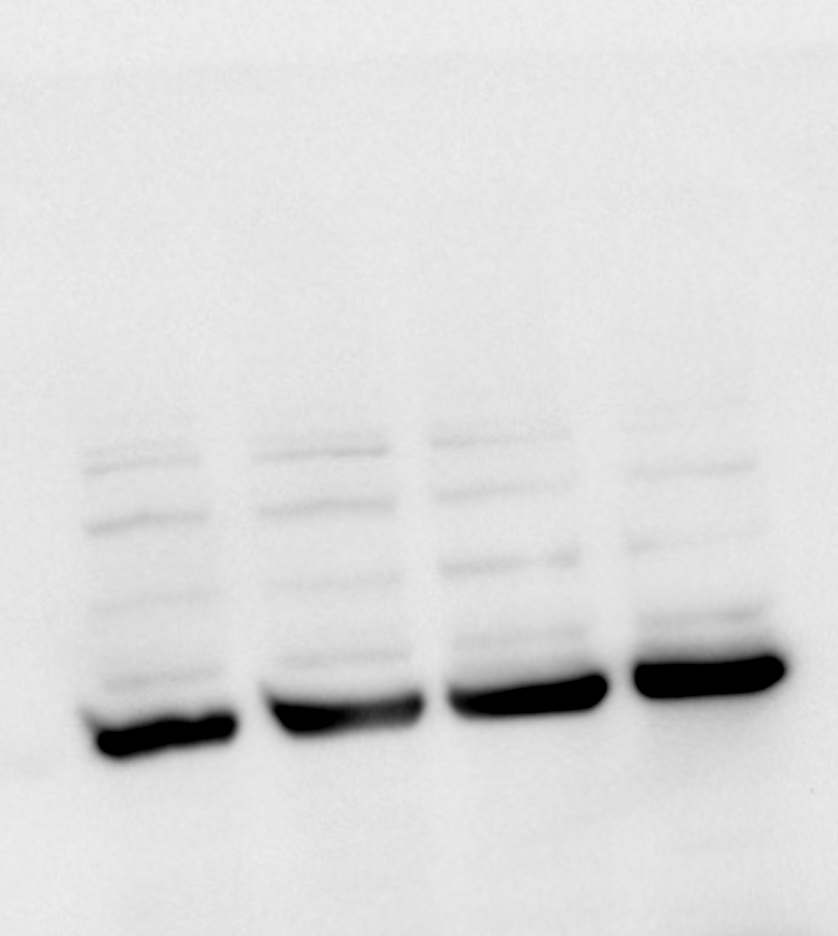

Supplement: Supplementary file 6 — Source Data for Figure 3 [file EMMM-13-e13929-s005.zip › EMM-2021-13929_Fig3/EMM-2021-13929_Fig3D/EMM-2021-13929_Fig3D_left_TUBULIN.tif]

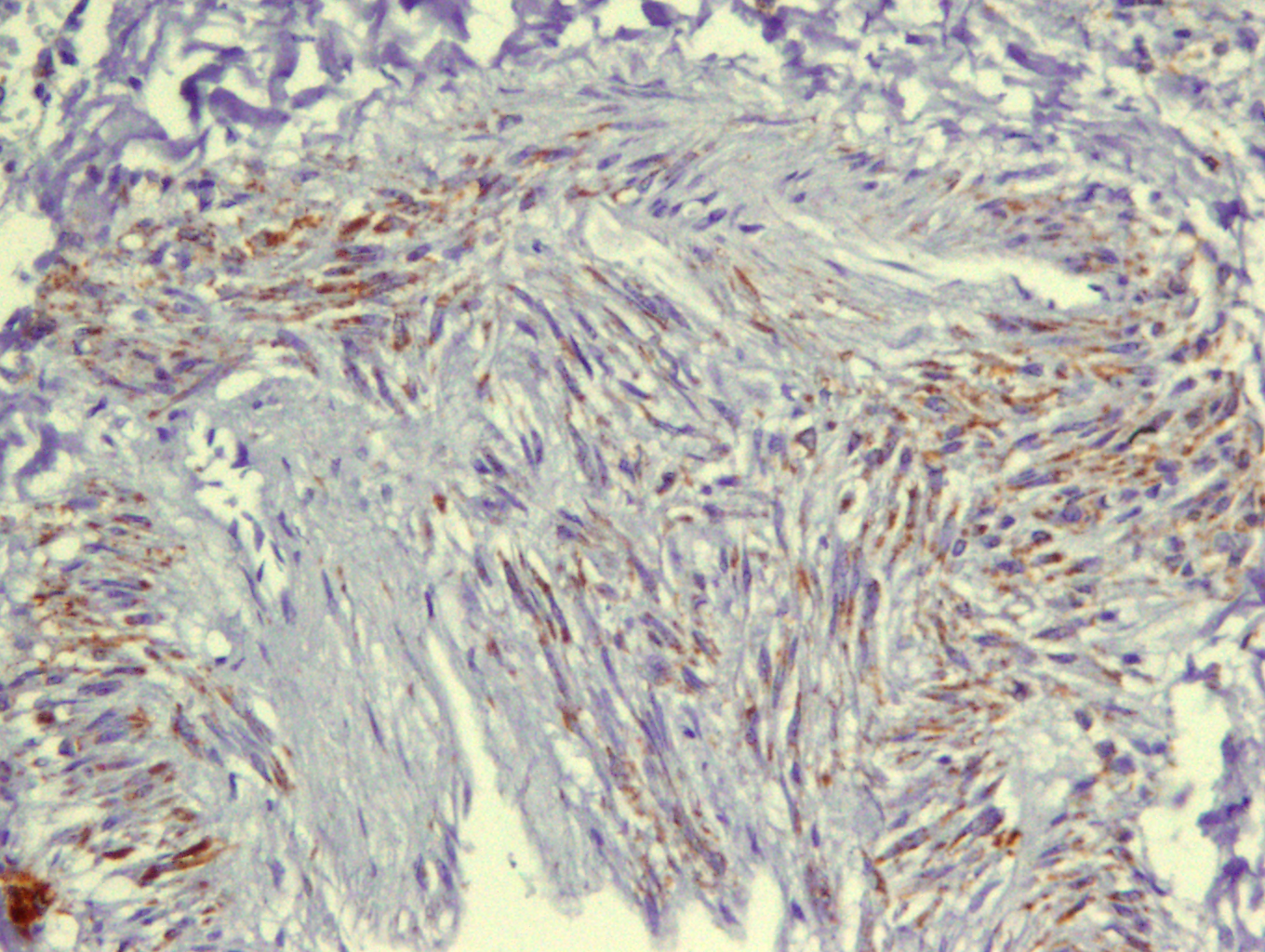

Supplement: Supplementary file 6 — Source Data for Figure 3 [file EMMM-13-e13929-s005.zip › EMM-2021-13929_Fig3/EMM-2021-13929_Fig3A/EMM-2021-13929_Fig3A_LAM_MAOB_T09-6A3_20X.tif]

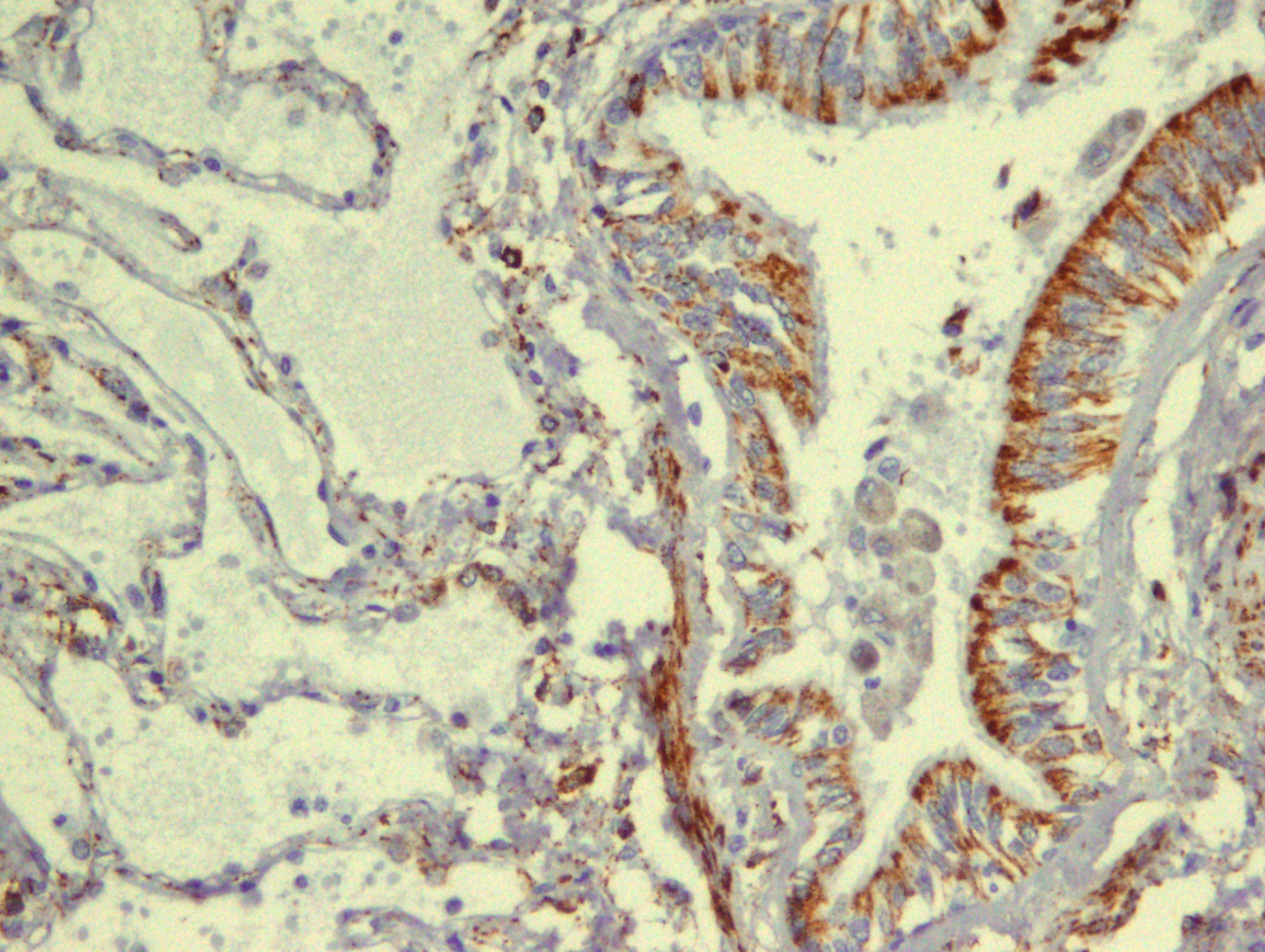

Supplement: Supplementary file 6 — Source Data for Figure 3 [file EMMM-13-e13929-s005.zip › EMM-2021-13929_Fig3/EMM-2021-13929_Fig3A/EMM-2021-13929_Fig3A_Normal-Lung_MAOB_10X.tif]

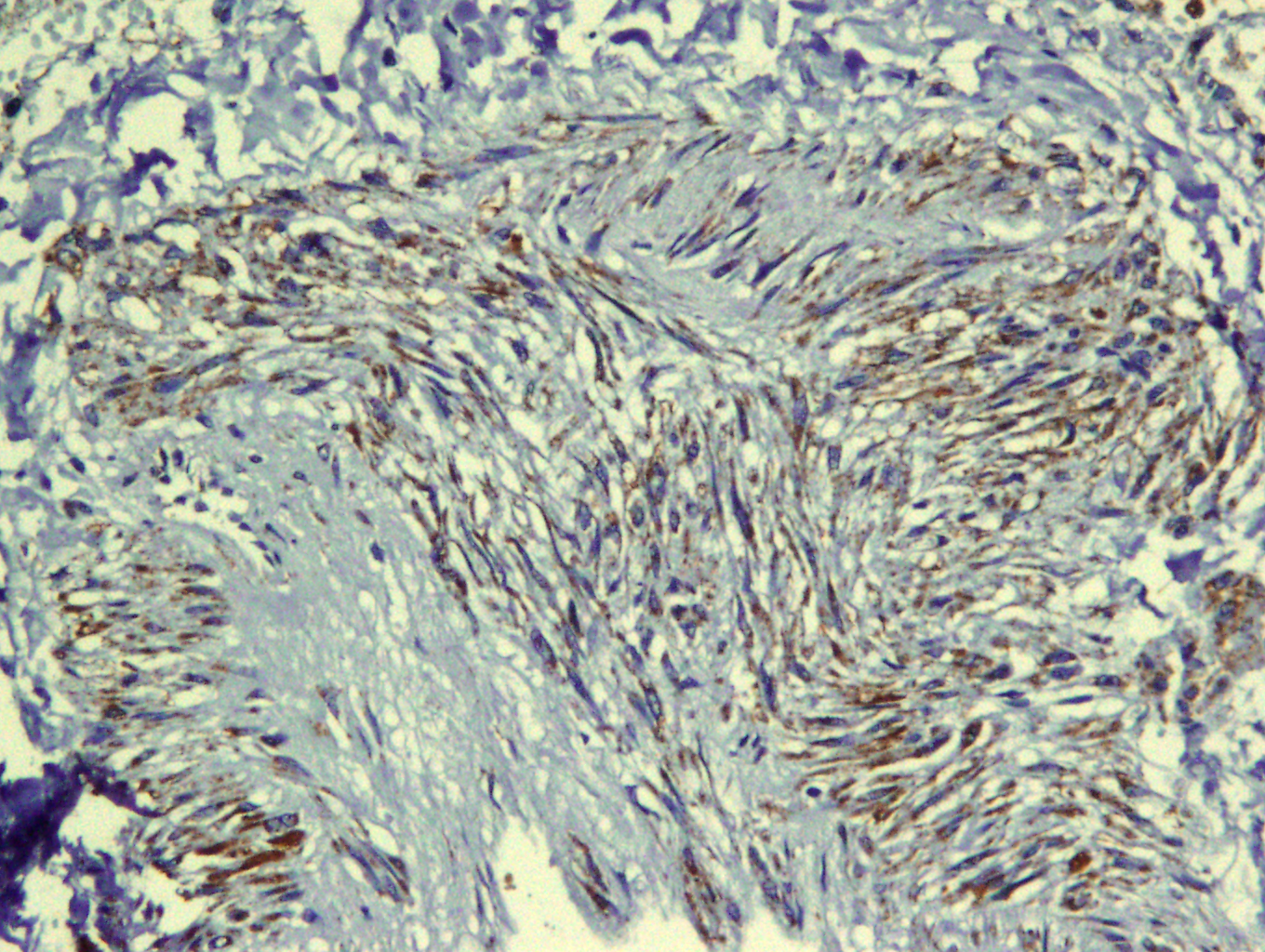

Supplement: Supplementary file 6 — Source Data for Figure 3 [file EMMM-13-e13929-s005.zip › EMM-2021-13929_Fig3/EMM-2021-13929_Fig3A/EMM-2021-13929_Fig3A_LAM_MAOA_T09-6A3_20X.tif]

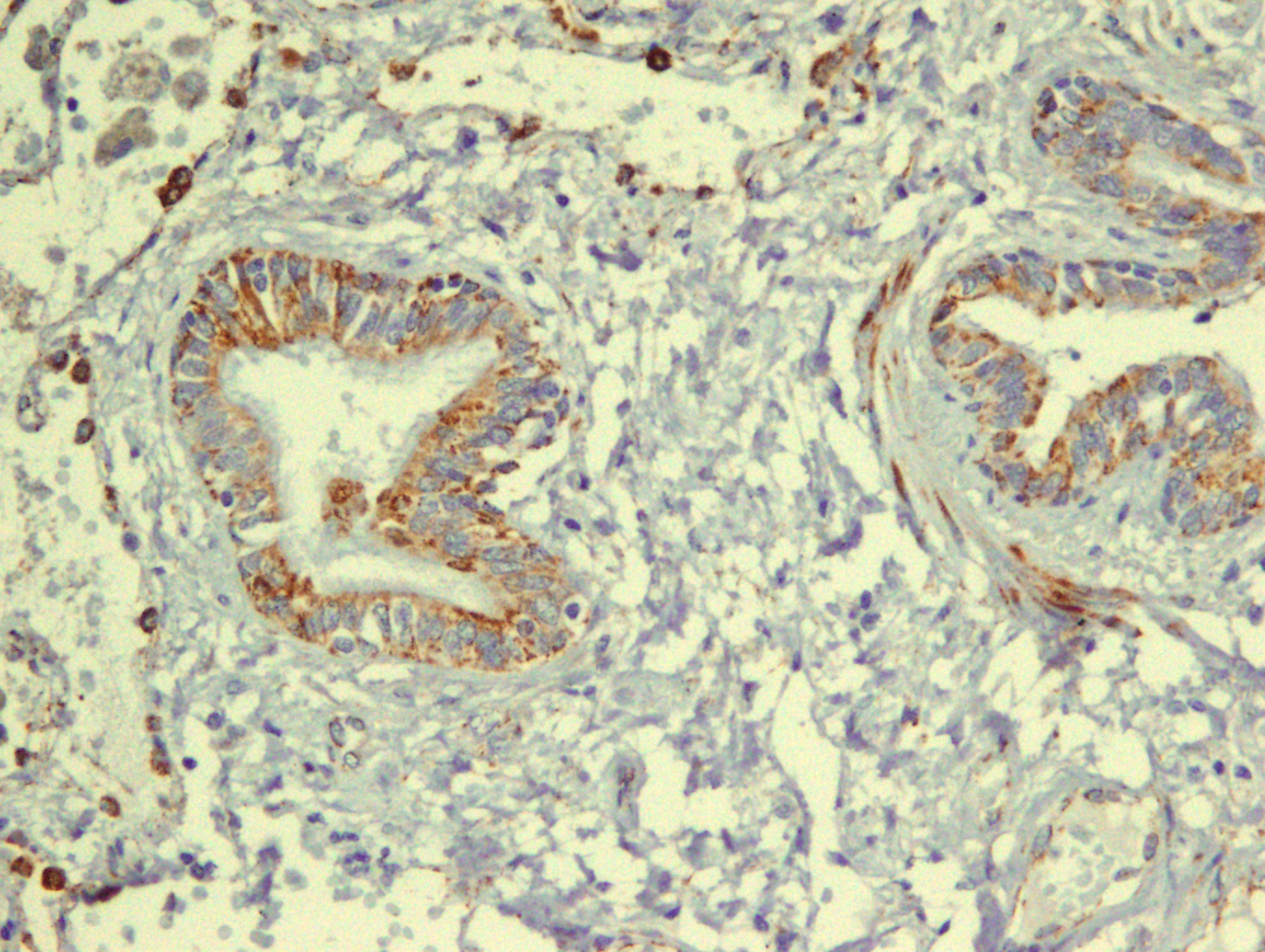

Supplement: Supplementary file 6 — Source Data for Figure 3 [file EMMM-13-e13929-s005.zip › EMM-2021-13929_Fig3/EMM-2021-13929_Fig3A/EMM-2021-13929_Fig3A_Normal-Lung_MAOA_10X.tif]

## Slide 1
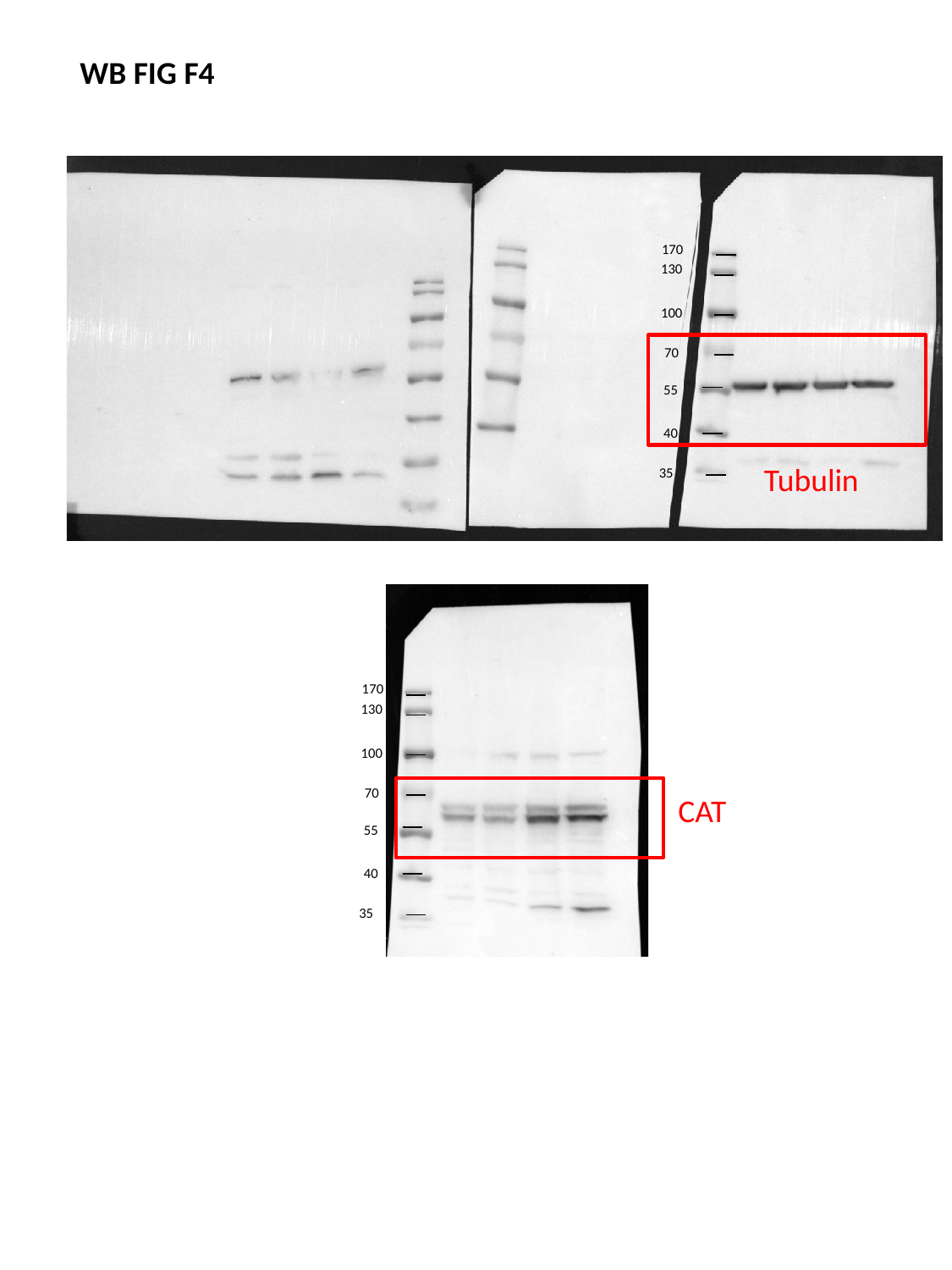

WB FIG F4
170
130
100
70
55
40
35
Tubulin
170
130
100
70
55
40
35
CAT

Supplement: Supplementary file 7 — Source Data for Figure 4 [file EMMM-13-e13929-s004.zip › EMM-2021-13929_Fig4/EMM-2021-13929_Fig4F/EMM-2021-13929_Fig4F_withMARKERS-CAT-Tubulin.pptx]

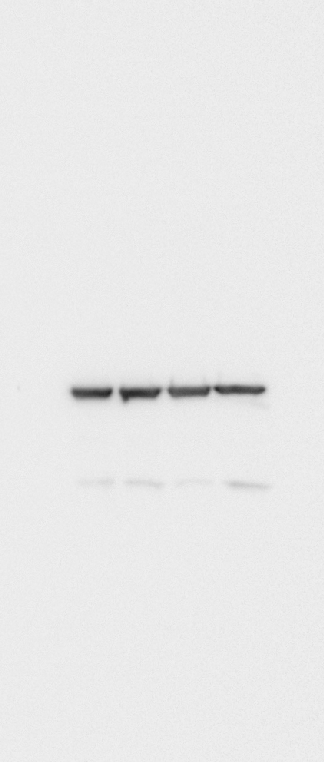

Supplement: Supplementary file 7 — Source Data for Figure 4 [file EMMM-13-e13929-s004.zip › EMM-2021-13929_Fig4/EMM-2021-13929_Fig4F/EMM-2021-13929_Fig4F_TUBULIN.tif]

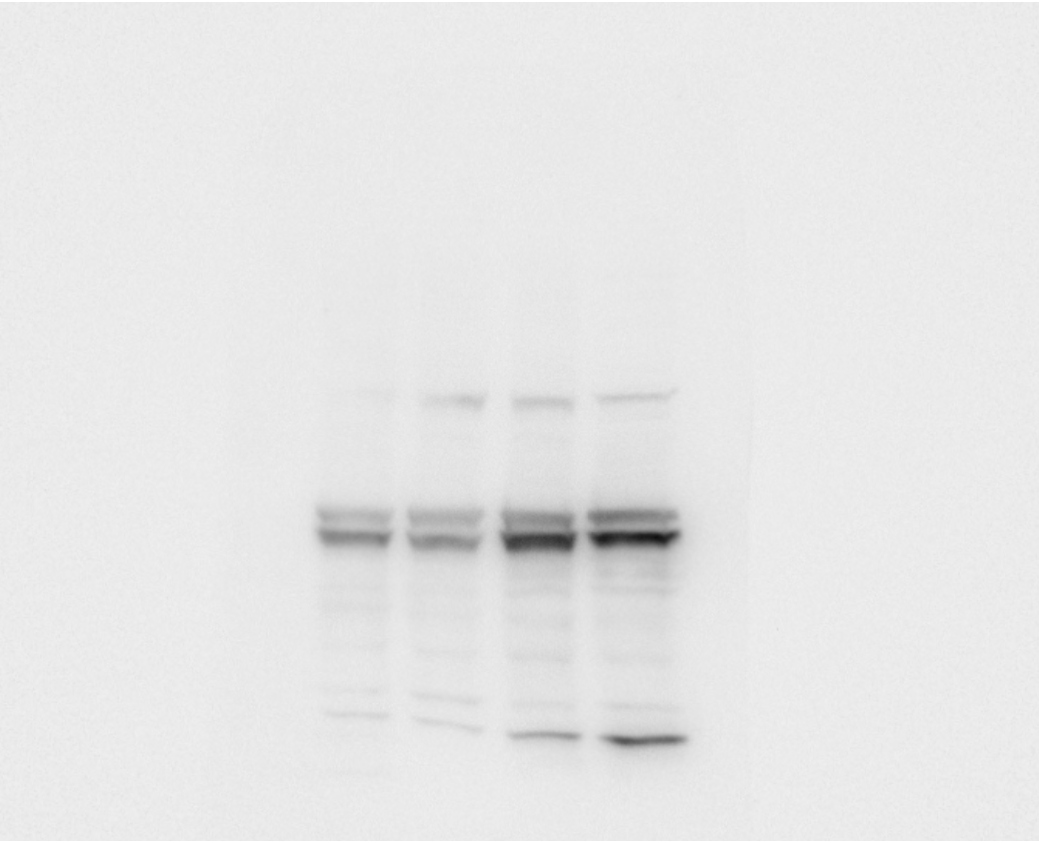

Supplement: Supplementary file 7 — Source Data for Figure 4 [file EMMM-13-e13929-s004.zip › EMM-2021-13929_Fig4/EMM-2021-13929_Fig4F/EMM-2021-13929_Fig4F_CAT.tif]

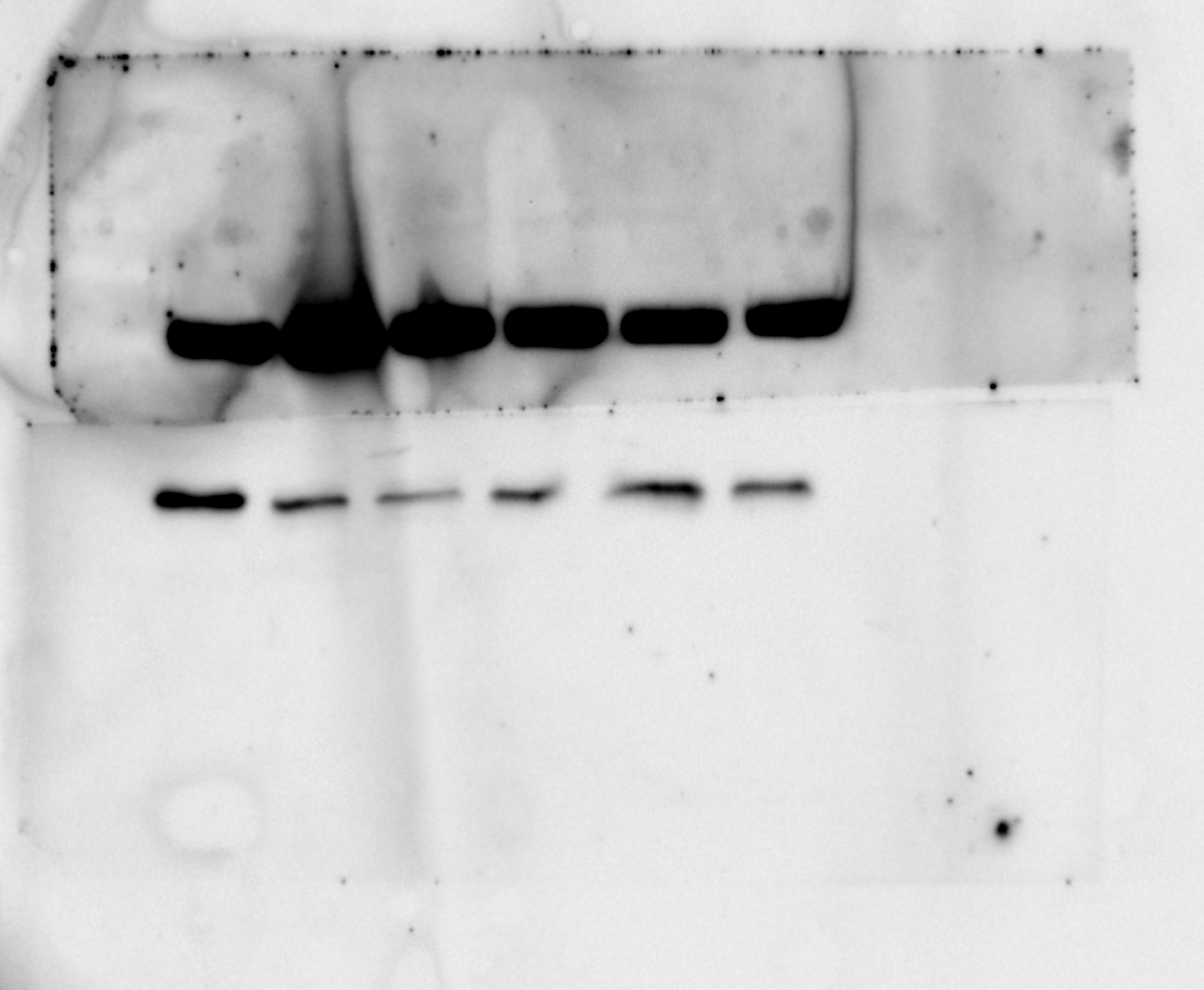

Supplement: Supplementary file 7 — Source Data for Figure 4 [file EMMM-13-e13929-s004.zip › EMM-2021-13929_Fig4/EMM-2021-13929_Fig4A/EMM-2021-13929_Fig4A_VDAC1_bottom_lanes 3-4.tif]

## Slide 1
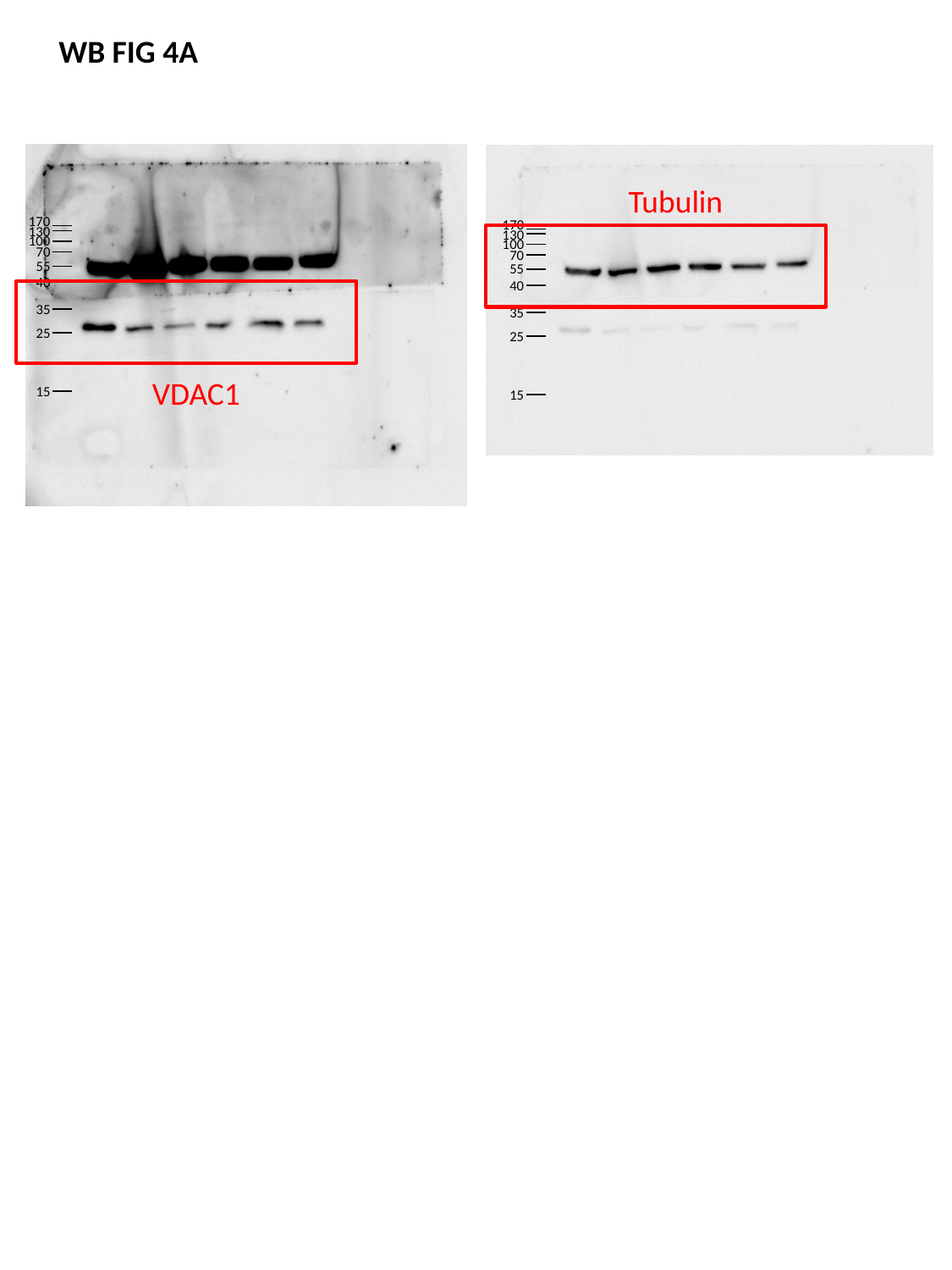

WB FIG 4A
Tubulin
170
130
100
70
55
40
35
25
15
170
130
100
70
55
40
35
25
15
VDAC1

Supplement: Supplementary file 7 — Source Data for Figure 4 [file EMMM-13-e13929-s004.zip › EMM-2021-13929_Fig4/EMM-2021-13929_Fig4A/EMM-2021-13929_Fig4A_withMARKERS_Tubulin_VDAC1.pptx]

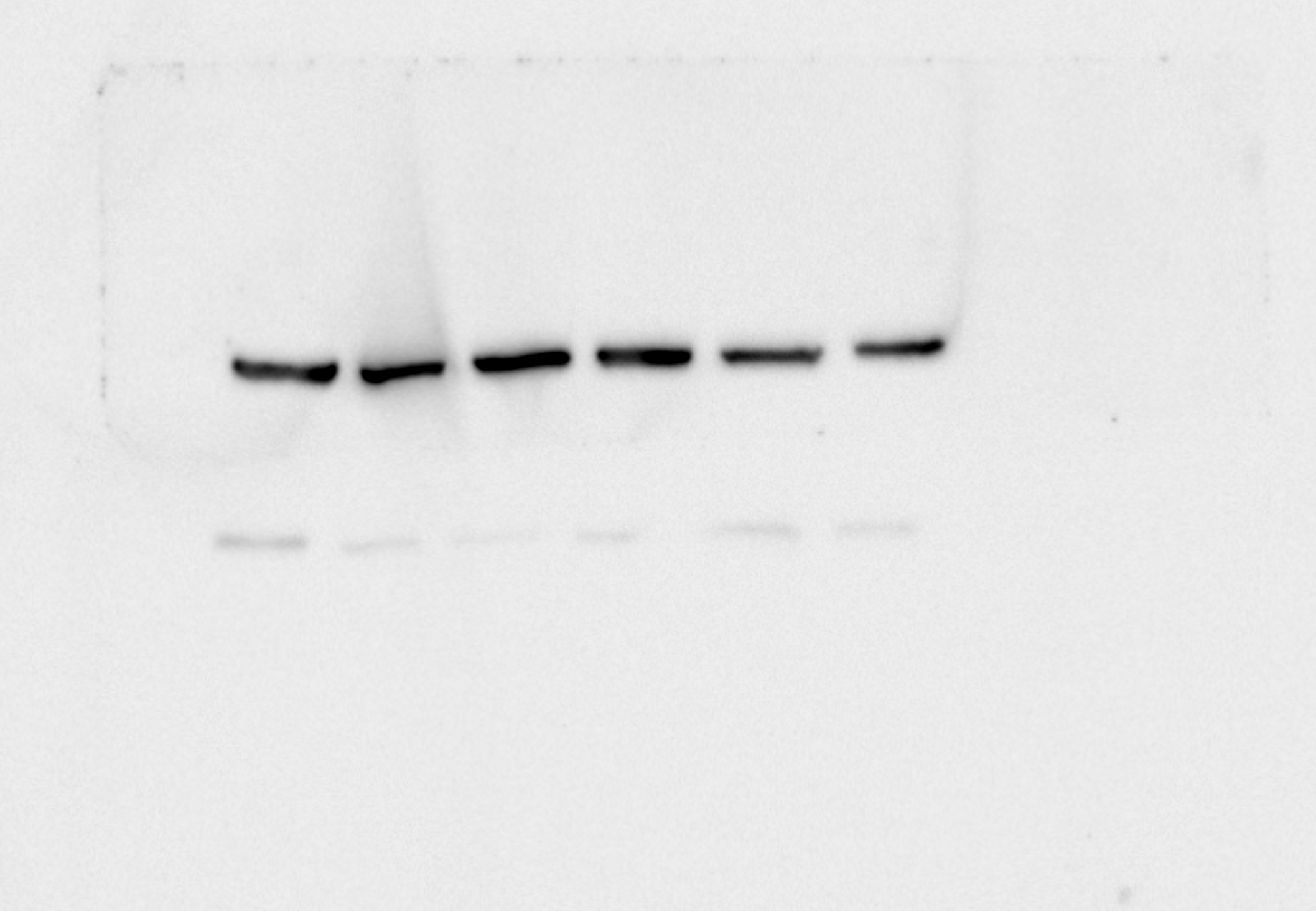

Supplement: Supplementary file 7 — Source Data for Figure 4 [file EMMM-13-e13929-s004.zip › EMM-2021-13929_Fig4/EMM-2021-13929_Fig4A/EMM-2021-13929_Fig4A_Tubulin_upper_lanes 3-4.tif]

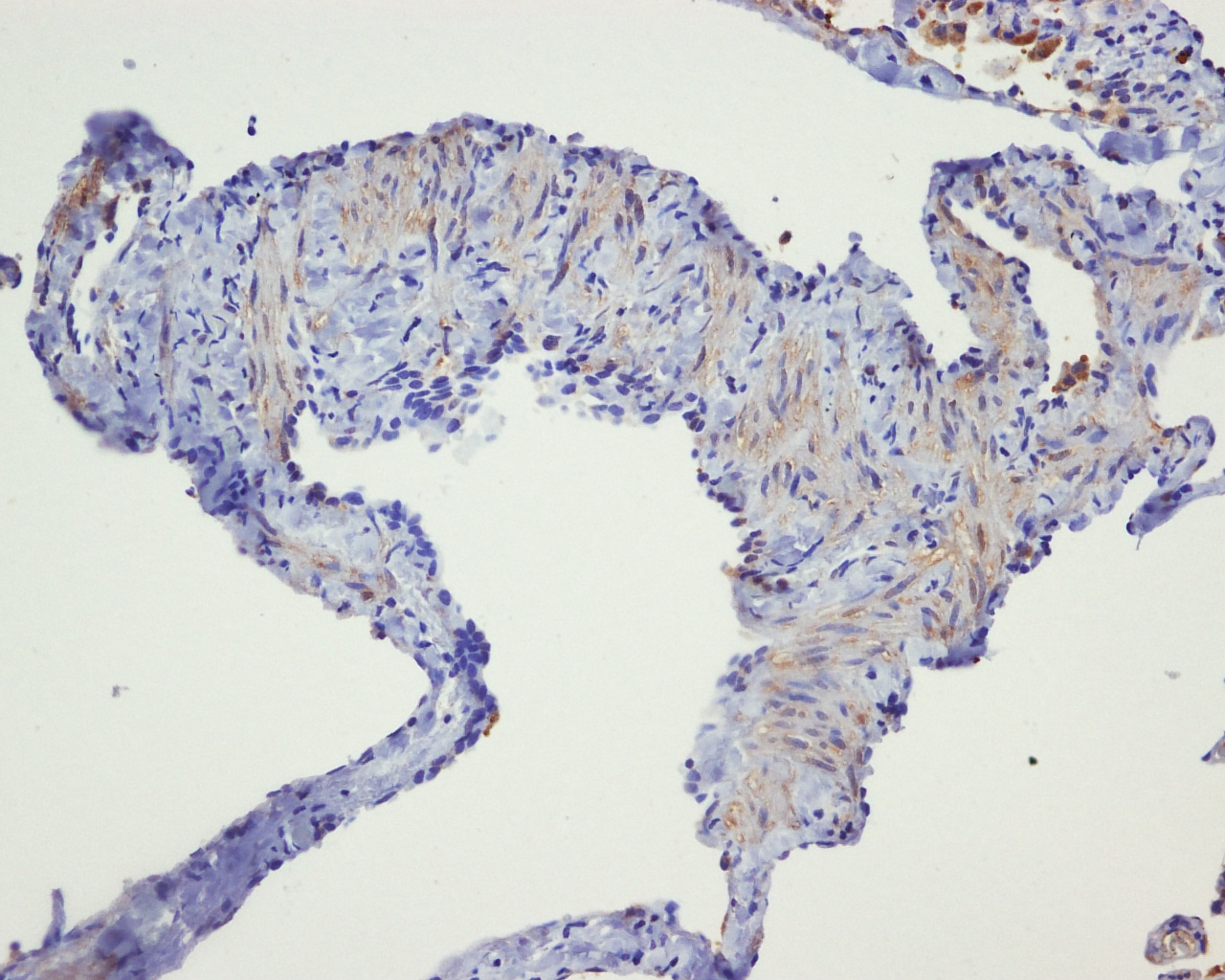

Supplement: Supplementary file 7 — Source Data for Figure 4 [file EMMM-13-e13929-s004.zip › EMM-2021-13929_Fig4/EMM-2021-13929_Fig4G/EMM-2021-13929_Fig4G_ACROLEIN_20X_2.tif]

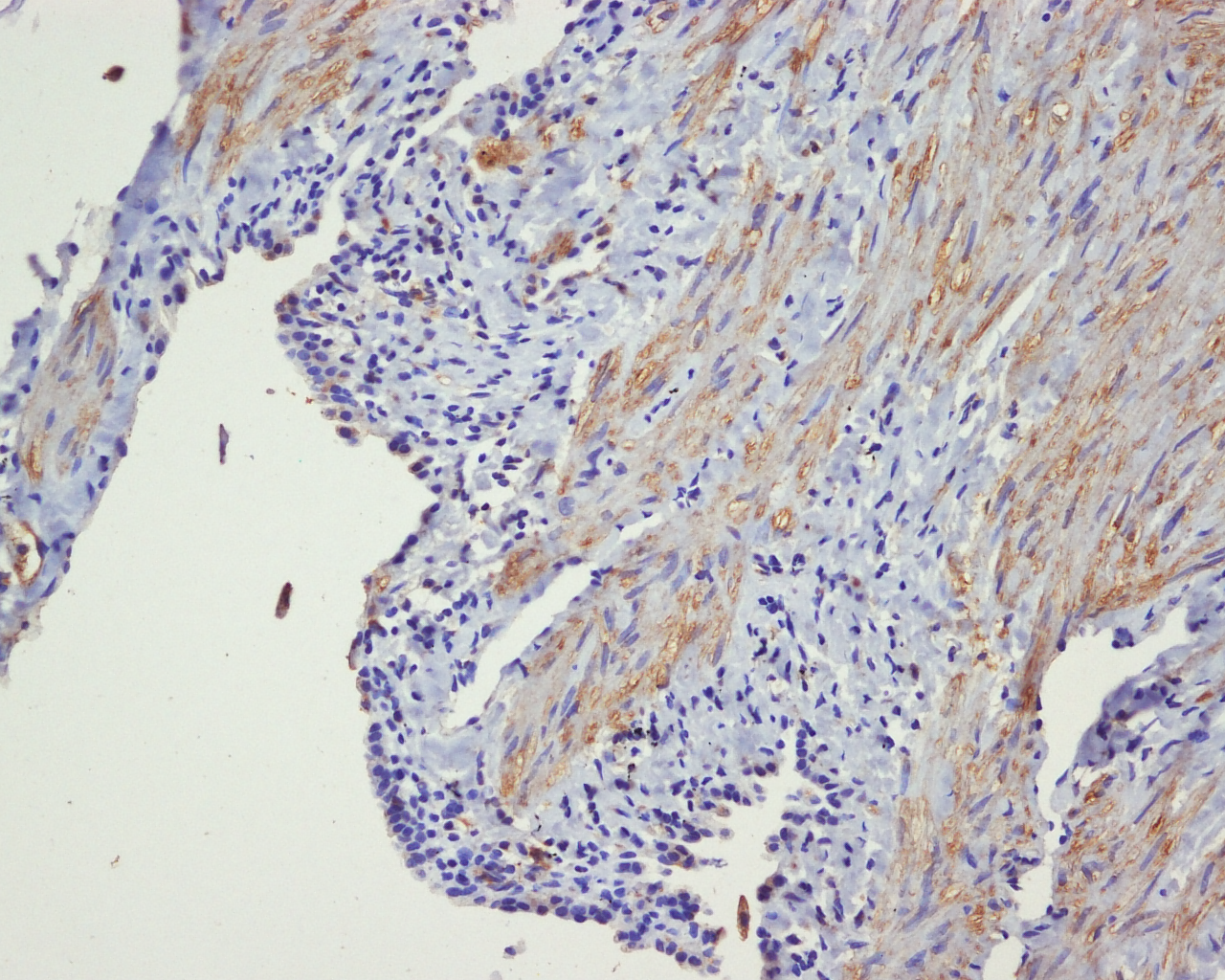

Supplement: Supplementary file 7 — Source Data for Figure 4 [file EMMM-13-e13929-s004.zip › EMM-2021-13929_Fig4/EMM-2021-13929_Fig4G/EMM-2021-13929_Fig4G_ACROLEIN_20X_1.tif]

## Slide 1
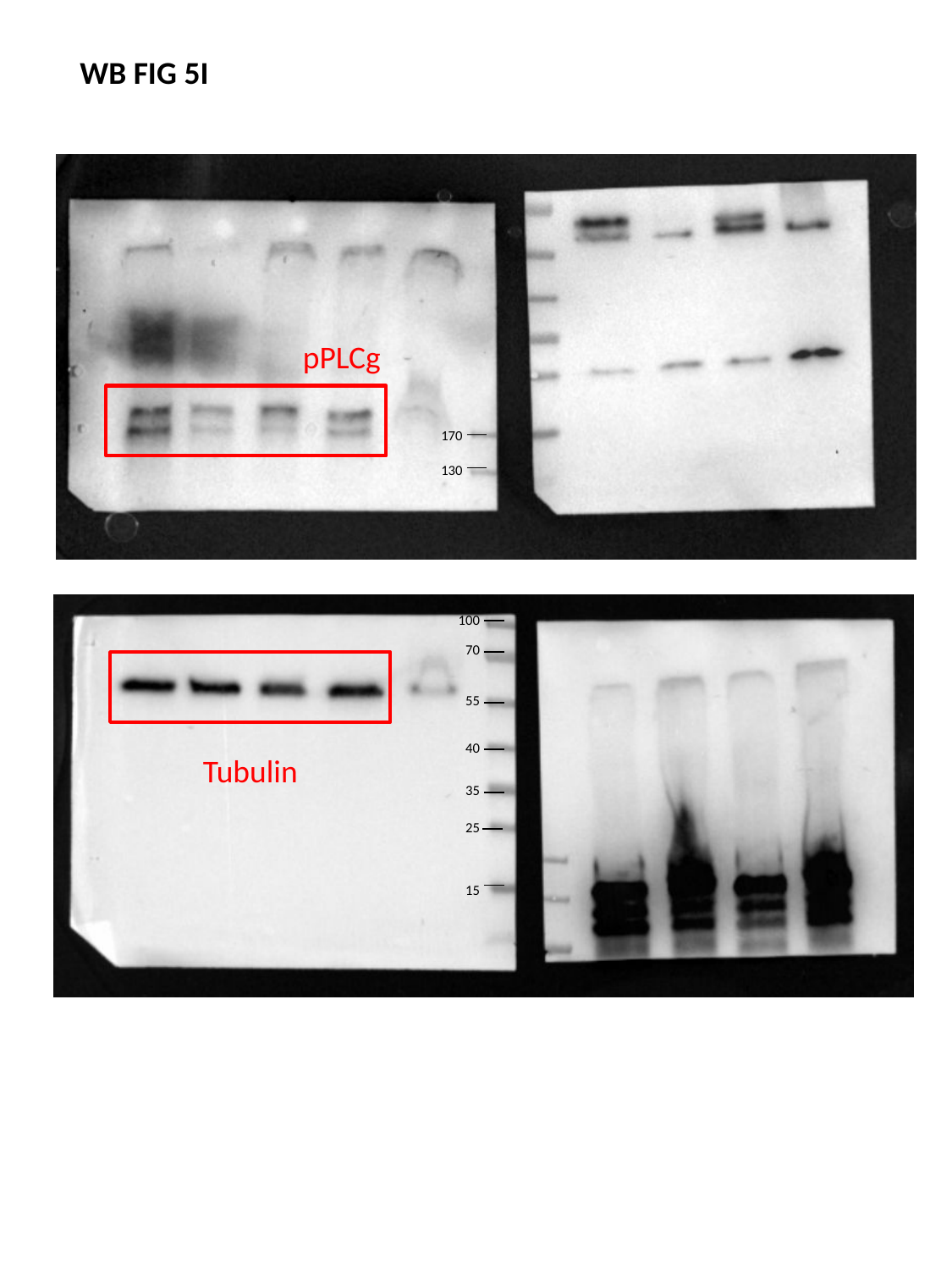

WB FIG 5I
pPLCg
170
130
100
70
55
40
35
25
15
Tubulin

Supplement: Supplementary file 8 — Source Data for Figure 5 [file EMMM-13-e13929-s006.zip › EMM-2021-13929_Fig5/EMM-2021-13929_Fig5I/EMM-2021-13929_Fig5I_withMARKERS_pPLCgamma-Tubulin.pptx]

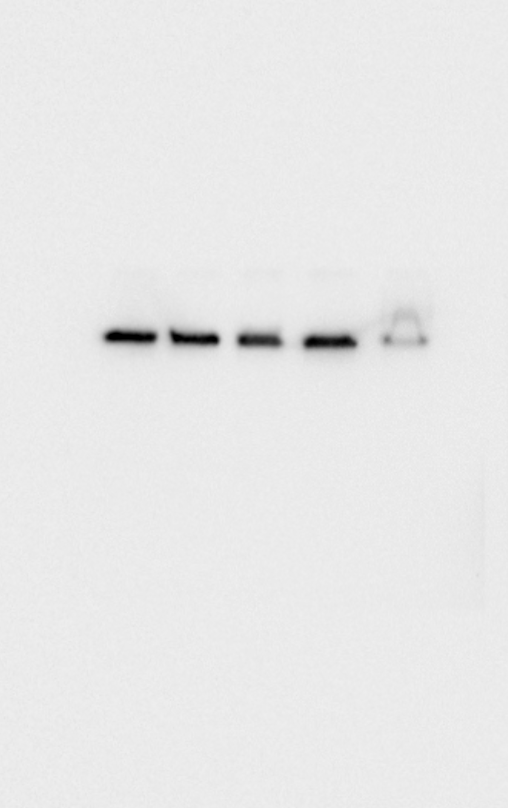

Supplement: Supplementary file 8 — Source Data for Figure 5 [file EMMM-13-e13929-s006.zip › EMM-2021-13929_Fig5/EMM-2021-13929_Fig5I/EMM-2021-13929_Fig5I_TUBULIN.tif]

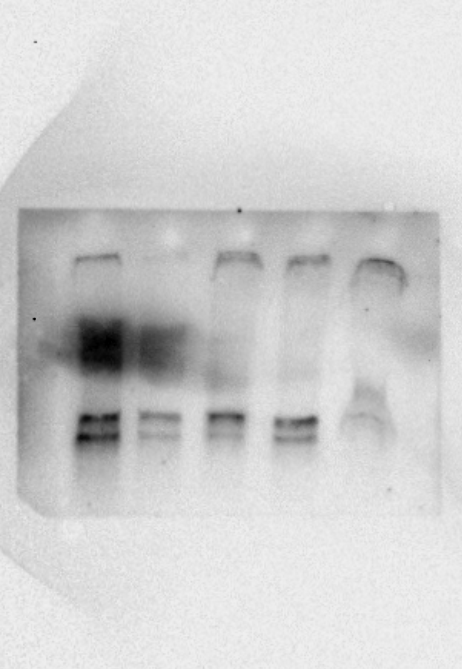

Supplement: Supplementary file 8 — Source Data for Figure 5 [file EMMM-13-e13929-s006.zip › EMM-2021-13929_Fig5/EMM-2021-13929_Fig5I/EMM-2021-13929_Fig5I_pPLCgamma.tif]

## Slide 1
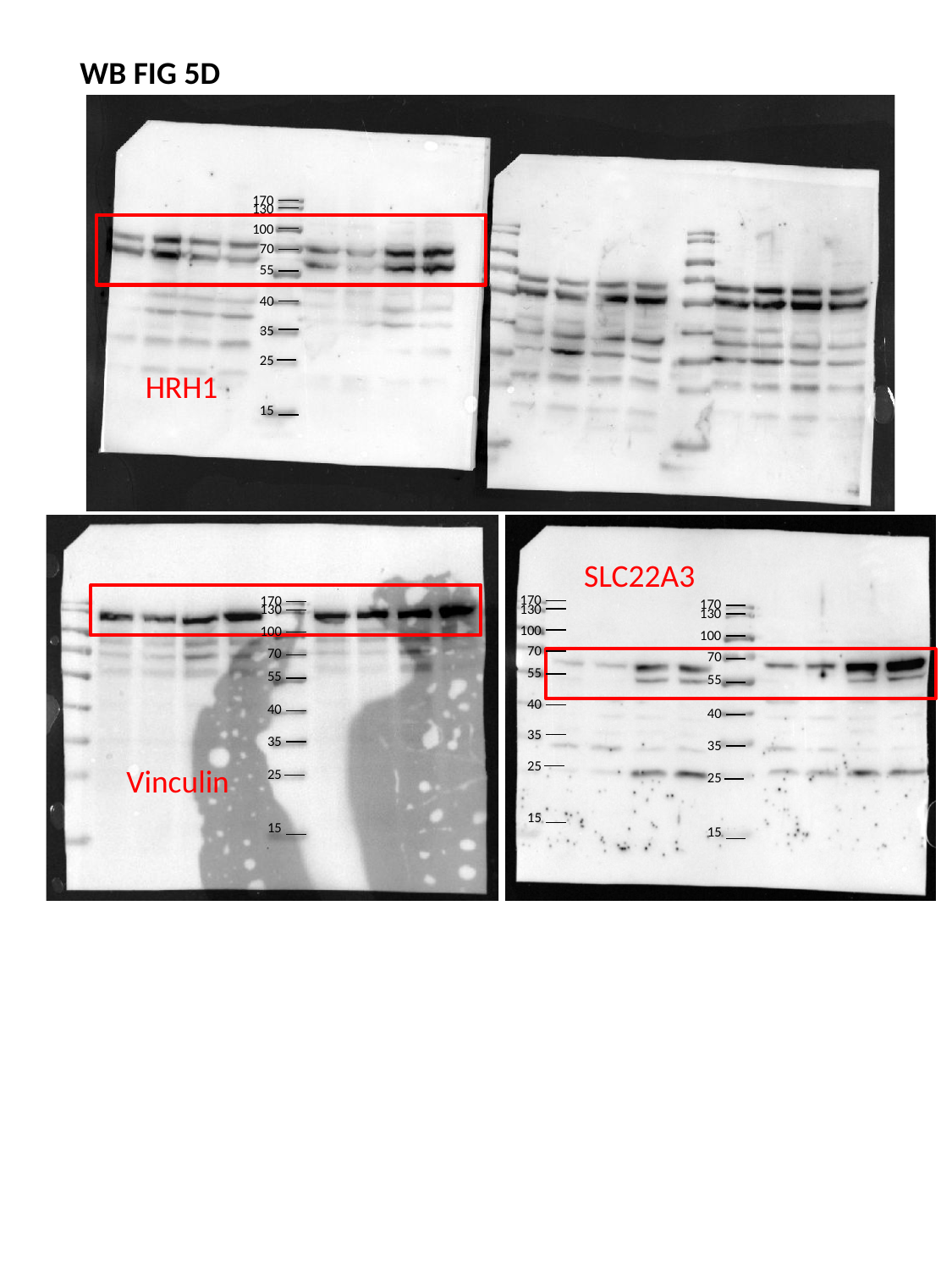

WB FIG 5D
170
130
100
70
55
40
35
25
15
HRH1
SLC22A3
170
130
100
70
55
40
35
25
15
170
130
100
70
55
40
35
25
15
170
130
100
70
55
40
35
25
15
Vinculin

Supplement: Supplementary file 8 — Source Data for Figure 5 [file EMMM-13-e13929-s006.zip › EMM-2021-13929_Fig5/EMM-2021-13929_Fig5D/EMM-2021-13929_Fig5D_withMARKERS_HRH1-SLC22A3-Vinculin.pptx]

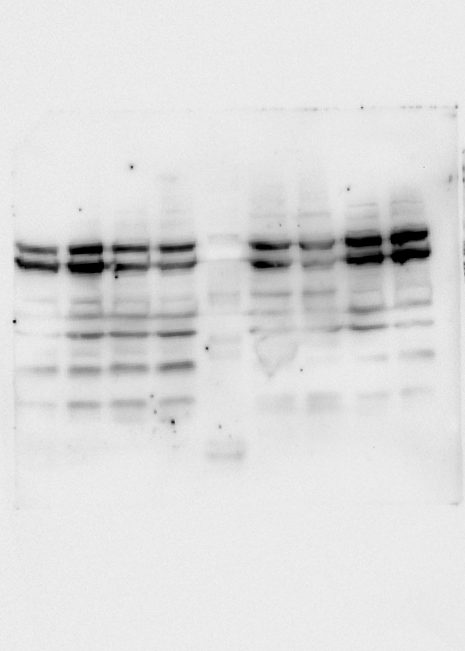

Supplement: Supplementary file 8 — Source Data for Figure 5 [file EMMM-13-e13929-s006.zip › EMM-2021-13929_Fig5/EMM-2021-13929_Fig5D/EMM-2021-13929_Fig5D_HRH1 .tif]

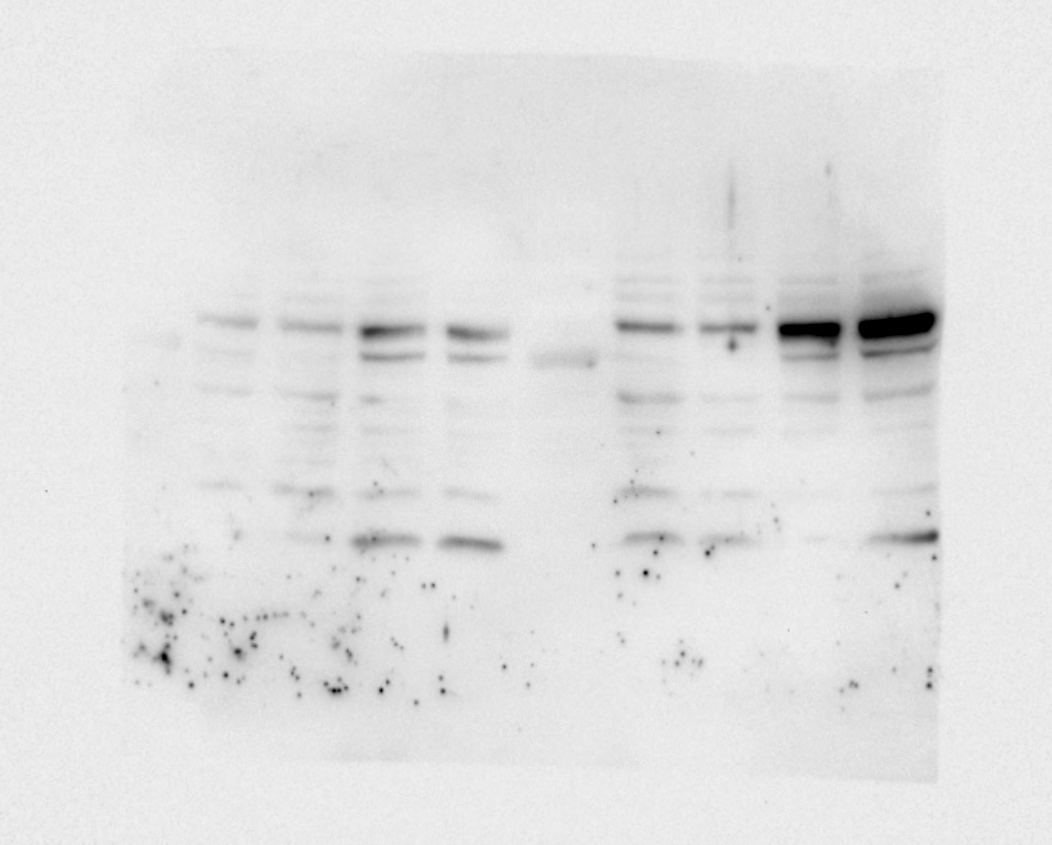

Supplement: Supplementary file 8 — Source Data for Figure 5 [file EMMM-13-e13929-s006.zip › EMM-2021-13929_Fig5/EMM-2021-13929_Fig5D/EMM-2021-13929_Fig5D_SLC22A3.tif]

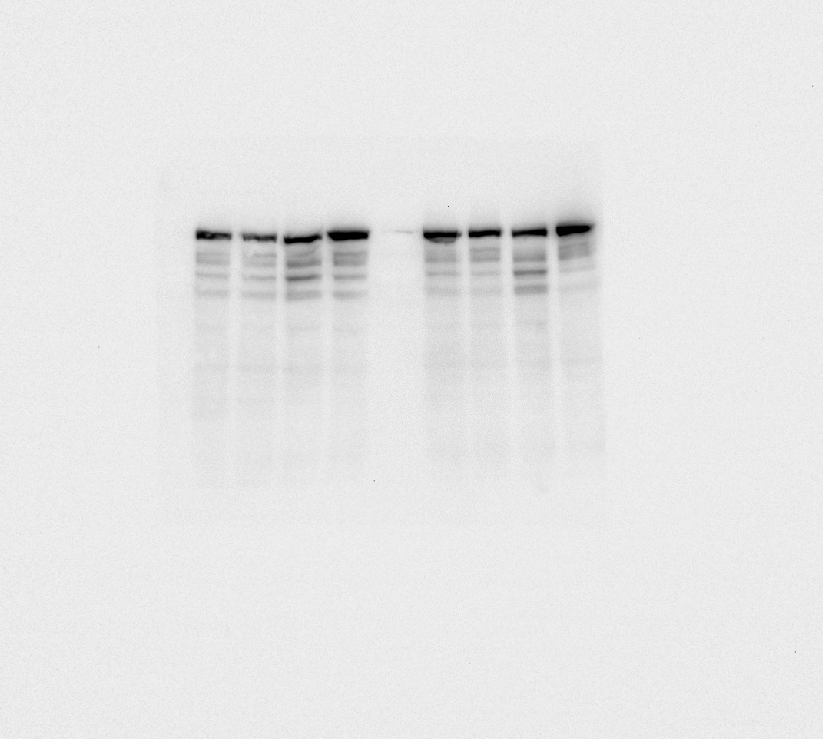

Supplement: Supplementary file 8 — Source Data for Figure 5 [file EMMM-13-e13929-s006.zip › EMM-2021-13929_Fig5/EMM-2021-13929_Fig5D/EMM-2021-13929_Fig5D_Fig5D_VINCULIN.tif]

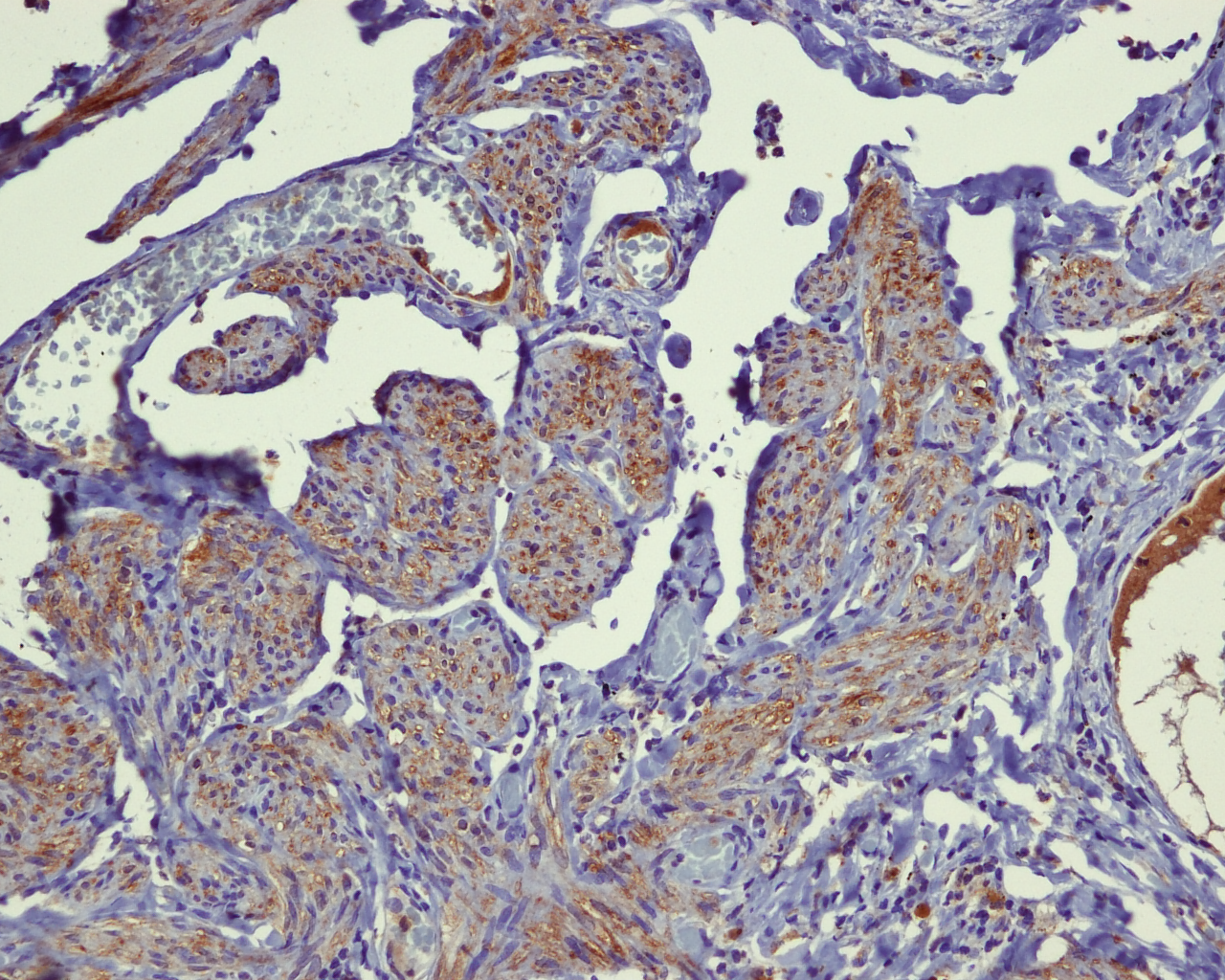

Supplement: Supplementary file 8 — Source Data for Figure 5 [file EMMM-13-e13929-s006.zip › EMM-2021-13929_Fig5/EMM-2021-13929_Fig5E/EMM-2021-13929_Fig5E_HRH1_20X_1.tif]

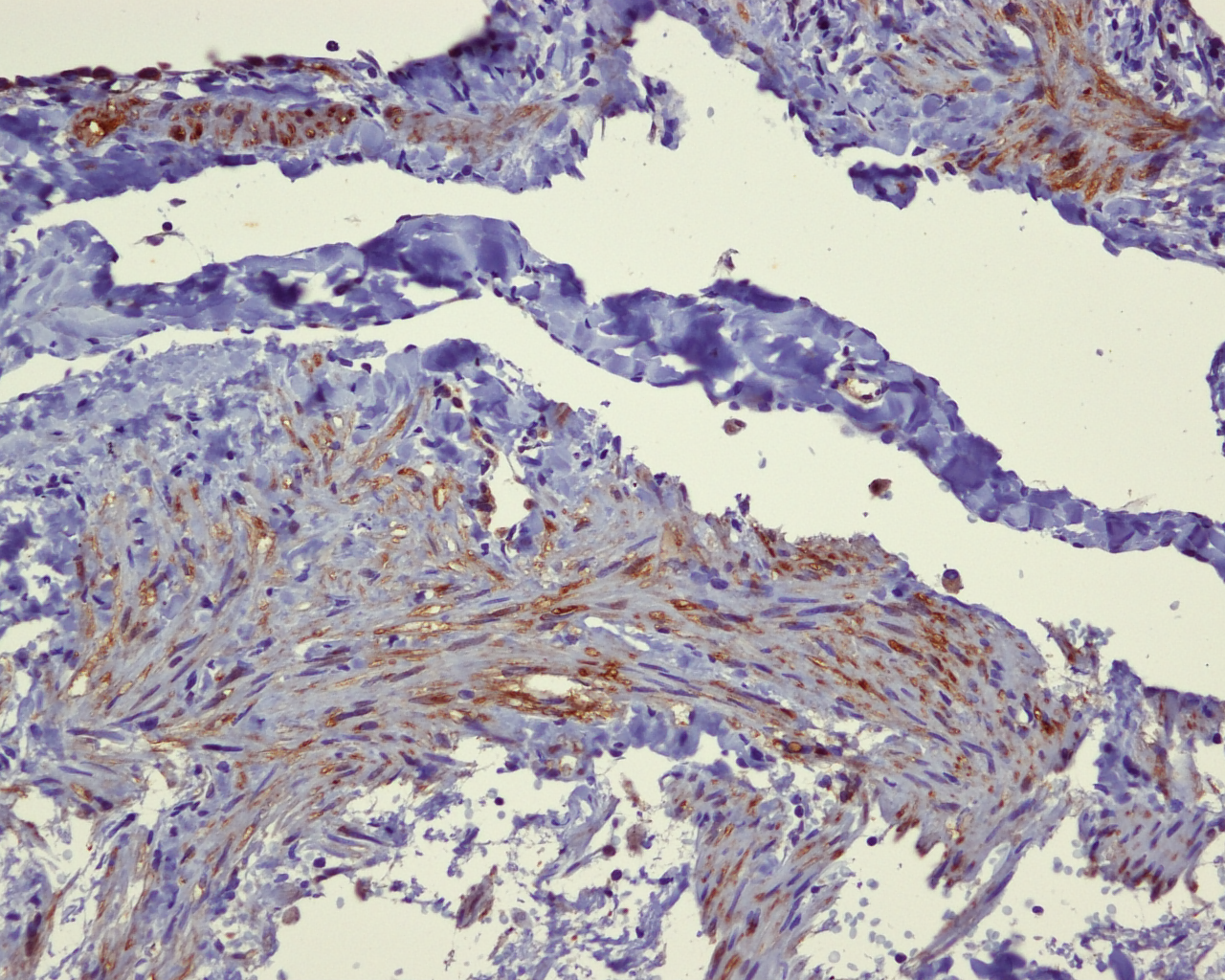

Supplement: Supplementary file 8 — Source Data for Figure 5 [file EMMM-13-e13929-s006.zip › EMM-2021-13929_Fig5/EMM-2021-13929_Fig5E/EMM-2021-13929_Fig5E_HRH1_20X_2.tif]

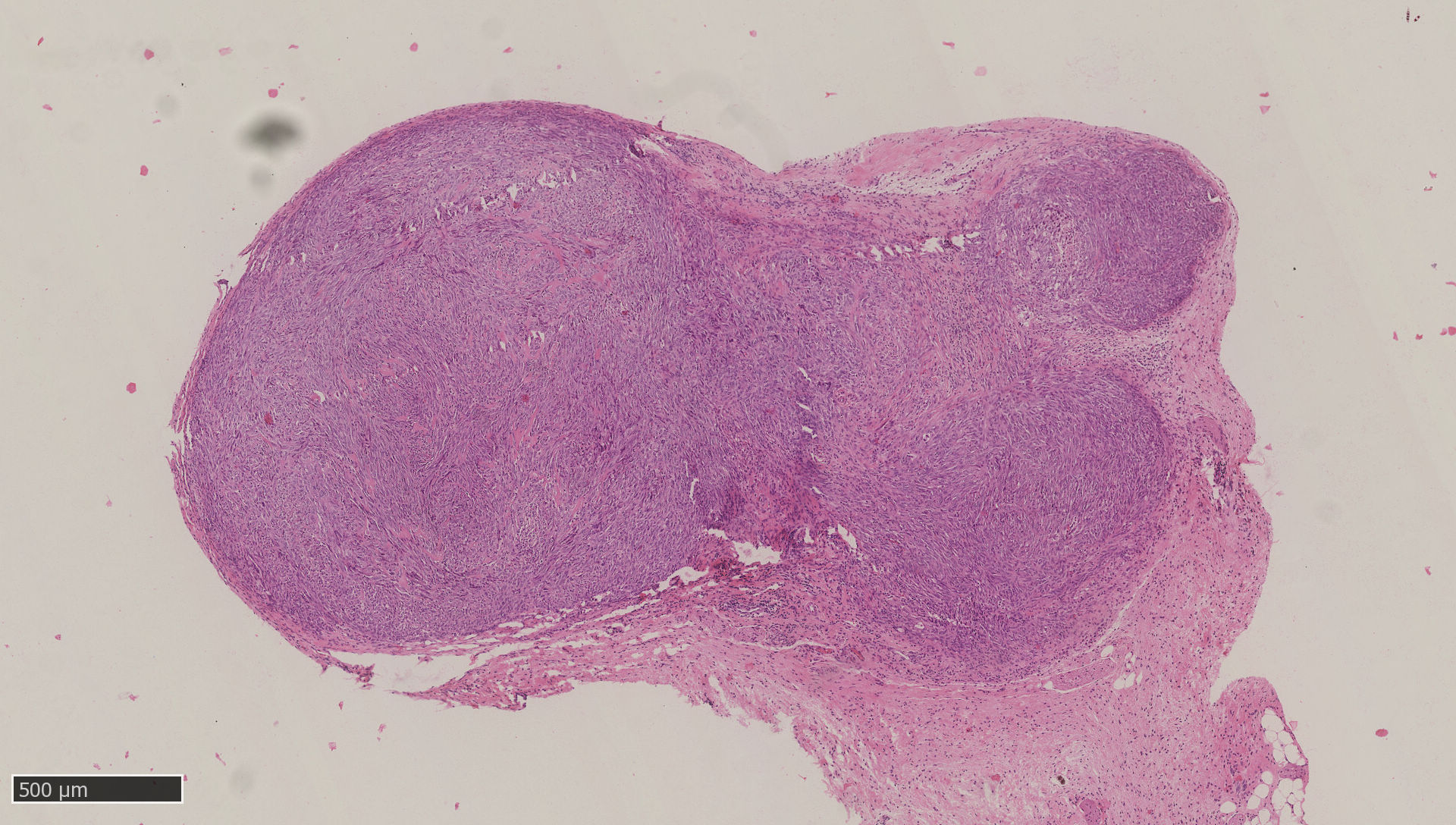

Supplement: Supplementary file 10 — Source Data for Figure 7 [file EMMM-13-e13929-s002.zip › EMM-2021-13929_Fig7/EMM-2021-13929_Fig7E/EMM-2021-13929_Fig7E_Rapamycin_H2R HE 40X.jpg]

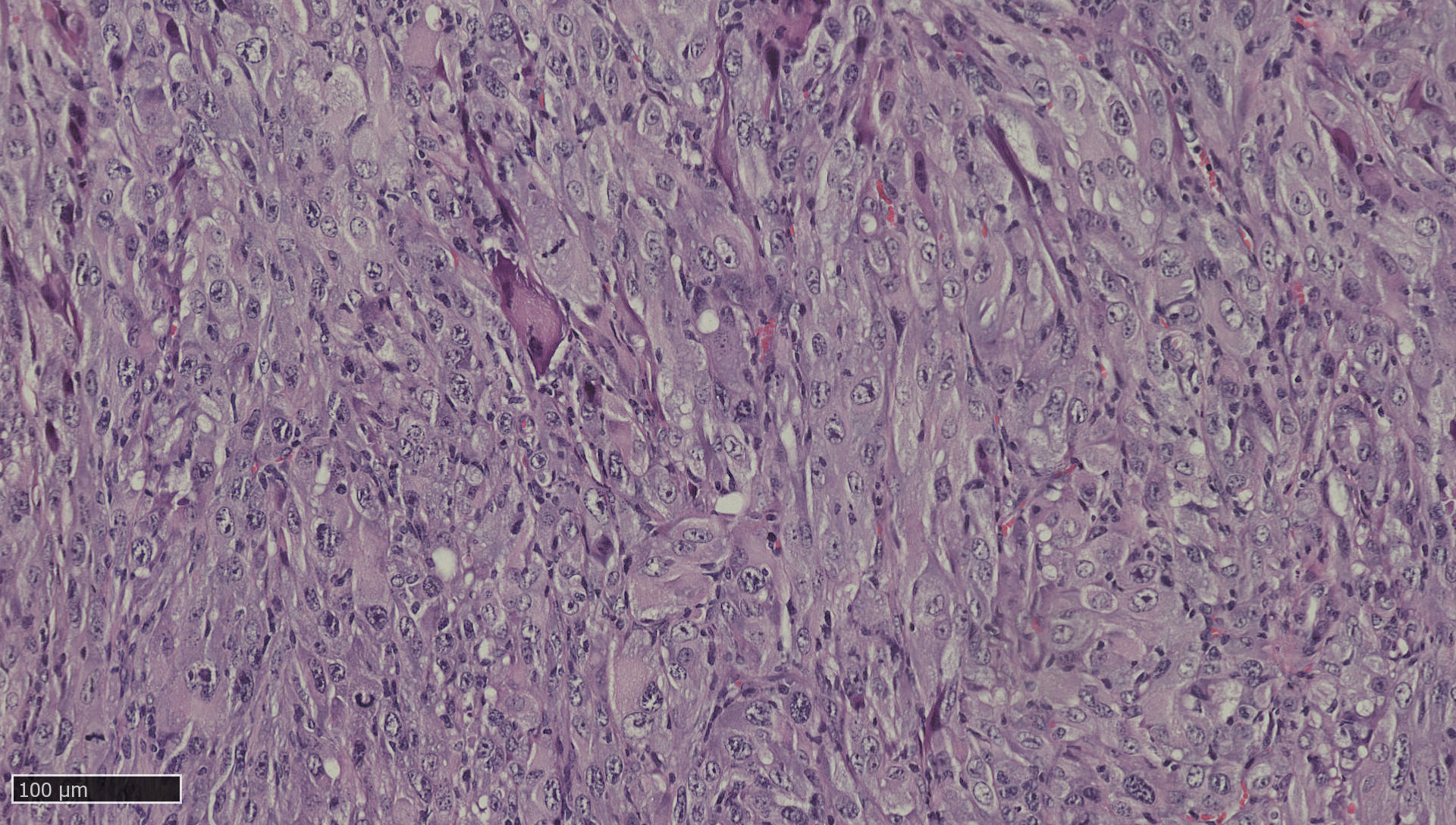

Supplement: Supplementary file 10 — Source Data for Figure 7 [file EMMM-13-e13929-s002.zip › EMM-2021-13929_Fig7/EMM-2021-13929_Fig7E/EMM-2021-13929_Fig7E_Loratadine_H3R HE 200X.jpg]

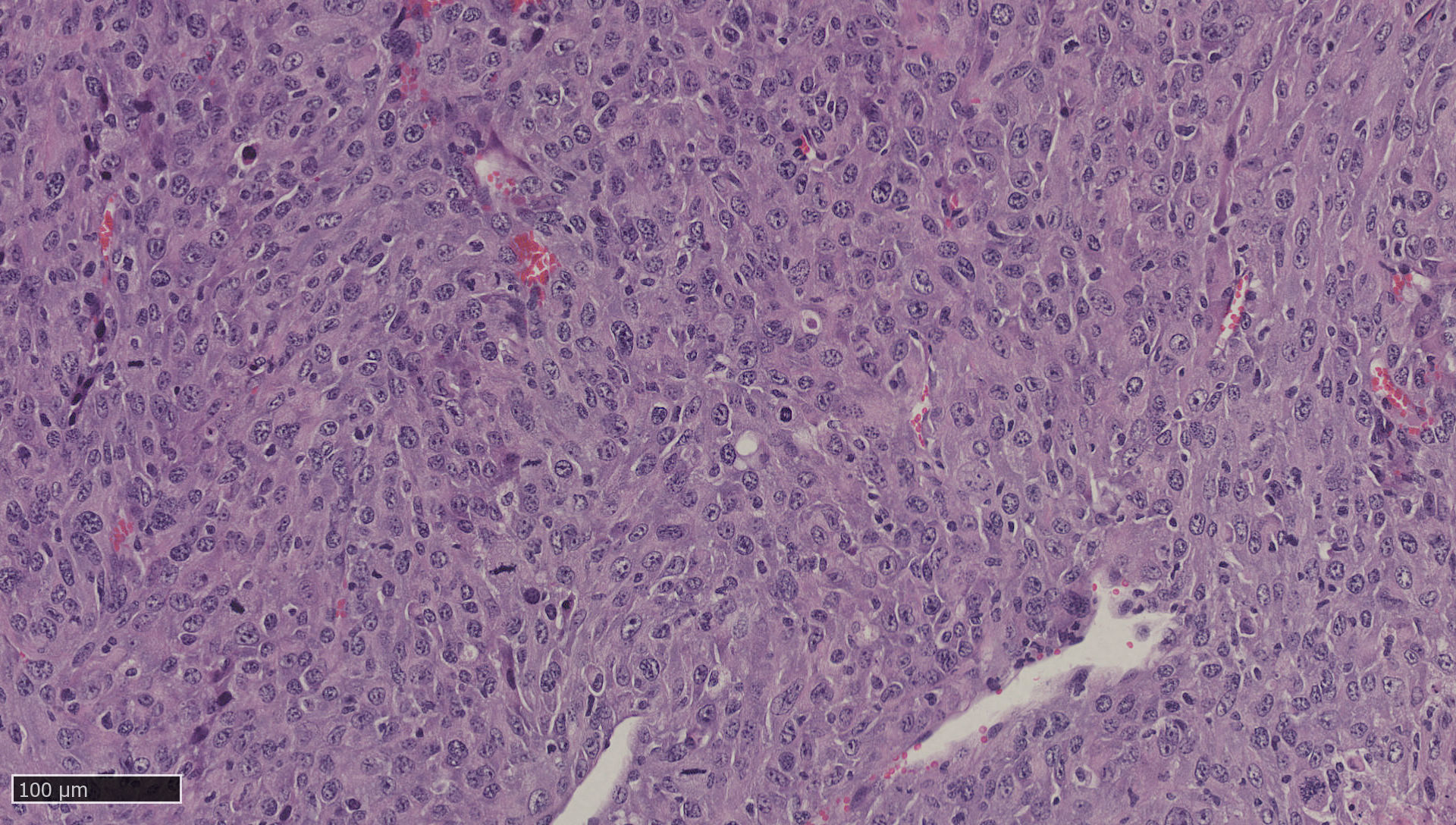

Supplement: Supplementary file 10 — Source Data for Figure 7 [file EMMM-13-e13929-s002.zip › EMM-2021-13929_Fig7/EMM-2021-13929_Fig7E/EMM-2021-13929_Fig7E_Rasagiline_G4R HE 200X.jpg]

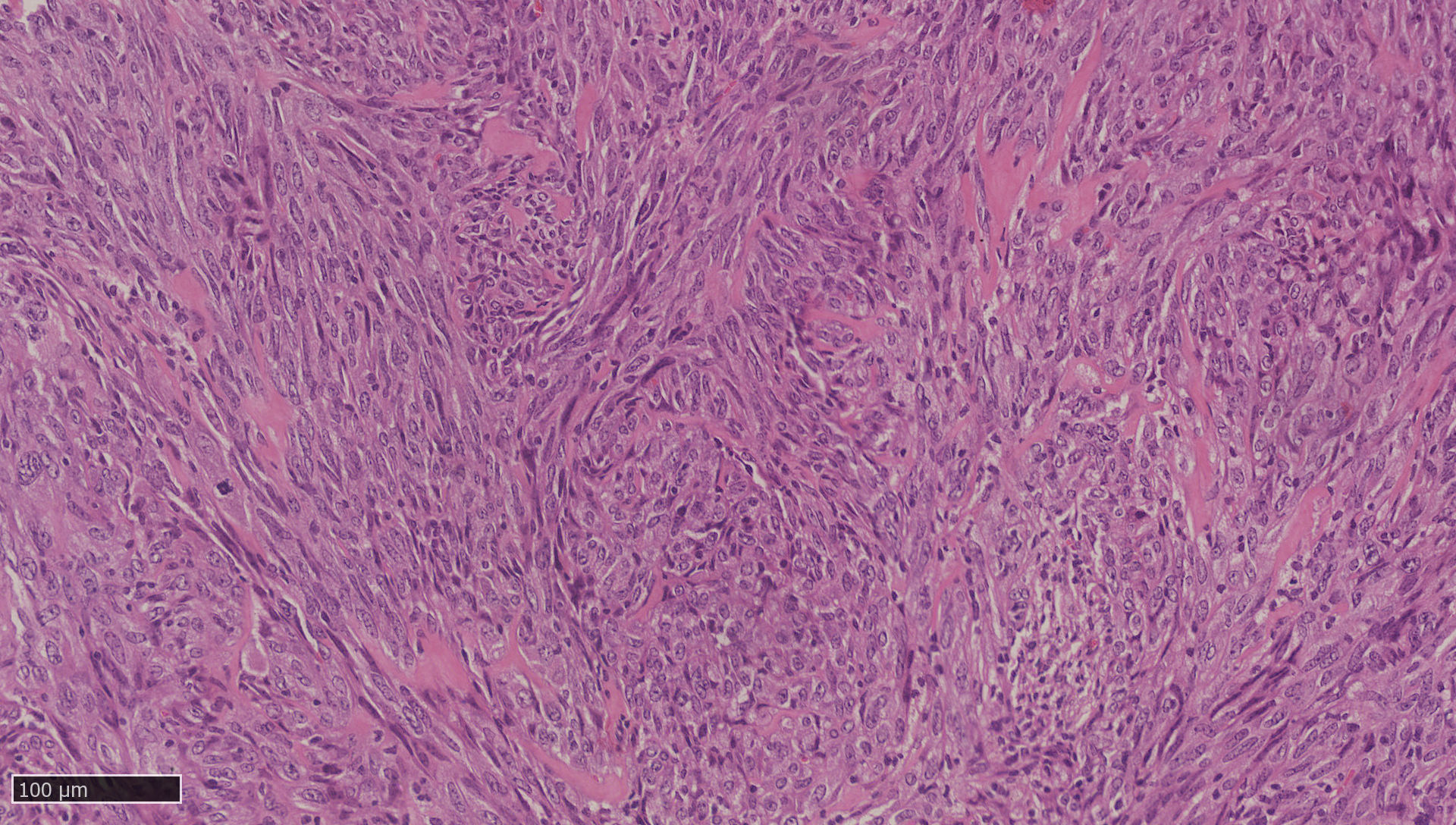

Supplement: Supplementary file 10 — Source Data for Figure 7 [file EMMM-13-e13929-s002.zip › EMM-2021-13929_Fig7/EMM-2021-13929_Fig7E/EMM-2021-13929_Fig7E_Rapamycin_H2R HE 200X.jpg]

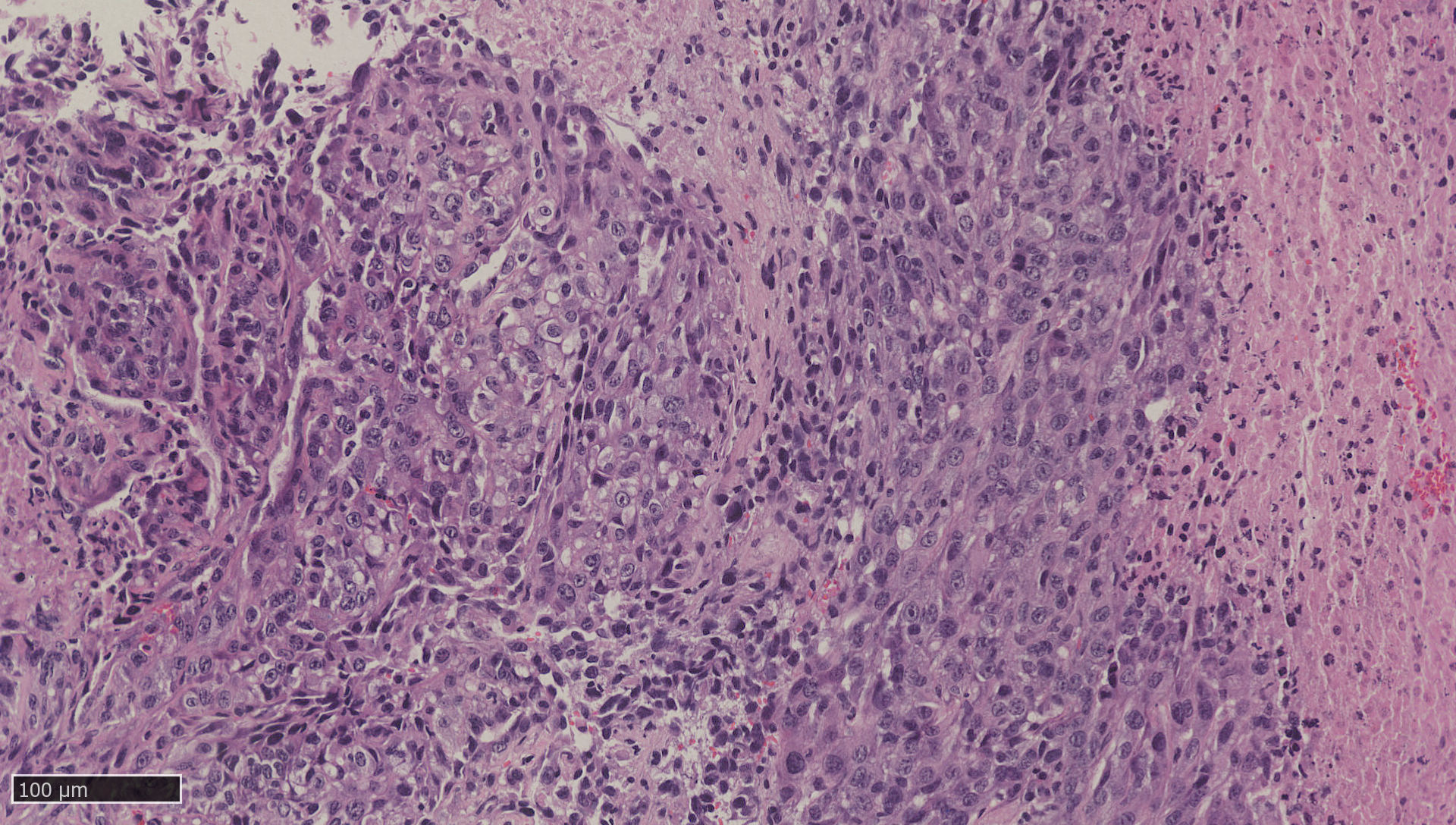

Supplement: Supplementary file 10 — Source Data for Figure 7 [file EMMM-13-e13929-s002.zip › EMM-2021-13929_Fig7/EMM-2021-13929_Fig7E/EMM-2021-13929_Fig7E_Clorgyline_G5R HE 200X.jpg]

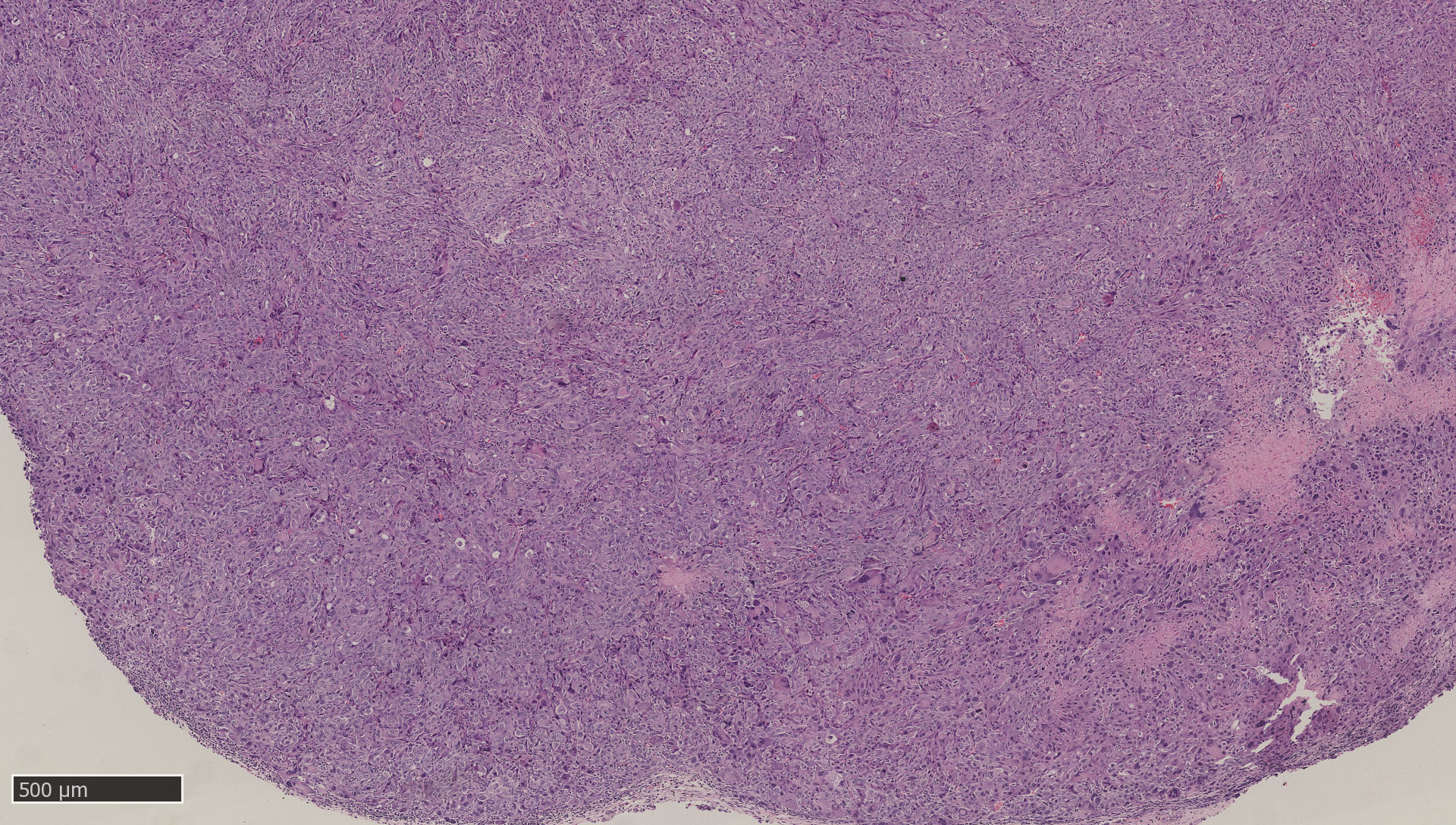

Supplement: Supplementary file 10 — Source Data for Figure 7 [file EMMM-13-e13929-s002.zip › EMM-2021-13929_Fig7/EMM-2021-13929_Fig7E/EMM-2021-13929_Fig7E_Vehicle_H5R HE 40X.jpg]

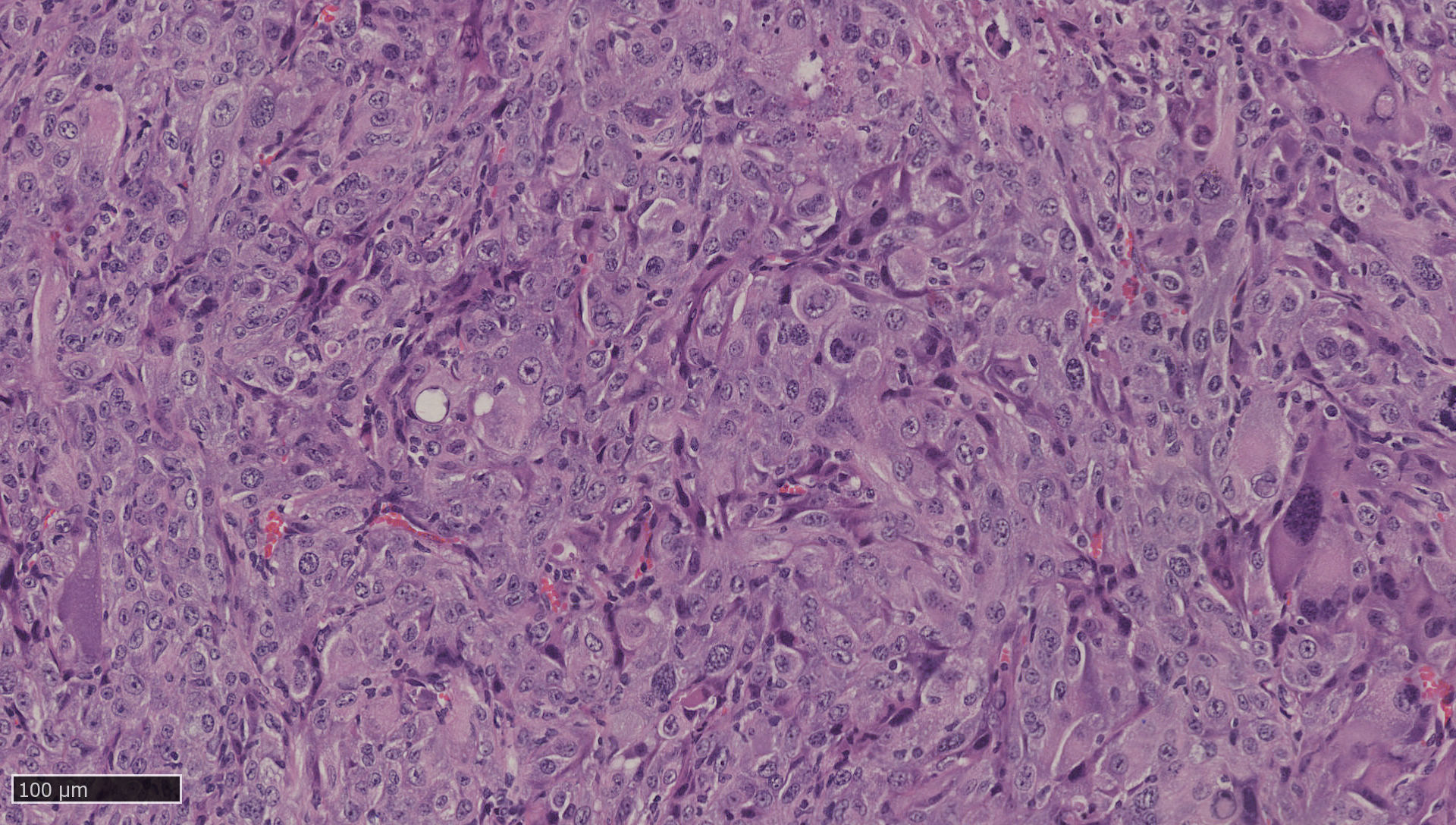

Supplement: Supplementary file 10 — Source Data for Figure 7 [file EMMM-13-e13929-s002.zip › EMM-2021-13929_Fig7/EMM-2021-13929_Fig7E/EMM-2021-13929_Fig7E_Vehicle_H5R HE 200X.jpg]

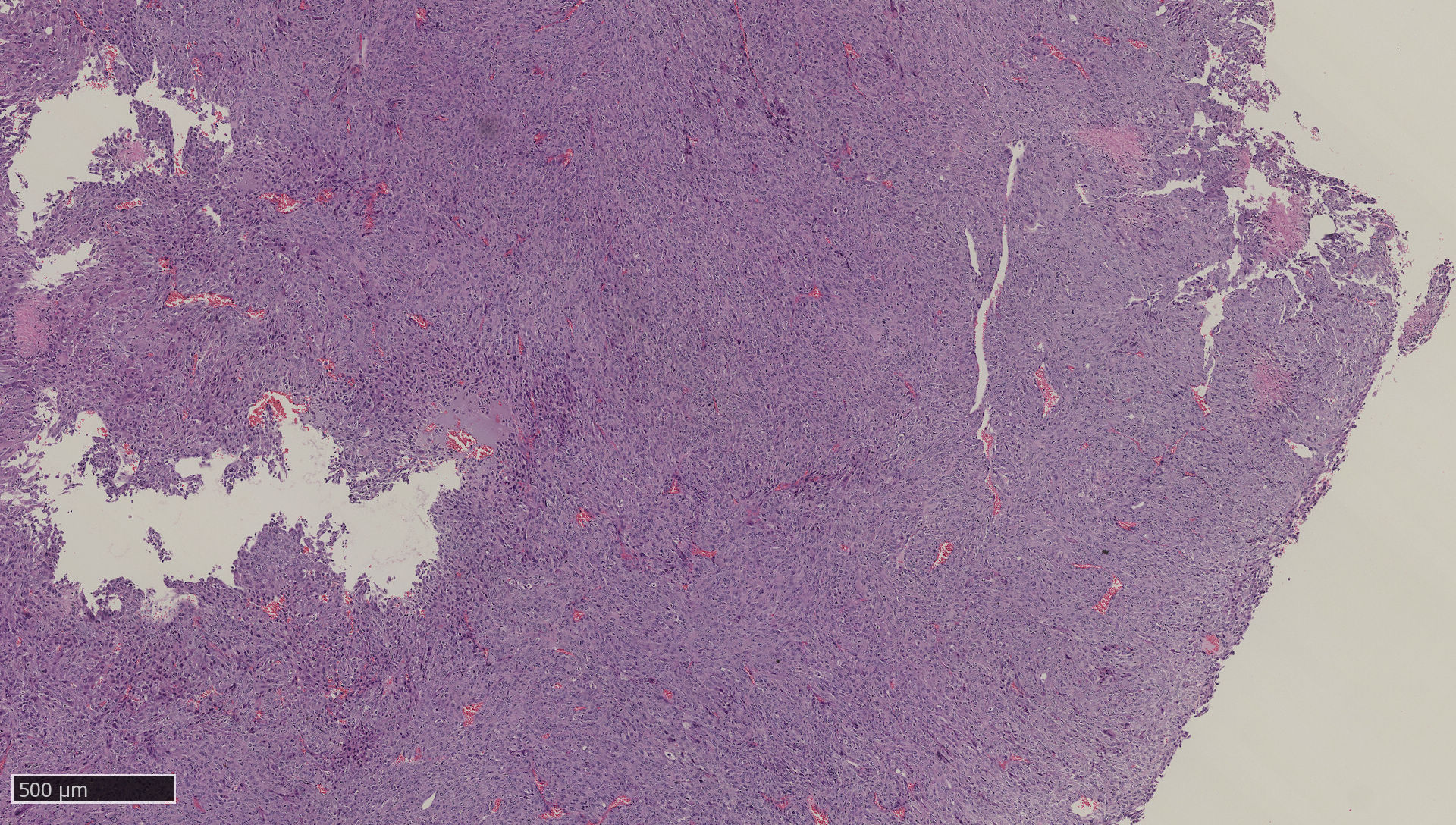

Supplement: Supplementary file 10 — Source Data for Figure 7 [file EMMM-13-e13929-s002.zip › EMM-2021-13929_Fig7/EMM-2021-13929_Fig7E/EMM-2021-13929_Fig7E_Rasagiline_G4R HE 40X.jpg]

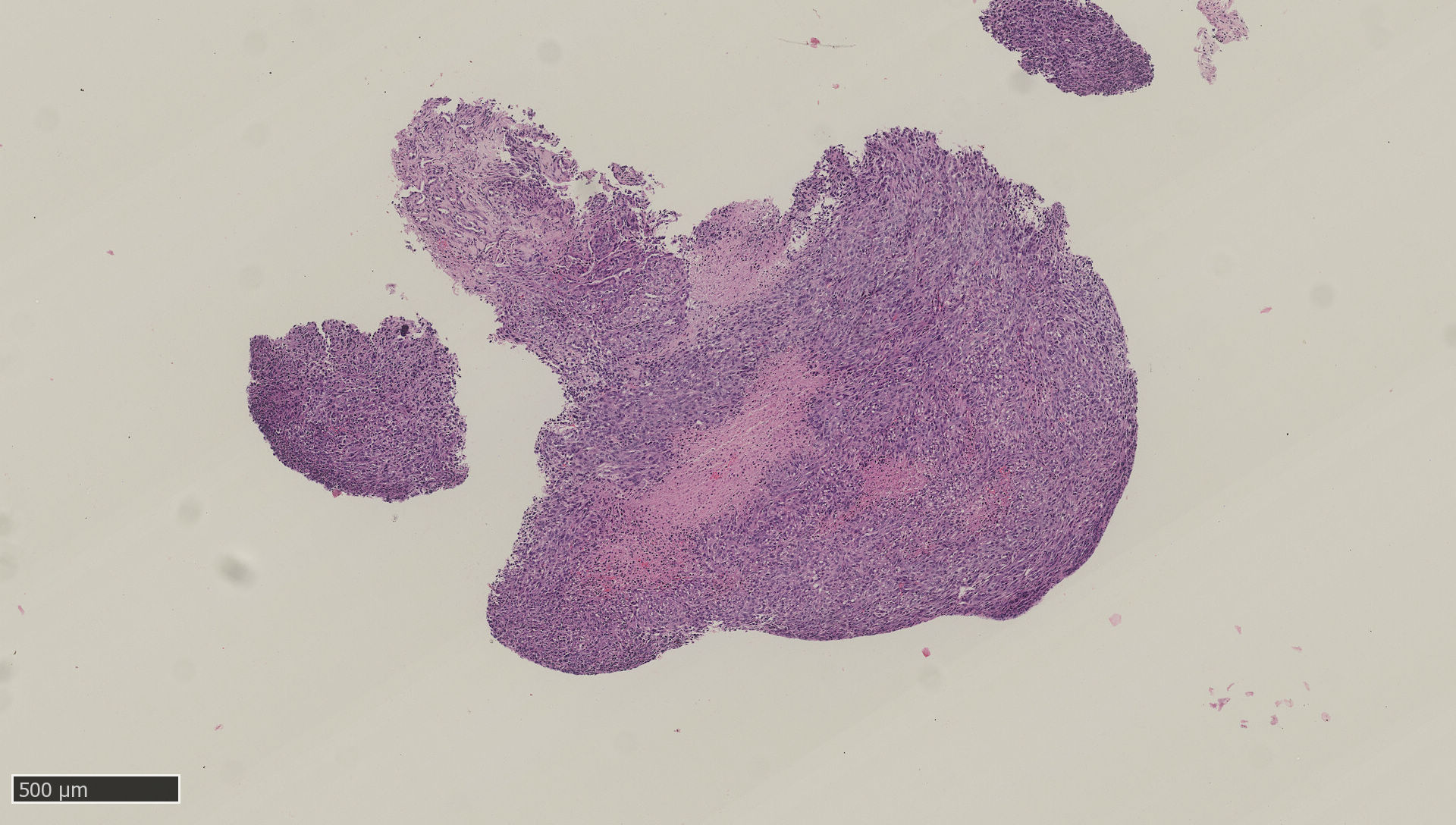

Supplement: Supplementary file 10 — Source Data for Figure 7 [file EMMM-13-e13929-s002.zip › EMM-2021-13929_Fig7/EMM-2021-13929_Fig7E/EMM-2021-13929_Fig7E_Clorgyline_G5R HE 40X.jpg]

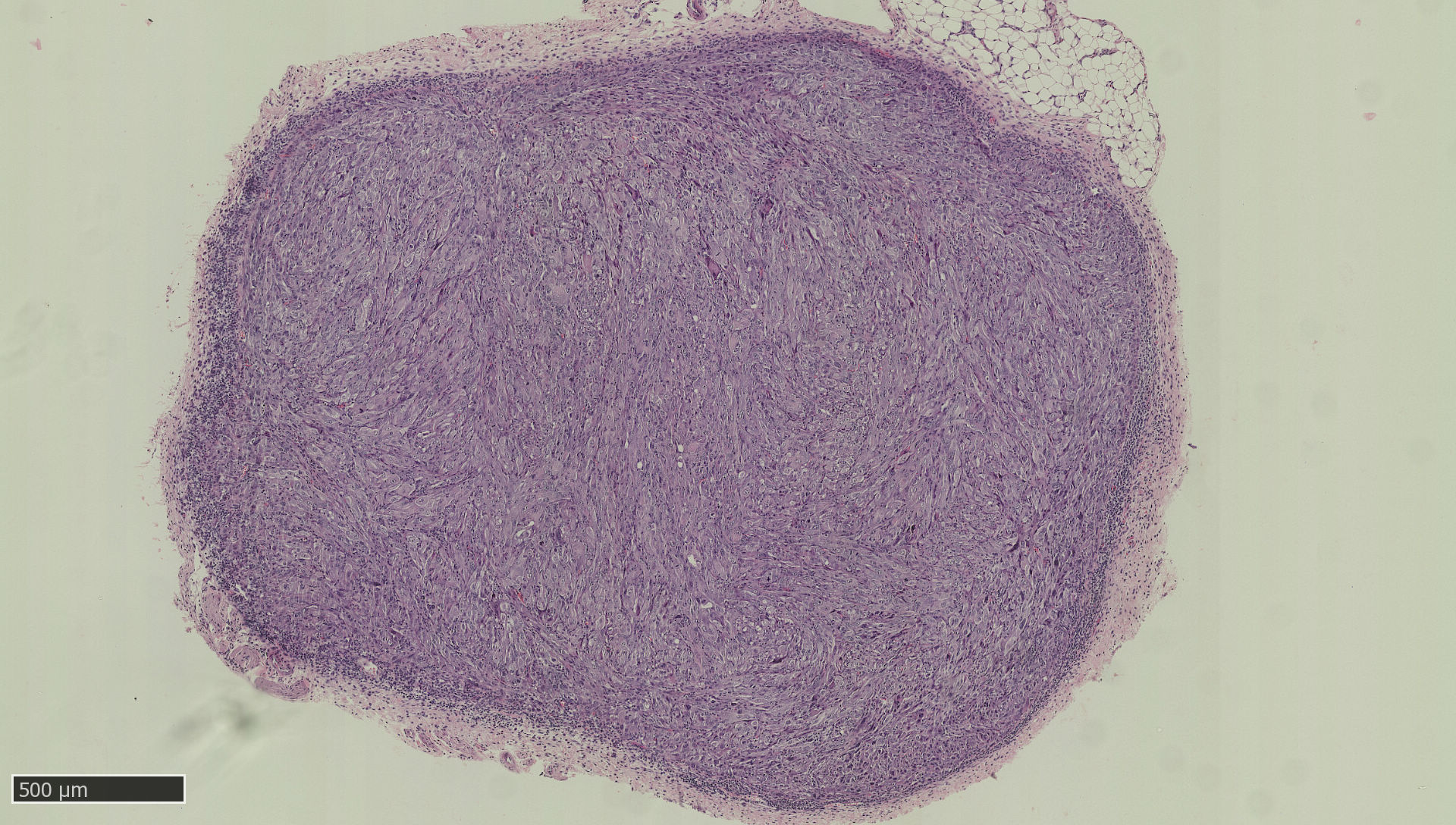

Supplement: Supplementary file 10 — Source Data for Figure 7 [file EMMM-13-e13929-s002.zip › EMM-2021-13929_Fig7/EMM-2021-13929_Fig7E/EMM-2021-13929_Fig7E_Loratadine_H3R HE 40X.jpg]

## Slide 1
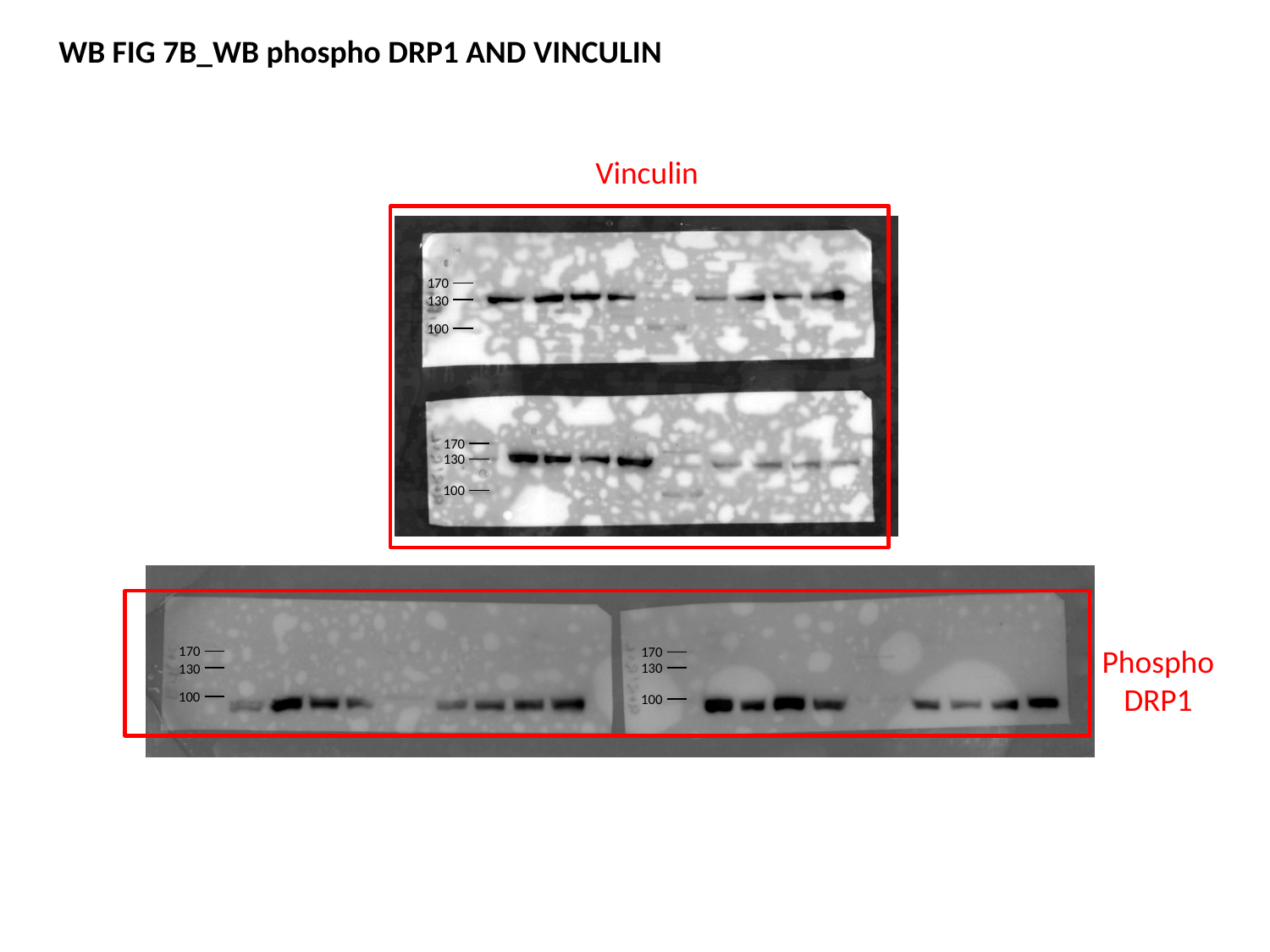

WB FIG 7B_WB phospho DRP1 AND VINCULIN
Vinculin
170
130
100
170
130
100
Phospho DRP1
170
170
130
130
100
100

Supplement: Supplementary file 10 — Source Data for Figure 7 [file EMMM-13-e13929-s002.zip › EMM-2021-13929_Fig7/EMM-2021-13929_Fig7B/EMM-2021-13929_Fig7B_withMARKERS_pDRP1-Vinculin.pptx]

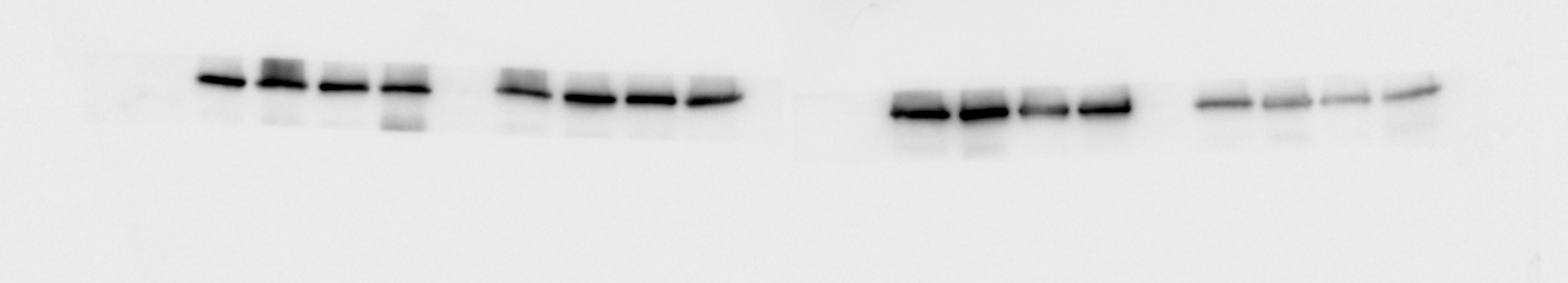

Supplement: Supplementary file 10 — Source Data for Figure 7 [file EMMM-13-e13929-s002.zip › EMM-2021-13929_Fig7/EMM-2021-13929_Fig7B/EMM-2021-13929_Fig7B_Vinculin_total S6.tif]

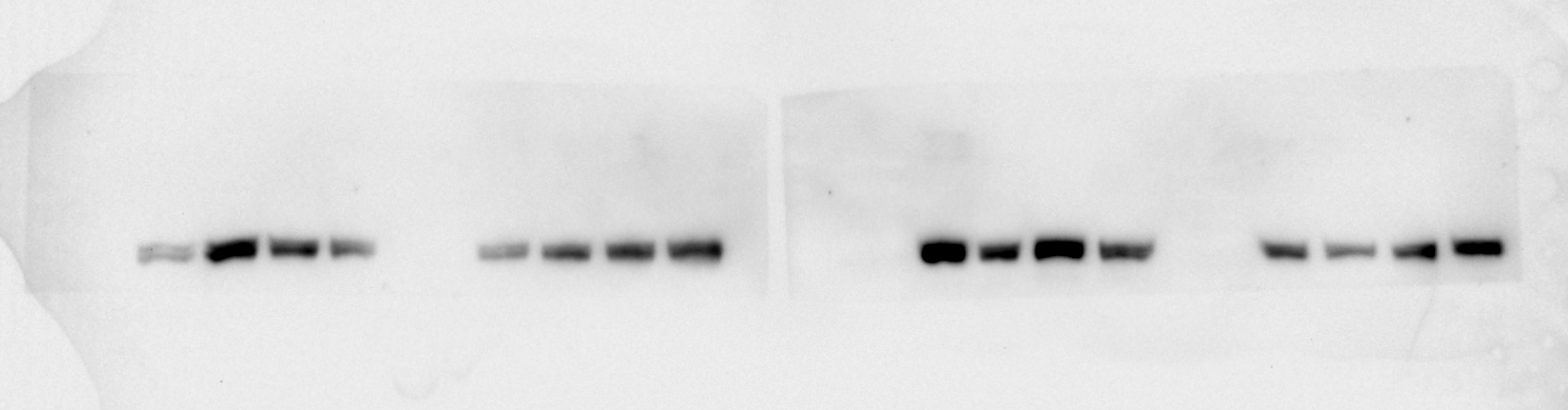

Supplement: Supplementary file 10 — Source Data for Figure 7 [file EMMM-13-e13929-s002.zip › EMM-2021-13929_Fig7/EMM-2021-13929_Fig7B/EMM-2021-13929_Fig7B_DRP1.tif]

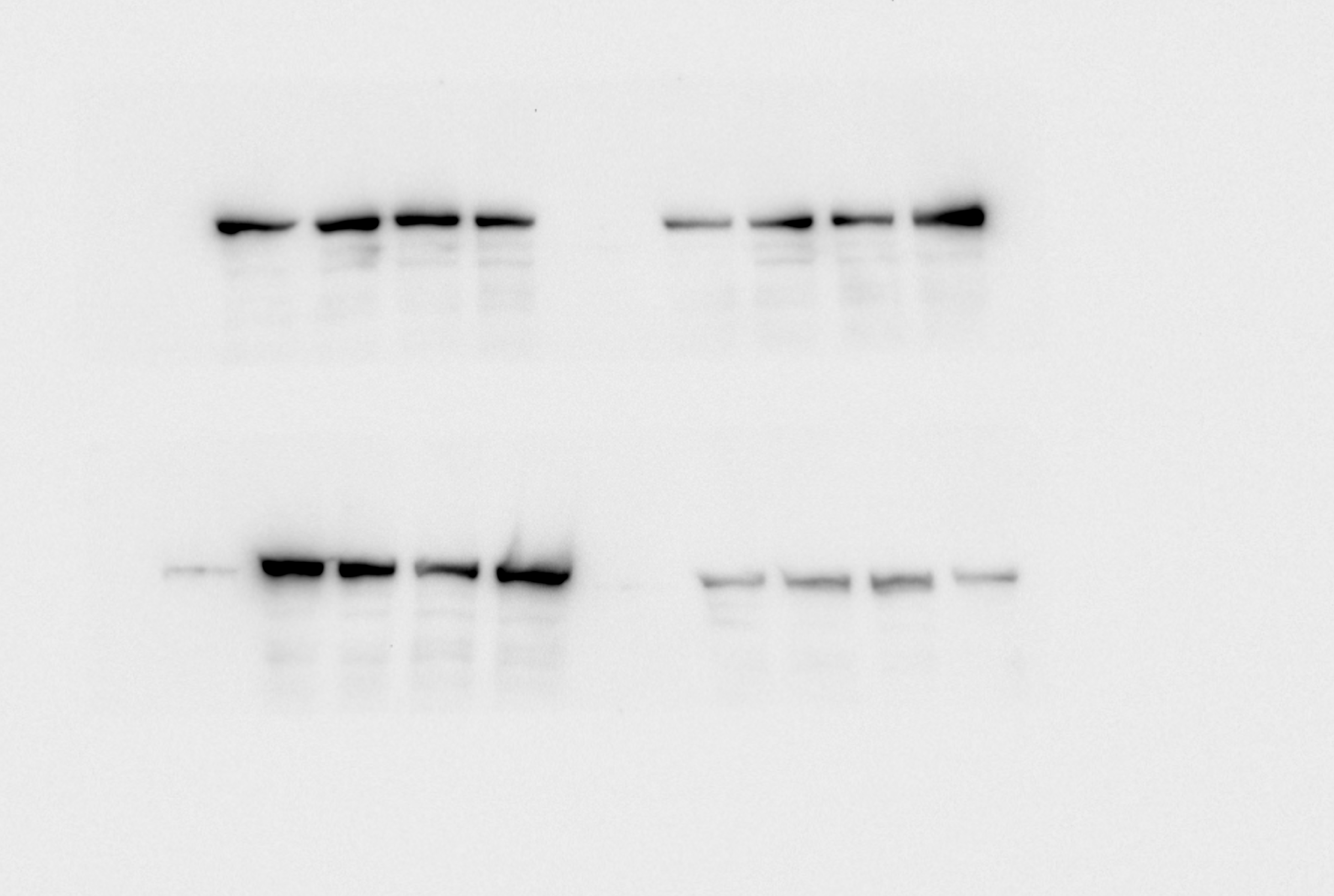

Supplement: Supplementary file 10 — Source Data for Figure 7 [file EMMM-13-e13929-s002.zip › EMM-2021-13929_Fig7/EMM-2021-13929_Fig7B/EMM-2021-13929_Fig7B_Vinculin_DRP1.tif]

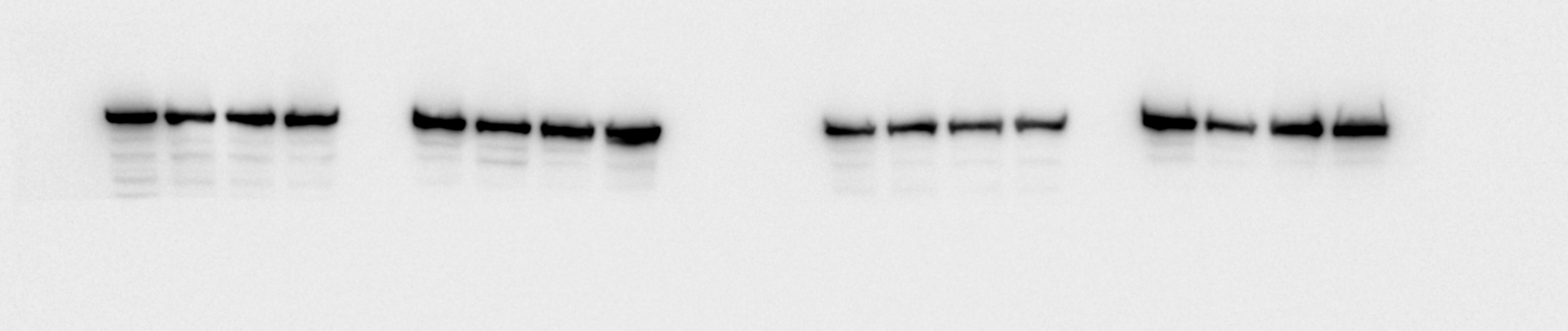

Supplement: Supplementary file 10 — Source Data for Figure 7 [file EMMM-13-e13929-s002.zip › EMM-2021-13929_Fig7/EMM-2021-13929_Fig7B/EMM-2021-13929_Fig7B_Vinculin_HRH1+S6.tif]

## Slide 1
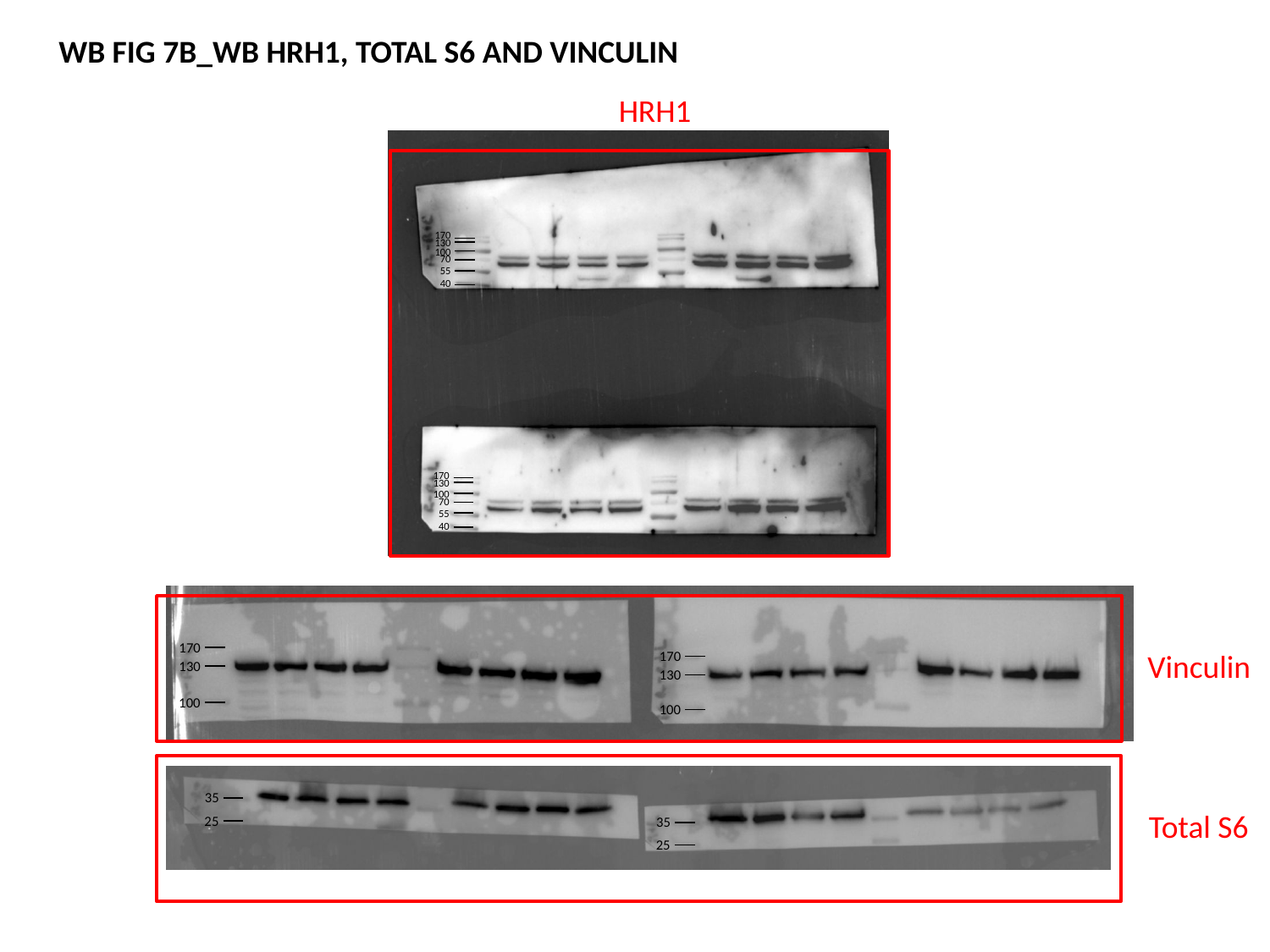

WB FIG 7B_WB HRH1, TOTAL S6 AND VINCULIN
HRH1
170
130
100
70
55
40
170
130
100
70
55
40
170
Vinculin
170
130
130
100
100
35
Total S6
25
35
25

Supplement: Supplementary file 10 — Source Data for Figure 7 [file EMMM-13-e13929-s002.zip › EMM-2021-13929_Fig7/EMM-2021-13929_Fig7B/EMM-2021-13929_Fig7B_withMARKERS_HRH1-TotalS6.pptx]

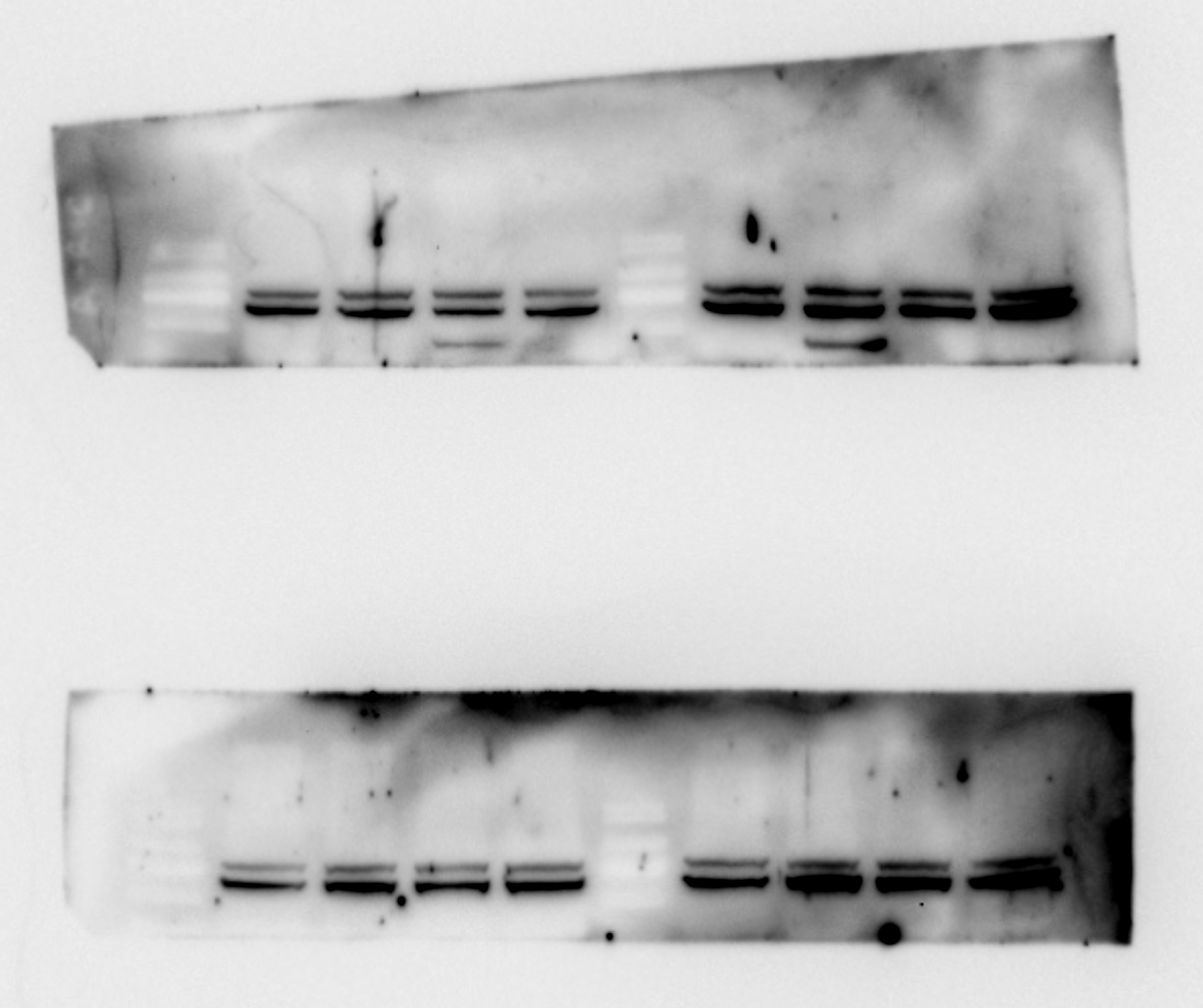

Supplement: Supplementary file 10 — Source Data for Figure 7 [file EMMM-13-e13929-s002.zip › EMM-2021-13929_Fig7/EMM-2021-13929_Fig7B/EMM-2021-13929_Fig7B_HRH1.tif]

## Slide 1
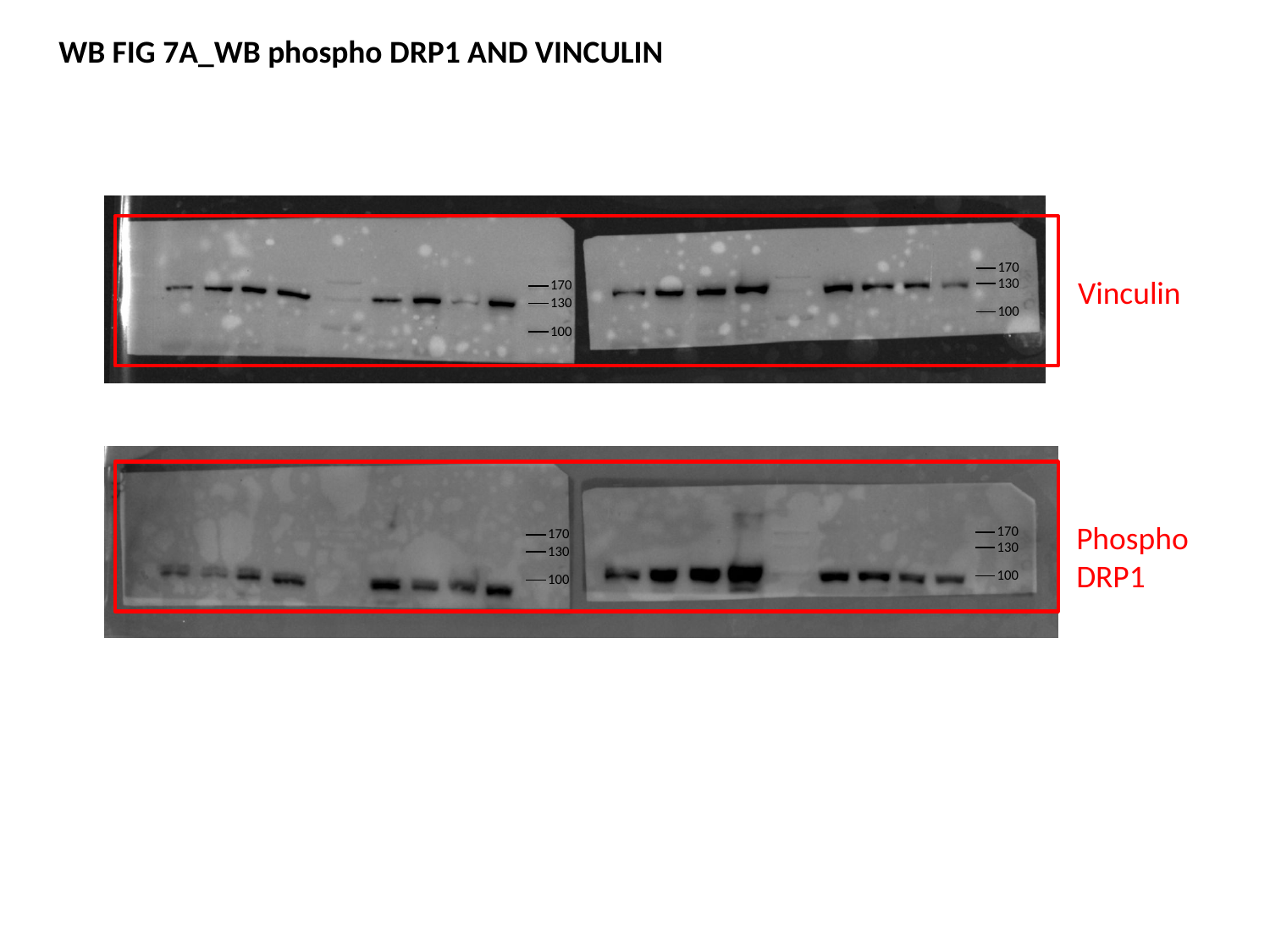

WB FIG 7A_WB phospho DRP1 AND VINCULIN
170
Vinculin
130
170
130
100
100
Phospho DRP1
170
170
130
130
100
100

Supplement: Supplementary file 10 — Source Data for Figure 7 [file EMMM-13-e13929-s002.zip › EMM-2021-13929_Fig7/EMM-2021-13929_Fig7A/EMM-2021-13929_Fig7A_withMARKERS_pDRP1.pptx]

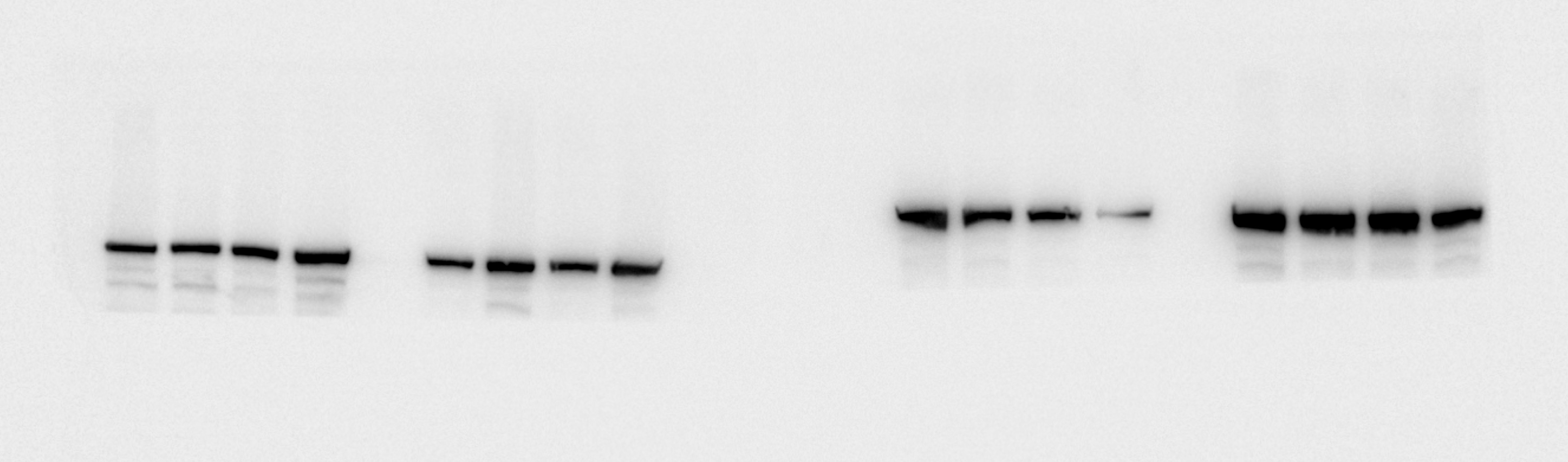

Supplement: Supplementary file 10 — Source Data for Figure 7 [file EMMM-13-e13929-s002.zip › EMM-2021-13929_Fig7/EMM-2021-13929_Fig7A/EMM-2021-13929_Fig7A_Fig 7A_Vinculin_HRH1.tif]

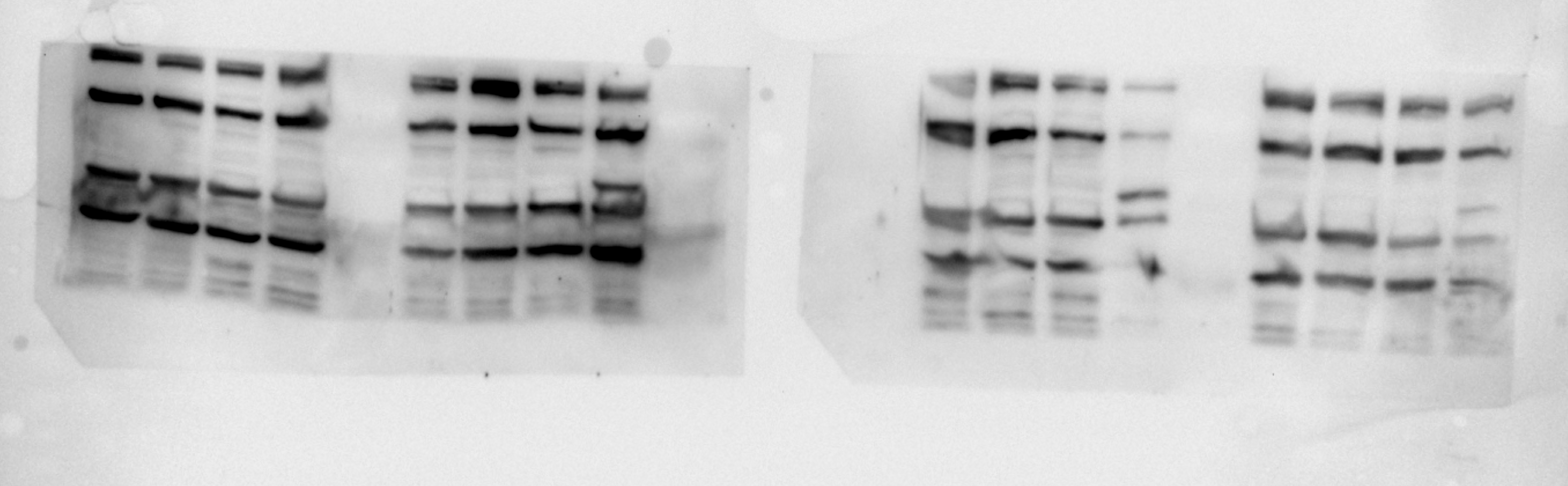

Supplement: Supplementary file 10 — Source Data for Figure 7 [file EMMM-13-e13929-s002.zip › EMM-2021-13929_Fig7/EMM-2021-13929_Fig7A/EMM-2021-13929_Fig7A_Fig 7A_HRH1.tif]

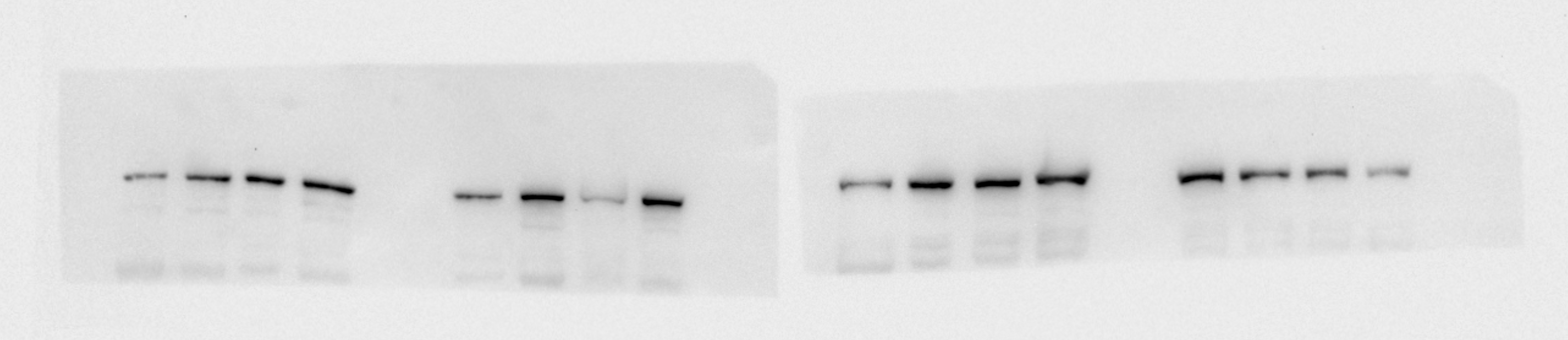

Supplement: Supplementary file 10 — Source Data for Figure 7 [file EMMM-13-e13929-s002.zip › EMM-2021-13929_Fig7/EMM-2021-13929_Fig7A/EMM-2021-13929_Fig7A_Fig 7A_Vinculin_DRP1.tif]

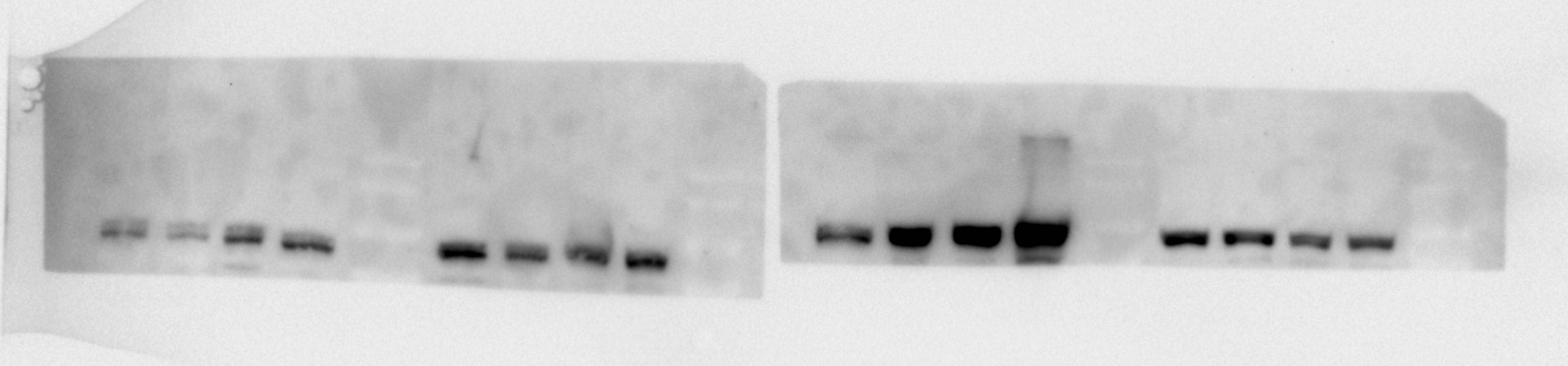

Supplement: Supplementary file 10 — Source Data for Figure 7 [file EMMM-13-e13929-s002.zip › EMM-2021-13929_Fig7/EMM-2021-13929_Fig7A/EMM-2021-13929_Fig7A_Fig 7A_DRP1.tif]

## Slide 1
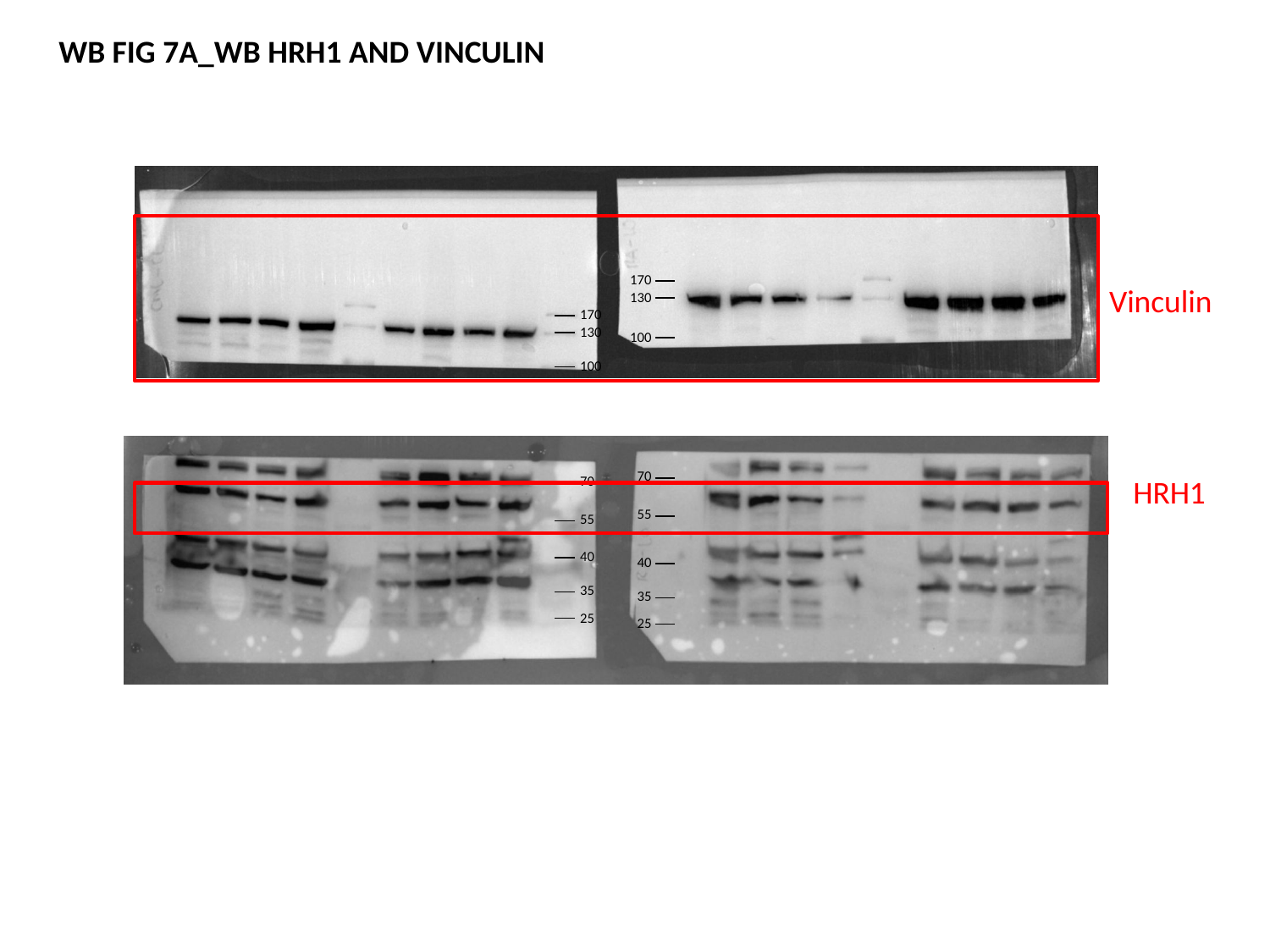

WB FIG 7A_WB HRH1 AND VINCULIN
170
130
100
70
55
40
35
25
Vinculin
170
130
100
70
HRH1
55
40
35
25

Supplement: Supplementary file 10 — Source Data for Figure 7 [file EMMM-13-e13929-s002.zip › EMM-2021-13929_Fig7/EMM-2021-13929_Fig7A/EMM-2021-13929_Fig7A_withMARKERS_HRH1.pptx]

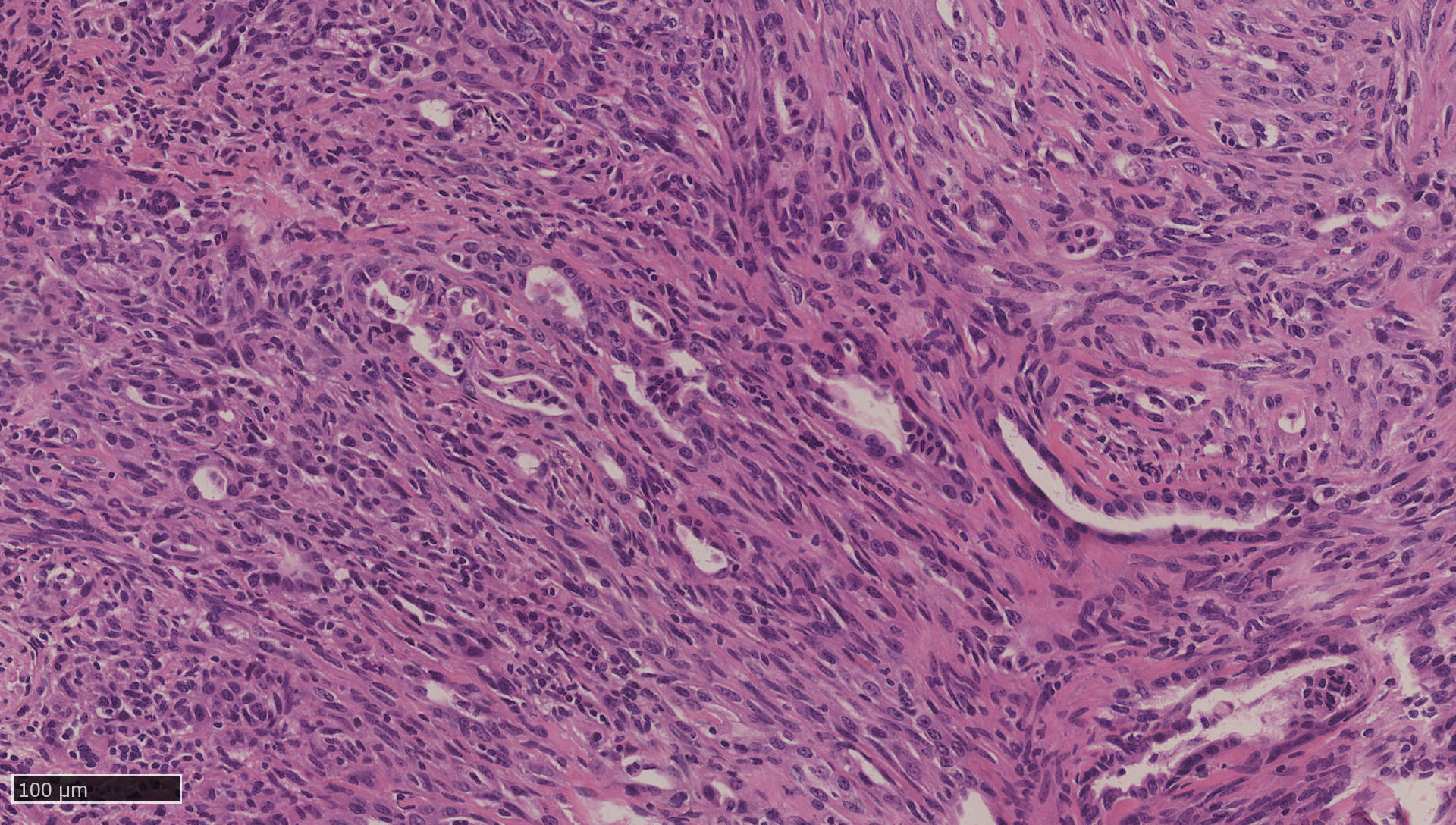

Supplement: Supplementary file 10 — Source Data for Figure 7 [file EMMM-13-e13929-s002.zip › EMM-2021-13929_Fig7/EMM-2021-13929_Fig7F/EMM-2021-13929_Fig7F_RAPAMYCIN+LORATADINE_G1L HE 200X.jpg]

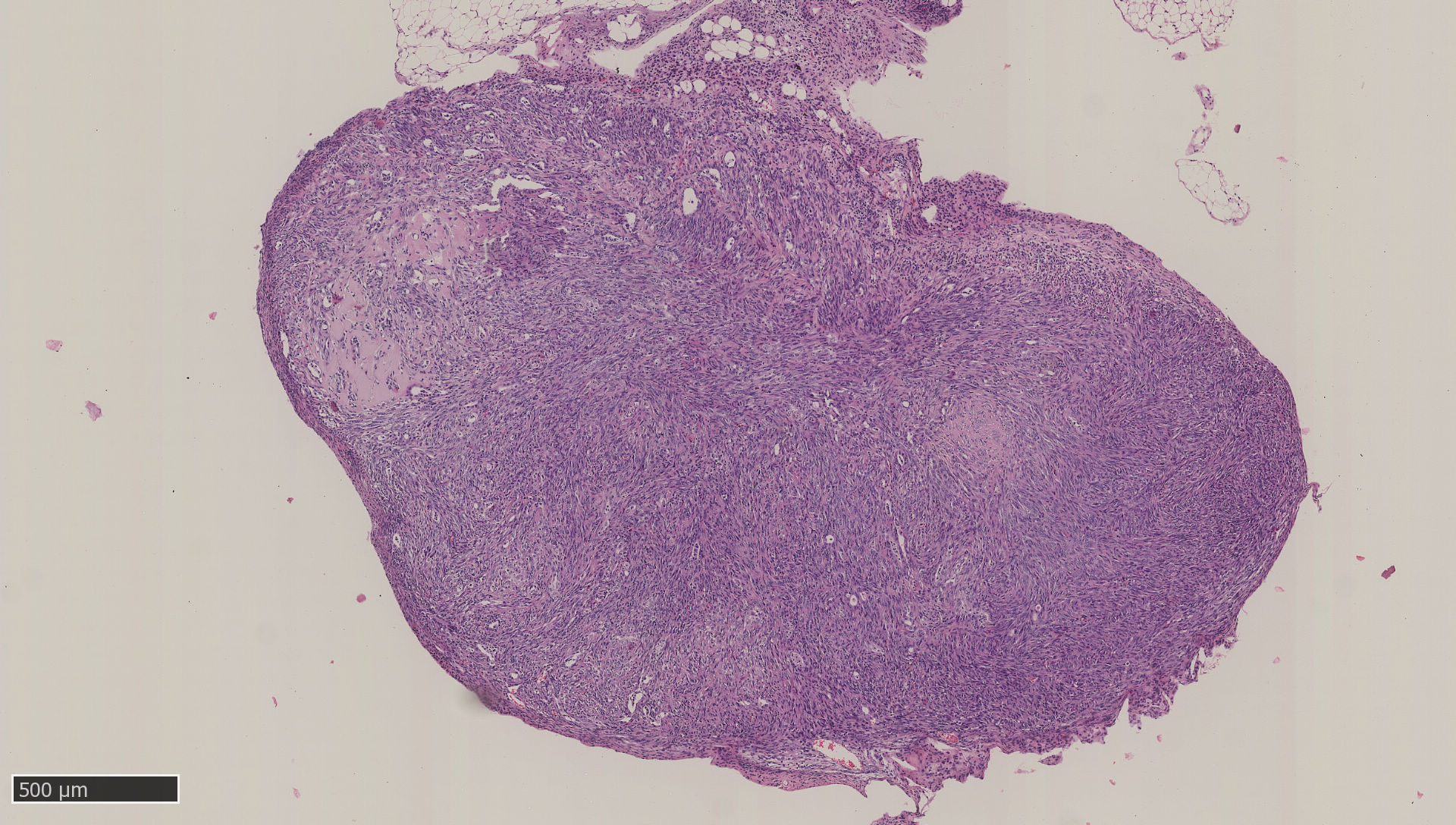

Supplement: Supplementary file 10 — Source Data for Figure 7 [file EMMM-13-e13929-s002.zip › EMM-2021-13929_Fig7/EMM-2021-13929_Fig7F/EMM-2021-13929_Fig7F_RAPAMYCIN+RASAGILINE_F2R HE 40X.jpg]

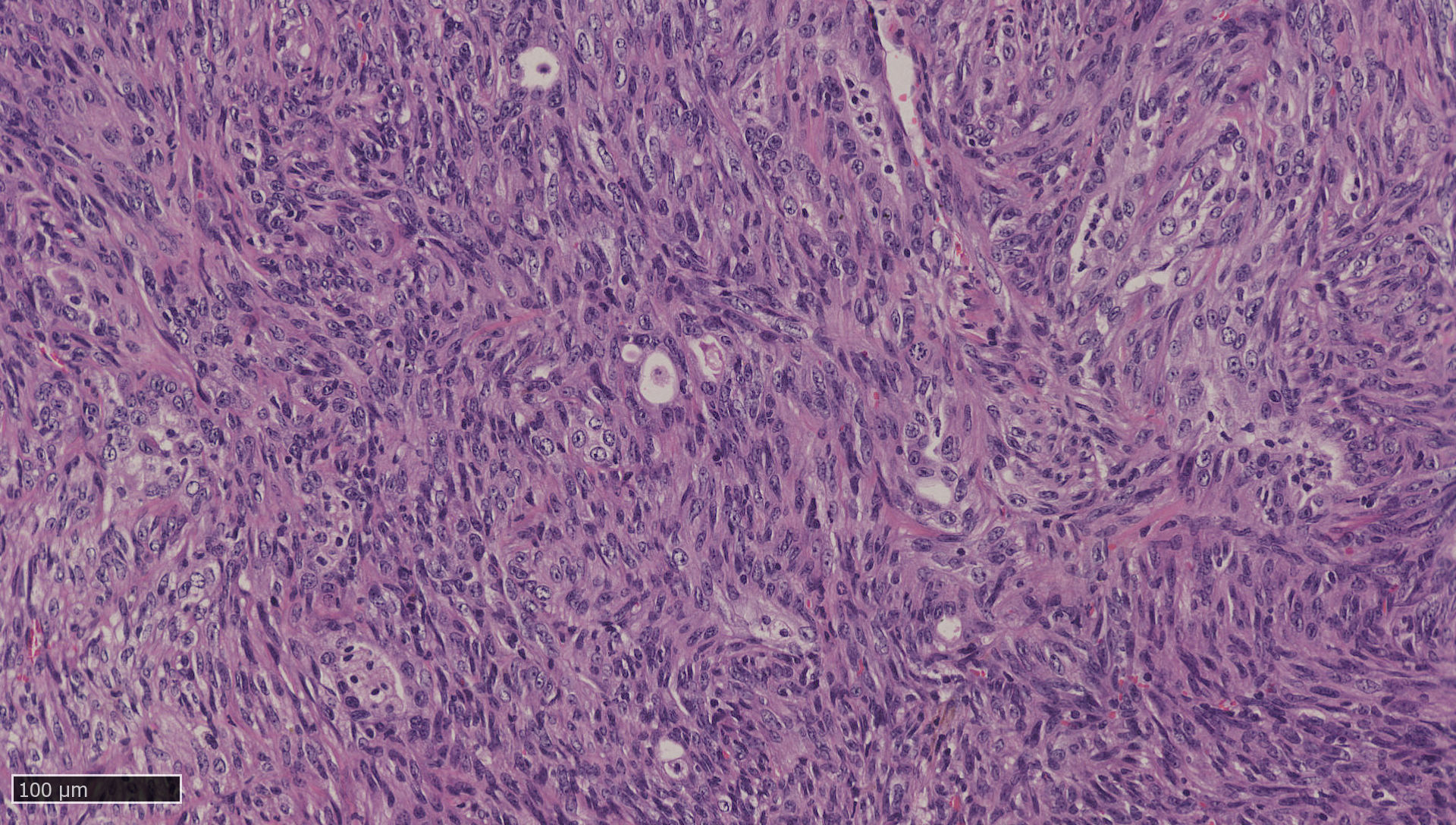

Supplement: Supplementary file 10 — Source Data for Figure 7 [file EMMM-13-e13929-s002.zip › EMM-2021-13929_Fig7/EMM-2021-13929_Fig7F/EMM-2021-13929_Fig7F_RAPAMYCIN+RASAGILINE_F2R HE 200X.jpg]

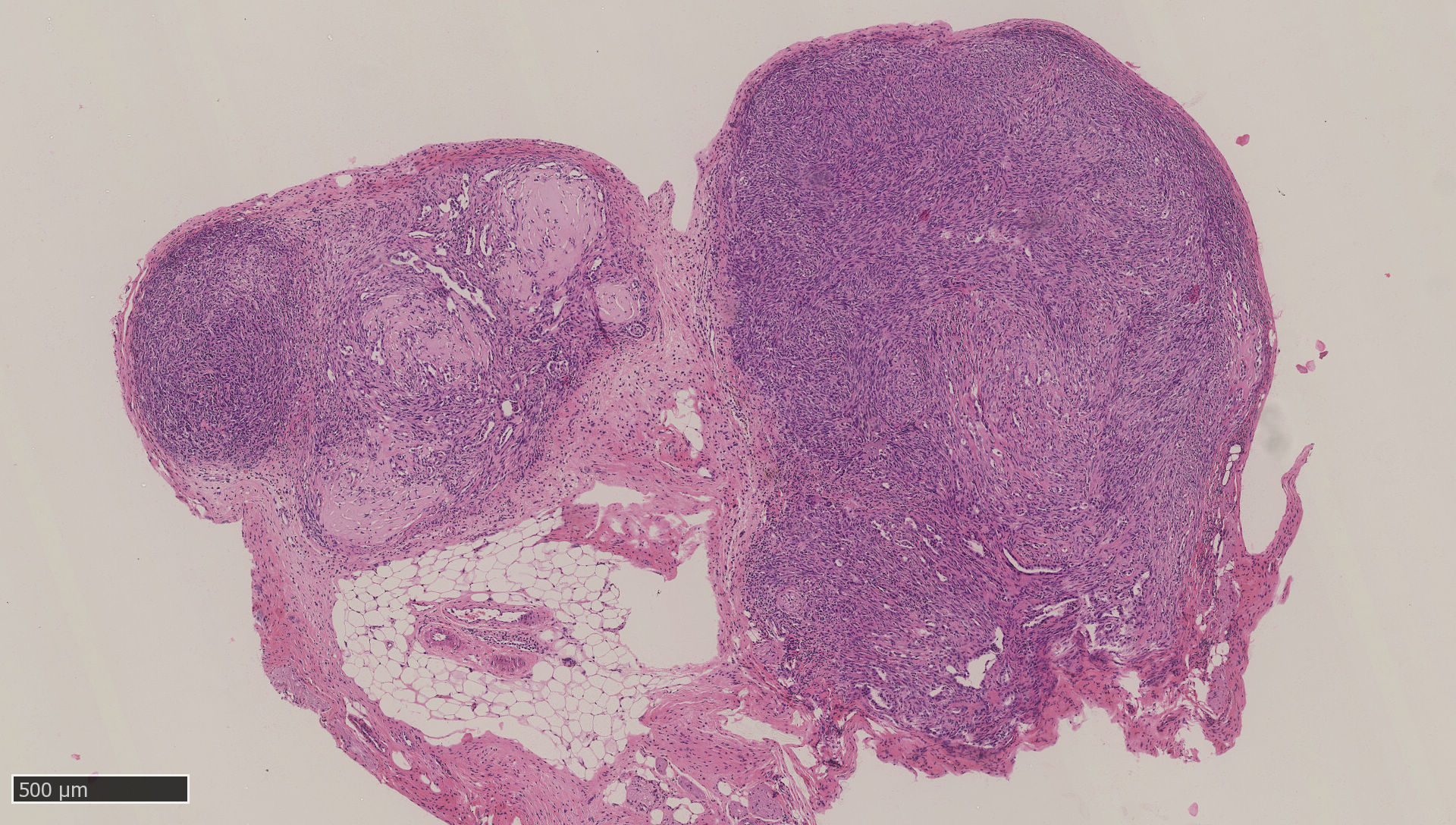

Supplement: Supplementary file 10 — Source Data for Figure 7 [file EMMM-13-e13929-s002.zip › EMM-2021-13929_Fig7/EMM-2021-13929_Fig7F/EMM-2021-13929_Fig7F_RAPAMYCIN+LORATADINE_G1L HE 40X.jpg]

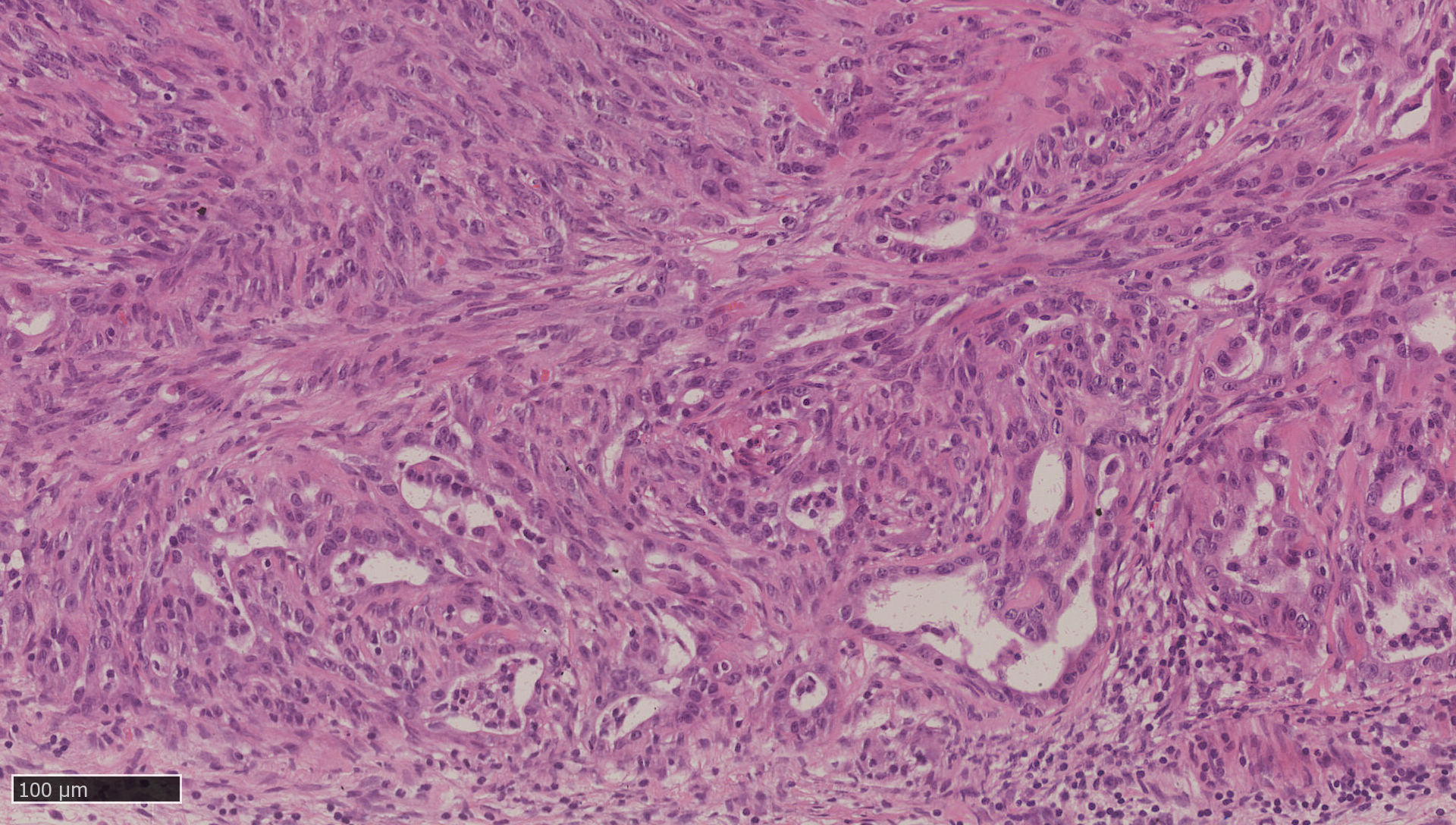

Supplement: Supplementary file 10 — Source Data for Figure 7 [file EMMM-13-e13929-s002.zip › EMM-2021-13929_Fig7/EMM-2021-13929_Fig7F/EMM-2021-13929_Fig7F_RAPAMYCIN+CLORGYLINE_G2R HE 200X.jpg]

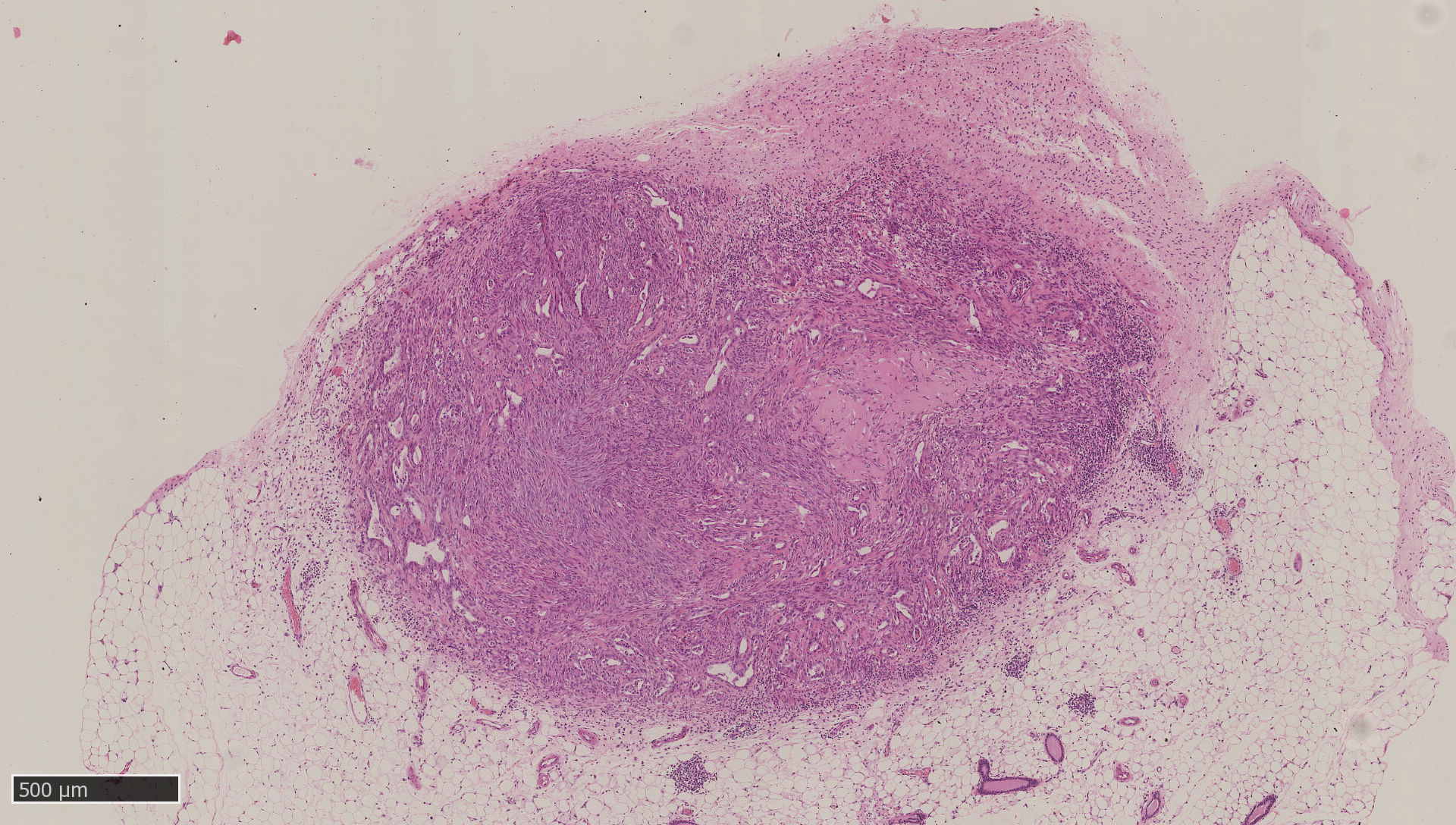

Supplement: Supplementary file 10 — Source Data for Figure 7 [file EMMM-13-e13929-s002.zip › EMM-2021-13929_Fig7/EMM-2021-13929_Fig7F/EMM-2021-13929_Fig7F_RAPAMYCIN+CLORGYLINE_G2R HE 40X.jpg]
