# Supplementary material for: Histamine signaling and metabolism identify potential biomarkers and therapies for lymphangioleiomyomatosis
Source: EMBO Mol Med. 2021 Aug 11;13(9):e13929. doi: 10.15252/emmm.202113929 (PMC8422079; doi:10.15252/emmm.202113929)
Supplement: Supplementary file 7 — Source Data for Figure 4 [file EMMM-13-e13929-s004.zip › EMM-2021-13929_Fig4/EMM-2021-13929_Fig4B/EMM-2021-13929_Fig4B_MitoSOX.pdf]

FC18246 2018.05.11 CTRL MEF++ 025

FC18246 2018.05.11 CTRL MEF++ 025 - Imported

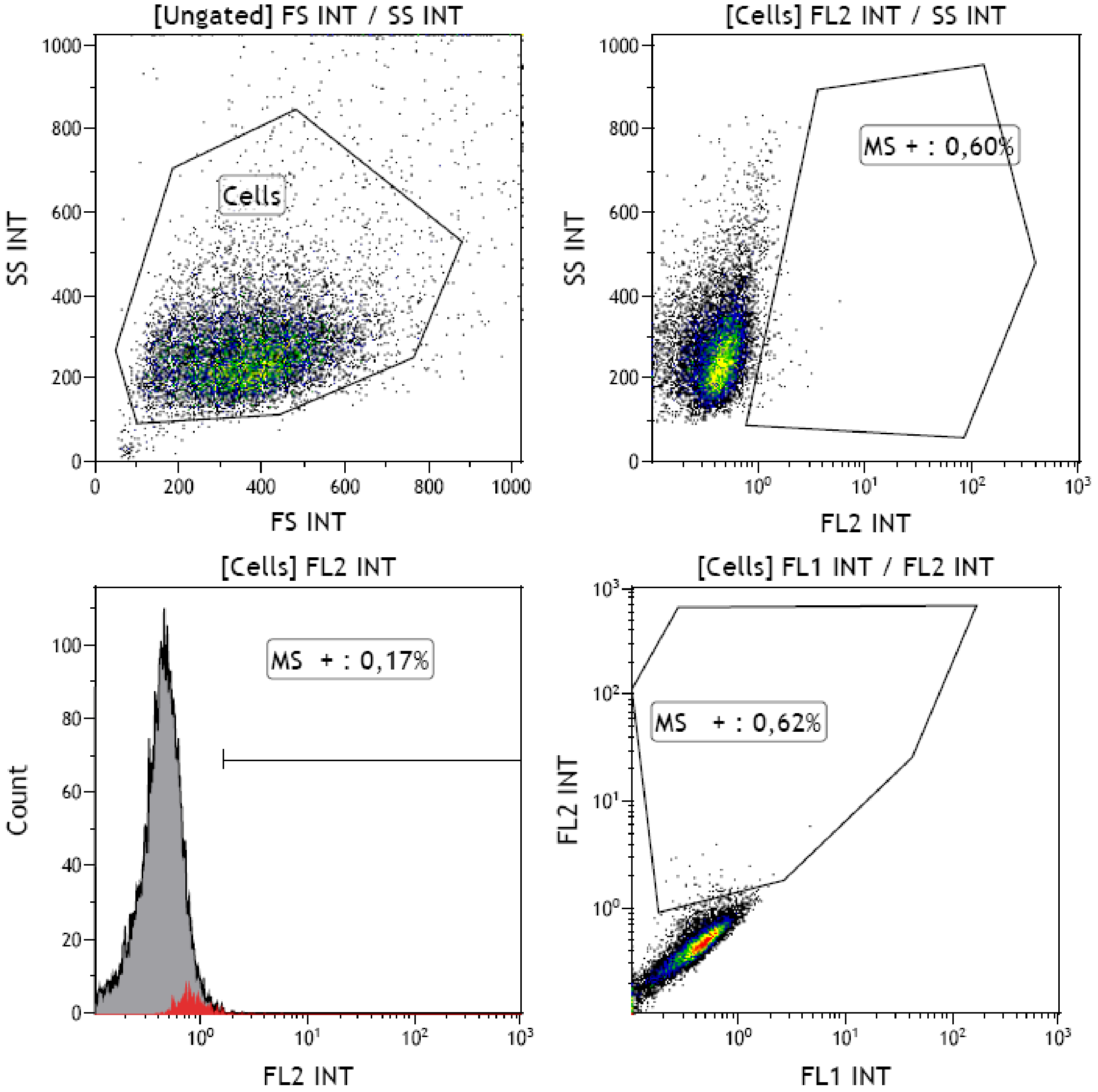

Gate	%Gated	X-AMean
All	100,00	0,47
MS +	0,17	2,49

Gate	%Gated	Y-AMean
All	100,00	0,47
MS +	0,62	1,70

FC18246 2018.05.11 CTRL MEF++ 025

FC18246 2018.05.11 CTRL MEF++ 025 - Imported

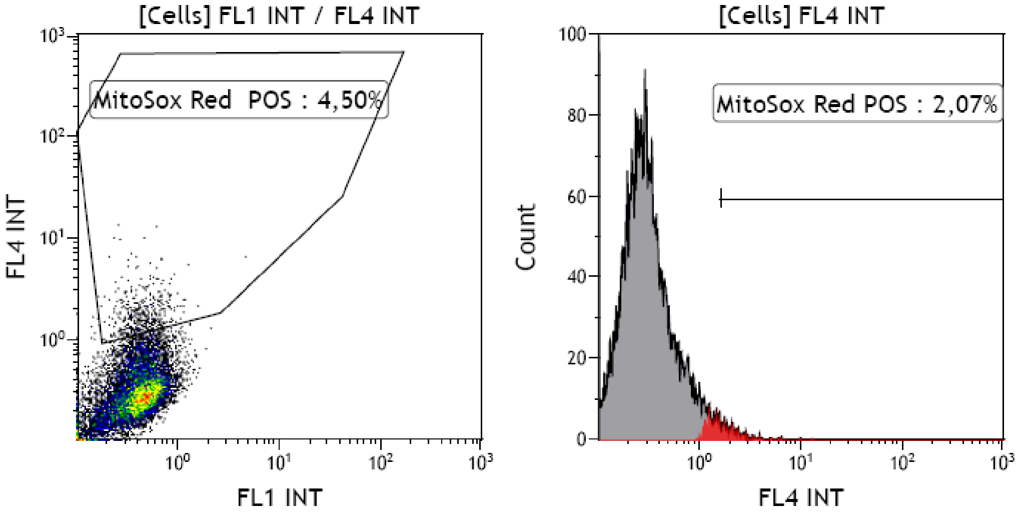

Gate	%Gated	Y-AMean
All	100,00	0,41
MitoSox Red POS	4,50	1,99

Gate	%Gated	X-AMean
All	100,00	0,41
MitoSox Red POS	2,07	2,76

FC18246 2018.05.11 MitoSox Red MEF++ 026

FC18246 2018.05.11 CTRL MEF++ 025 - Imported

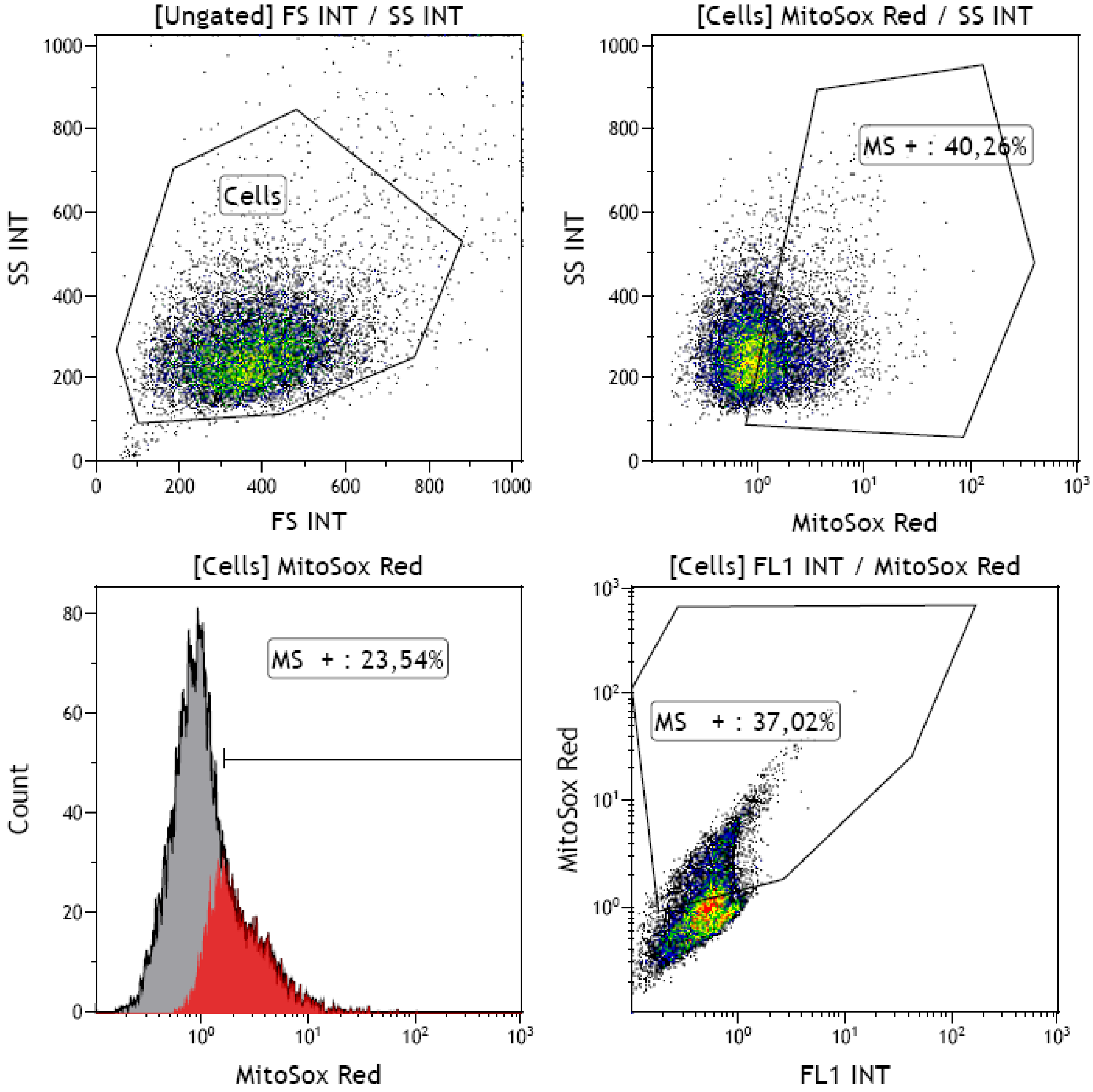

Gate	%Gated	X-AMean
All	100,00	1,64
MS +	23,54	4,16

Gate	%Gated	Y-AMean
All	100,00	1,64
MS +	37,02	3,14

FC18246 2018.05.11 MitoSox Red MEF++ 026
FC18246 2018.05.11 CTRL MEF++ 025 - Imported

[Cells] FL1 INT / FL4 INT

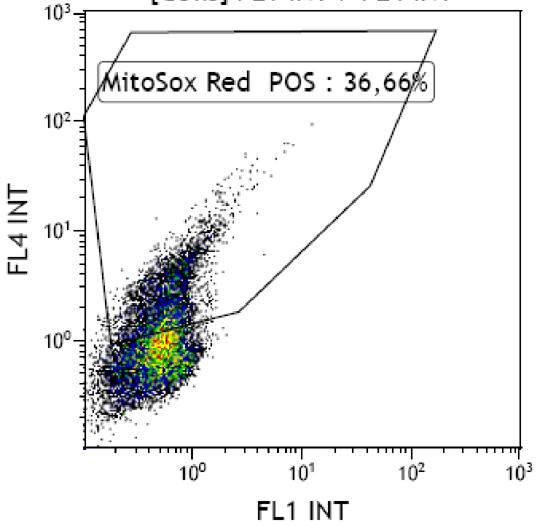

[Cells] FL4 INT

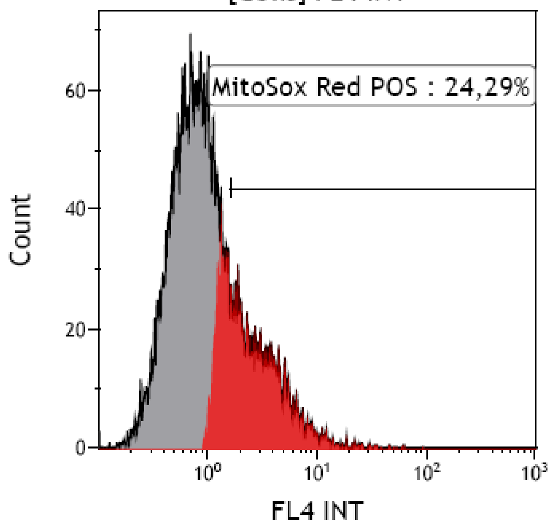

Gate	%Gated	Y-AMean
All	100,00	1,65
MitoSox Red POS	36,66	3,33

Gate	%Gated	X-AMean
All	100,00	1,65
MitoSox Red POS	24,29	4,34

FC18246 2018.05.11 MitoSox Red MEF-- 029
FC18246 2018.05.11 CTRL MEF++ 025 - Imported

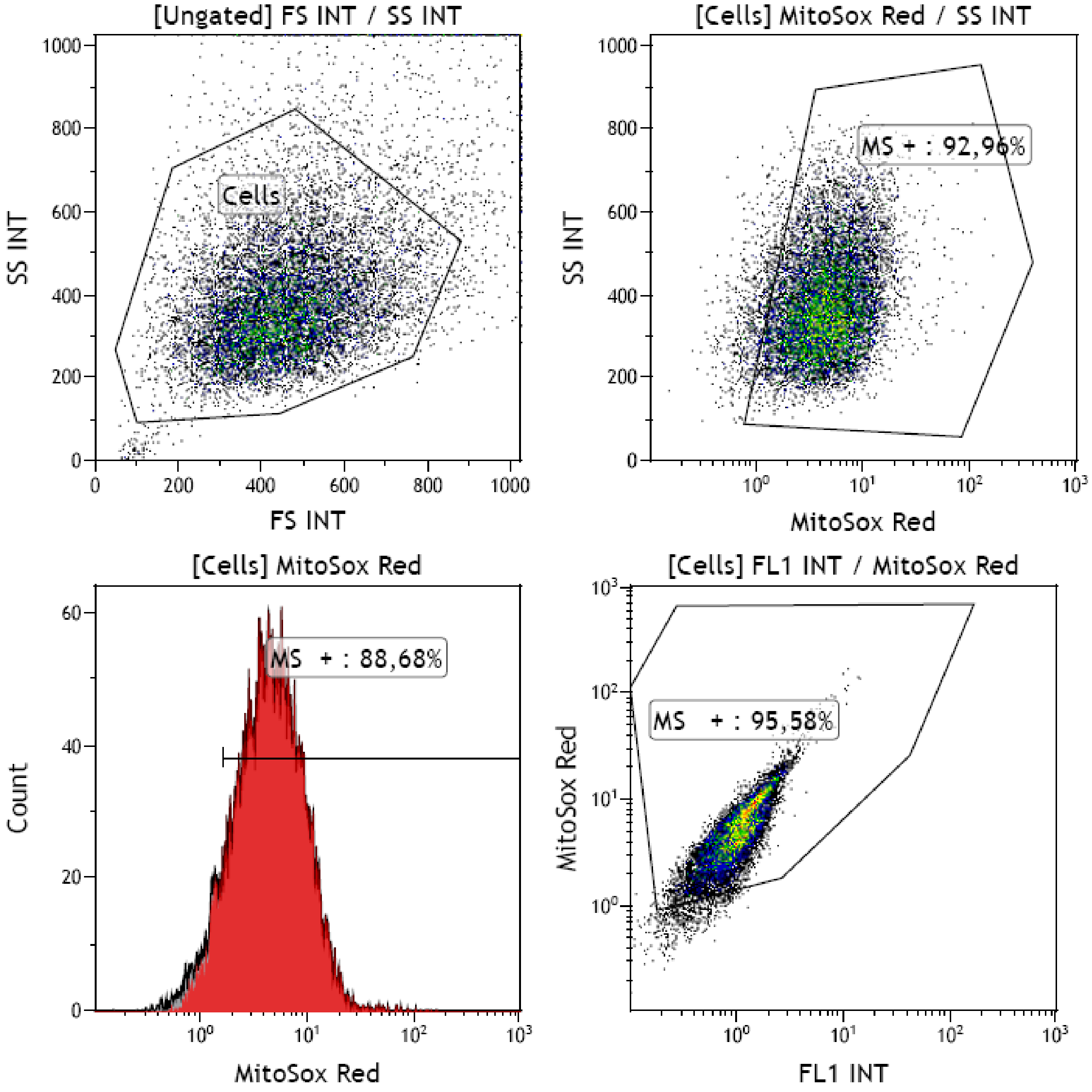

Gate	%Gated	X-AMean
All	100,00	5,76
MS +	88,68	6,35

Gate	%Gated	Y-AMean
All	100,00	5,76
MS +	95,58	5,99

FC18246 2018.05.11 MitoSox Red MEF-- 029
FC18246 2018.05.11 CTRL MEF++ 025 - Imported

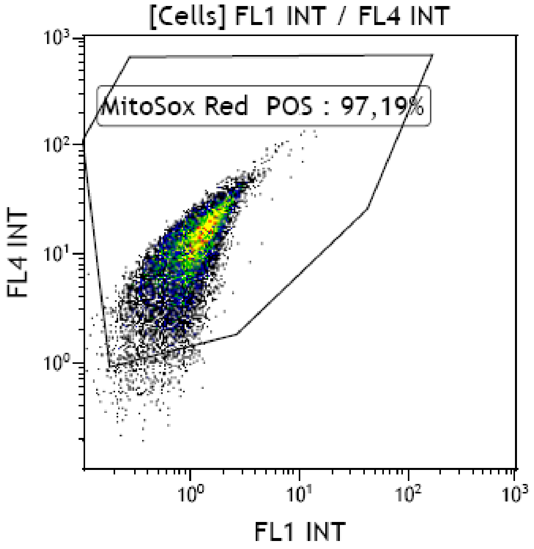

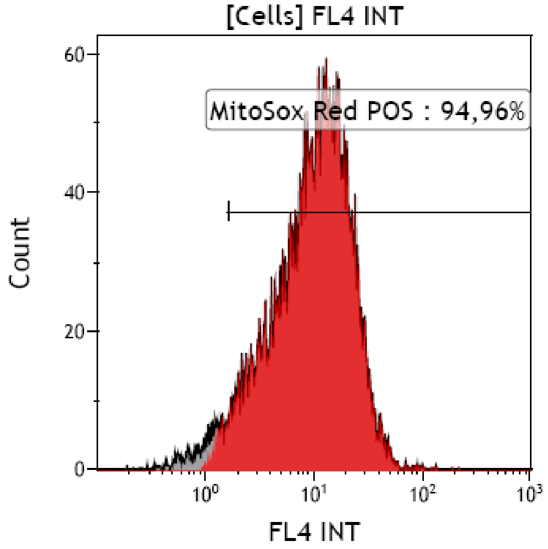

Gate	%Gated	Y-AMean
All	100,00	12,38
MitoSox Red POS	97,19	12,71

Gate	%Gated	X-AMean
All	100,00	12,38
MitoSox Red POS	94,96	12,98
