# Supplementary material for: Histamine signaling and metabolism identify potential biomarkers and therapies for lymphangioleiomyomatosis
Source: EMBO Mol Med. 2021 Aug 11;13(9):e13929. doi: 10.15252/emmm.202113929 (PMC8422079; doi:10.15252/emmm.202113929)
Supplement: Supplementary file 7 — Source Data for Figure 4 [file EMMM-13-e13929-s004.zip › EMM-2021-13929_Fig4/EMM-2021-13929_Fig4C/EMM-2021-13929_Fig4C_MitoTracker.pdf]

FC18266 2018.05.24 CTRL MEF++ 002
FC18246 2018.05.11 CTRL MEF++ 025 - Imported

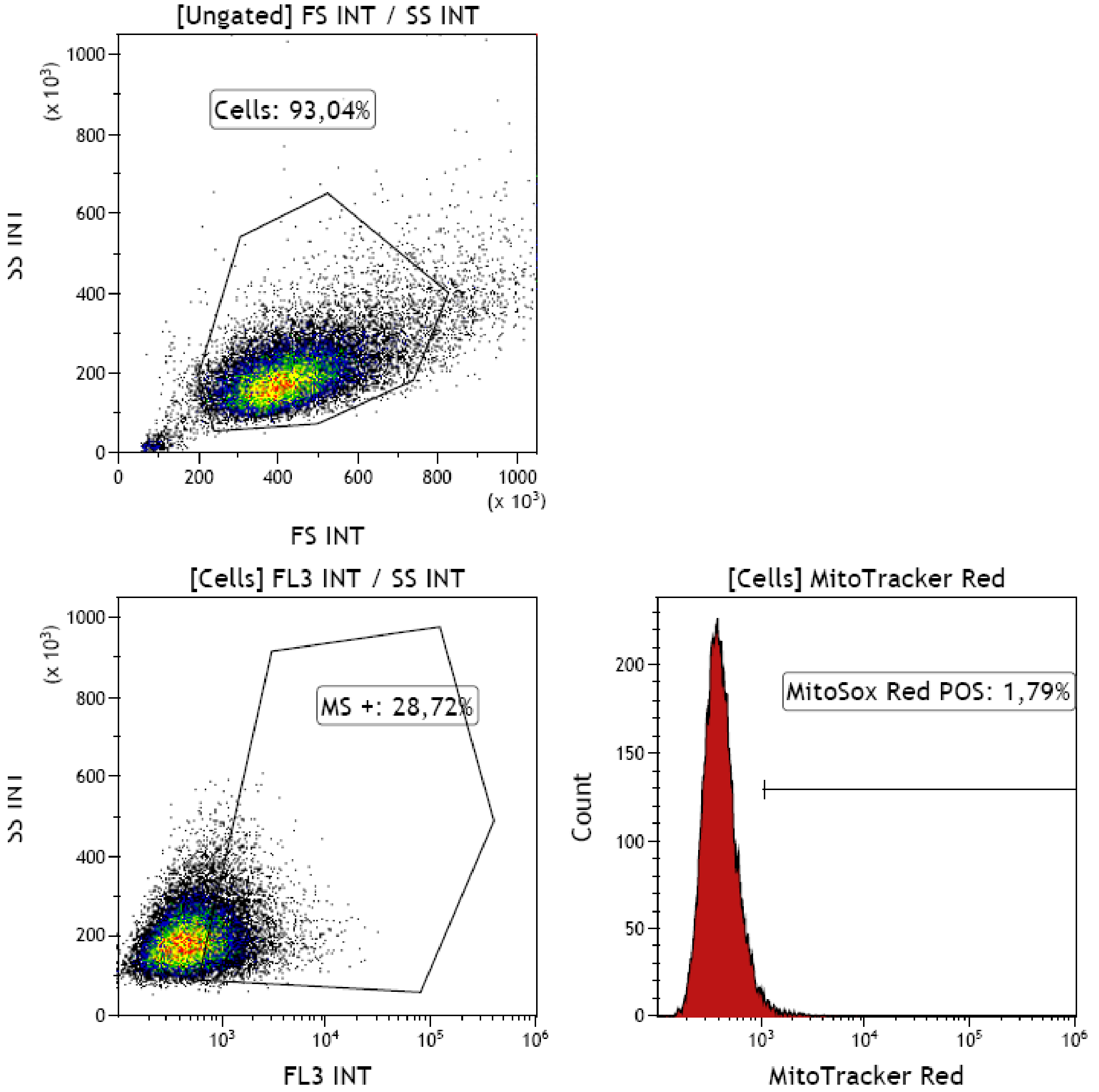

Gate	%Gated	X-AMean
All	100,00	458,41
MitoSox Red POS	1,79	1.722,45

FC18266 2018.05.24 CTRL MEF++ 002
FC18246 2018.05.11 CTRL MEF++ 025 - Imported

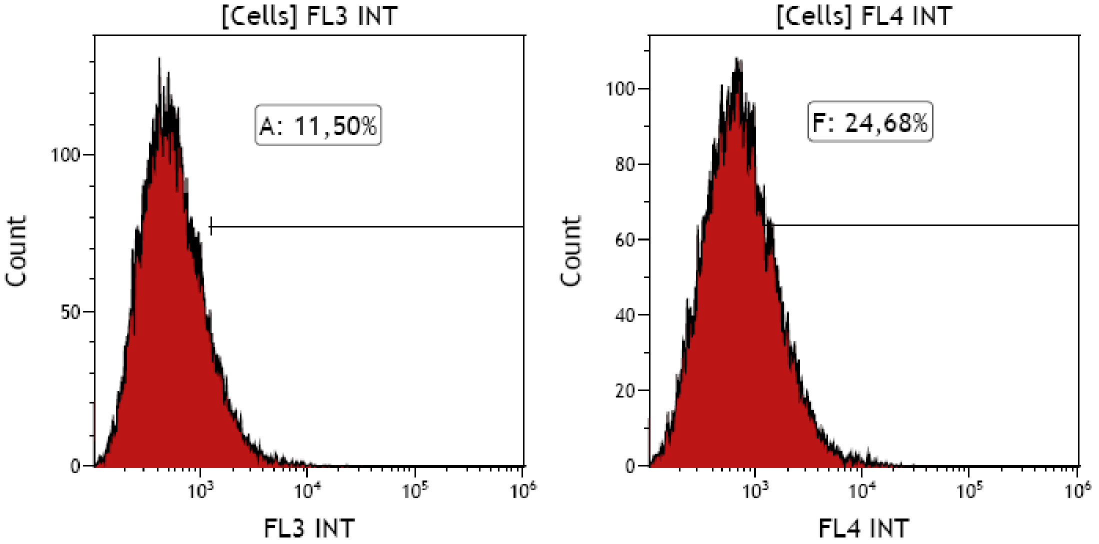

Gate	%Gated	X-AMean
All	100,00	761,55
A	11,50	2.416,03

Gate	%Gated	X-AMean
All	100,00	1.080,92
F	24,68	2.533,90

FC18266 2018.05.24 MitoTr Red MEF++ 003
FC18246 2018.05.11 CTRL MEF++ 025 - Imported

[Ungated] FS INT / SS INT

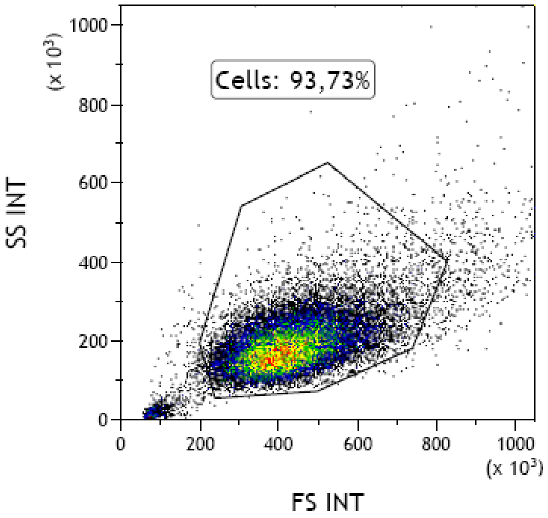

[Cells] FL3 INT / SS INT

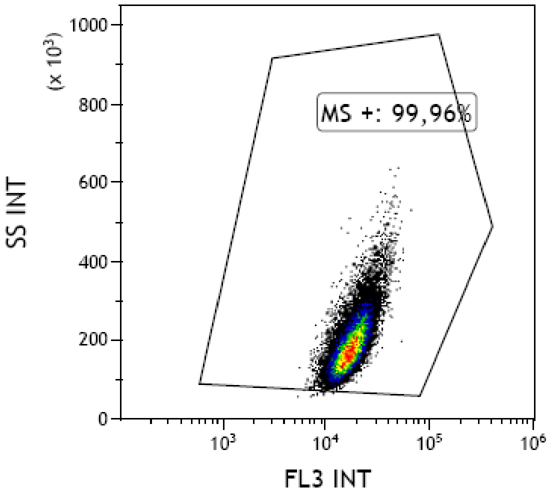

[Cells] MitoTracker Red

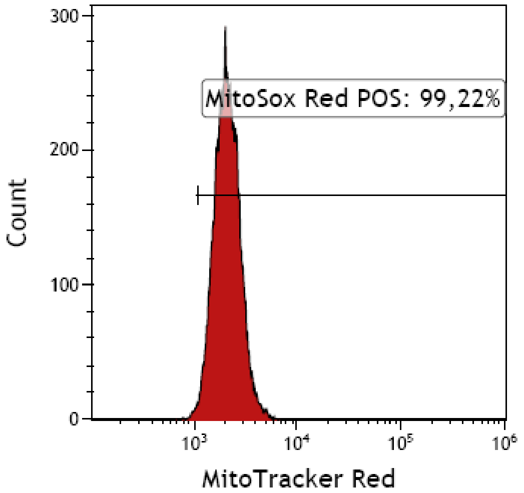

Gate	%Gated	X-AMean
All	100,00	2.213,57
MitoSox Red POS	99,22	2.223,04

FC18266 2018.05.24 MitoTr Red MEF++ 003
FC18246 2018.05.11 CTRL MEF++ 025 - Imported

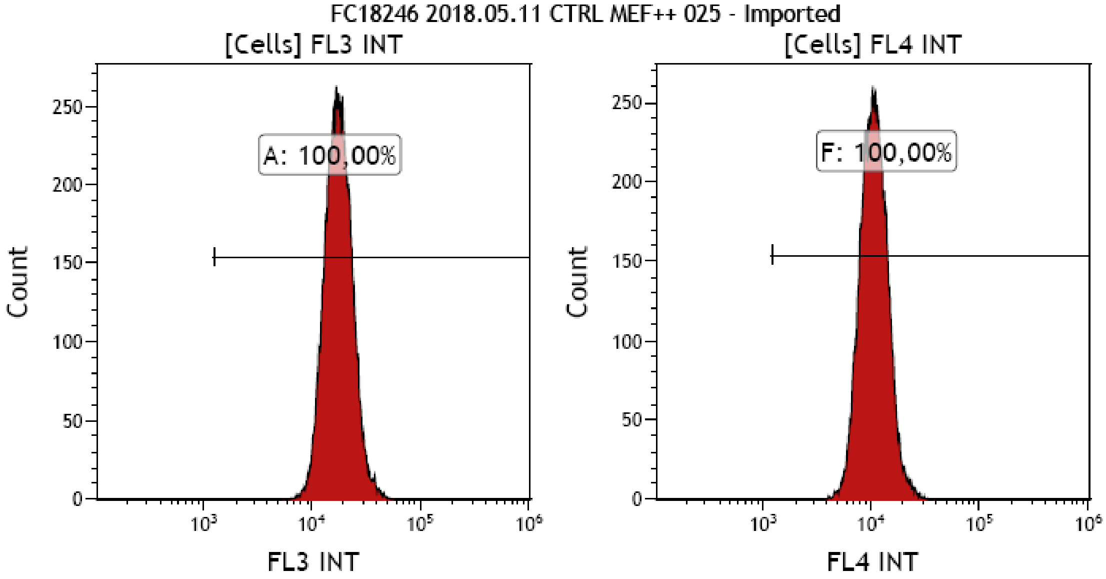

Gate	%Gated	X-AMean
All	100,00	18.582,33
A	100,00	18.582,33

Gate	%Gated	X-AMean
All	100,00	11.169,14
F	100,00	11.169,14

FC18266 2018.05.24 MitoTr Red MEF-- 006
FC18246 2018.05.11 CTRL MEF++ 025 - Imported

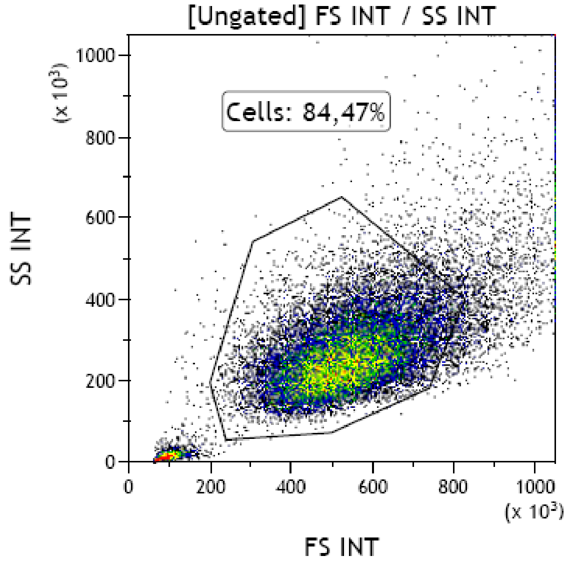

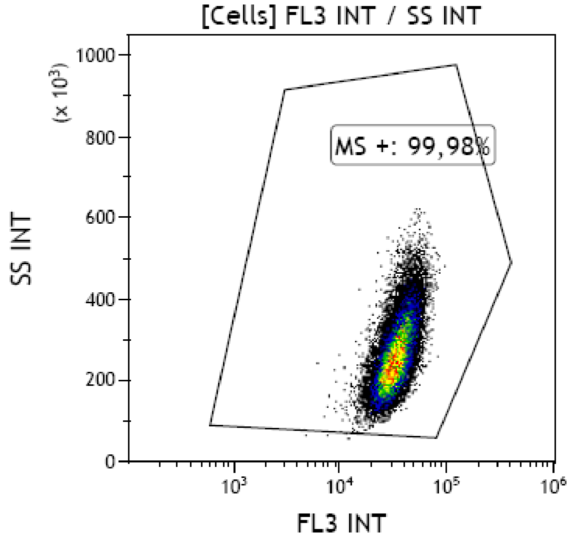

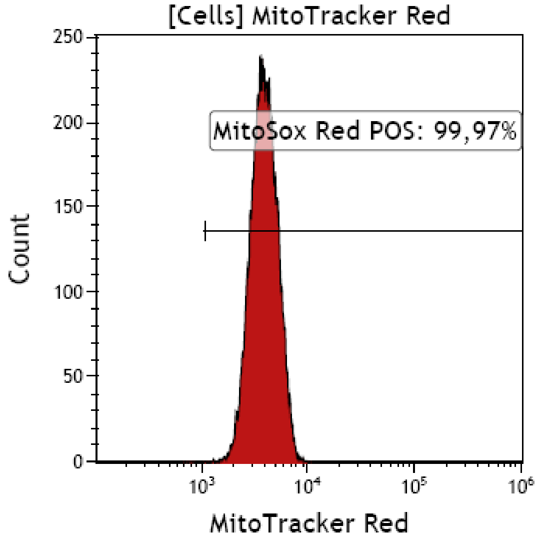

Gate	%Gated	X-AMean
All	100,00	4.074,54
MitoSox Red POS	99,97	4.075,58

FC18266 2018.05.24 MitoTr Red MEF-- 006
FC18246 2018.05.11 CTRL MEF++ 025 - Imported

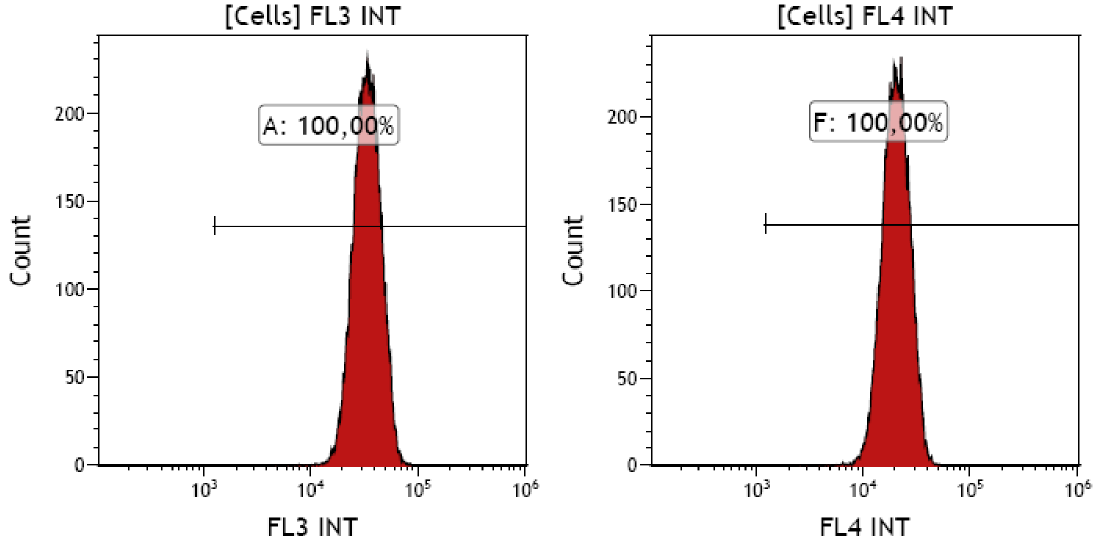

Gate	%Gated	X-AMean
All	100,00	35.063,05
A	100,00	35.063,05

Gate	%Gated	X-AMean
All	100,00	21.150,31
F	100,00	21.150,31
