# Supplementary material for: Histamine signaling and metabolism identify potential biomarkers and therapies for lymphangioleiomyomatosis
Source: EMBO Mol Med. 2021 Aug 11;13(9):e13929. doi: 10.15252/emmm.202113929 (PMC8422079; doi:10.15252/emmm.202113929)
Supplement: Supplementary file 9 — Source Data for Figure 6 [file EMMM-13-e13929-s010.zip › EMM-2021-13929_Fig6/EMM-2021-13929_Fig6B-C/EMM-2021-13929_Fig6B-C.pptx]

## Slide 1
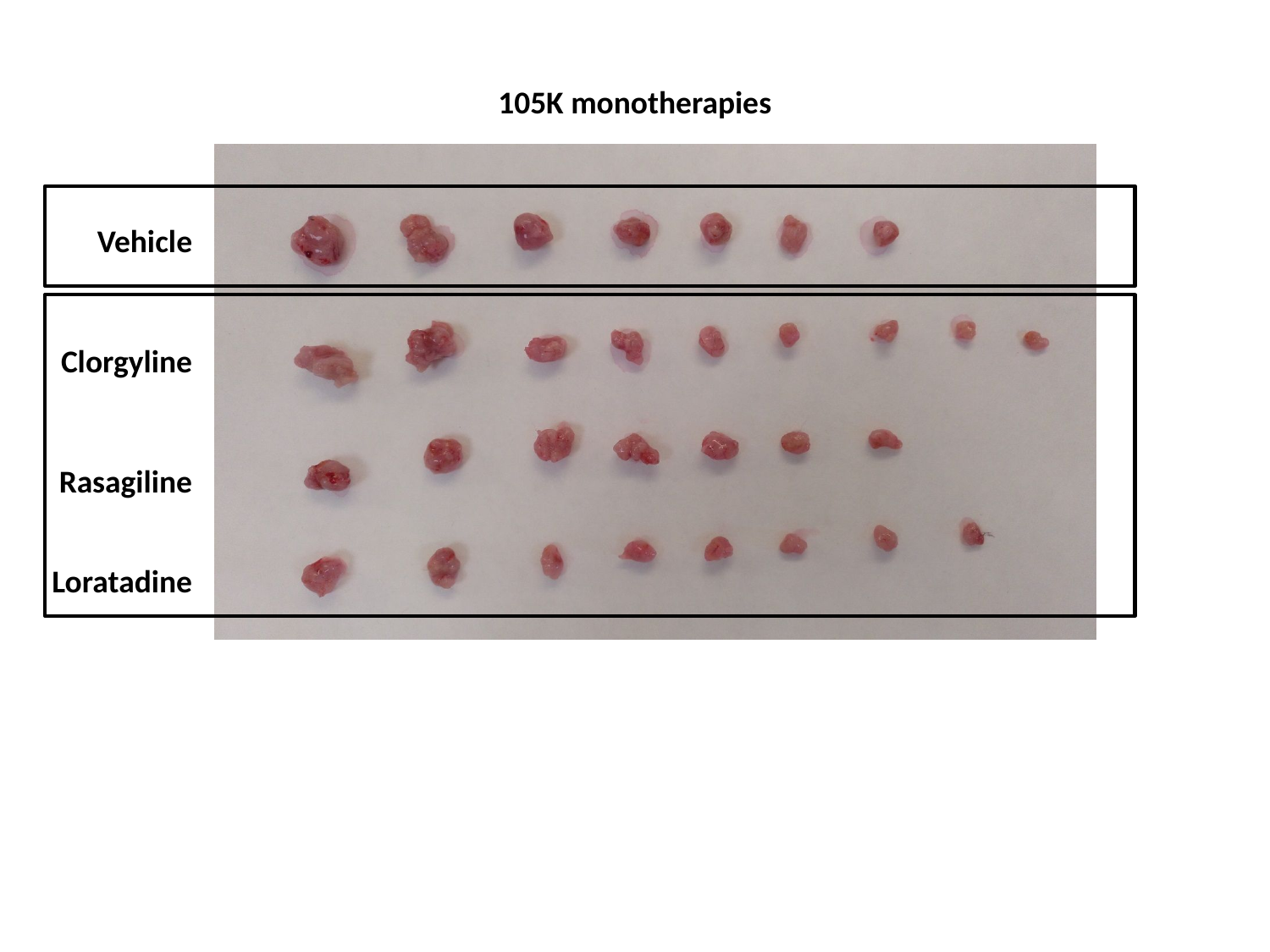

105K monotherapies
Vehicle
Clorgyline
Rasagiline
Loratadine

## Slide 2
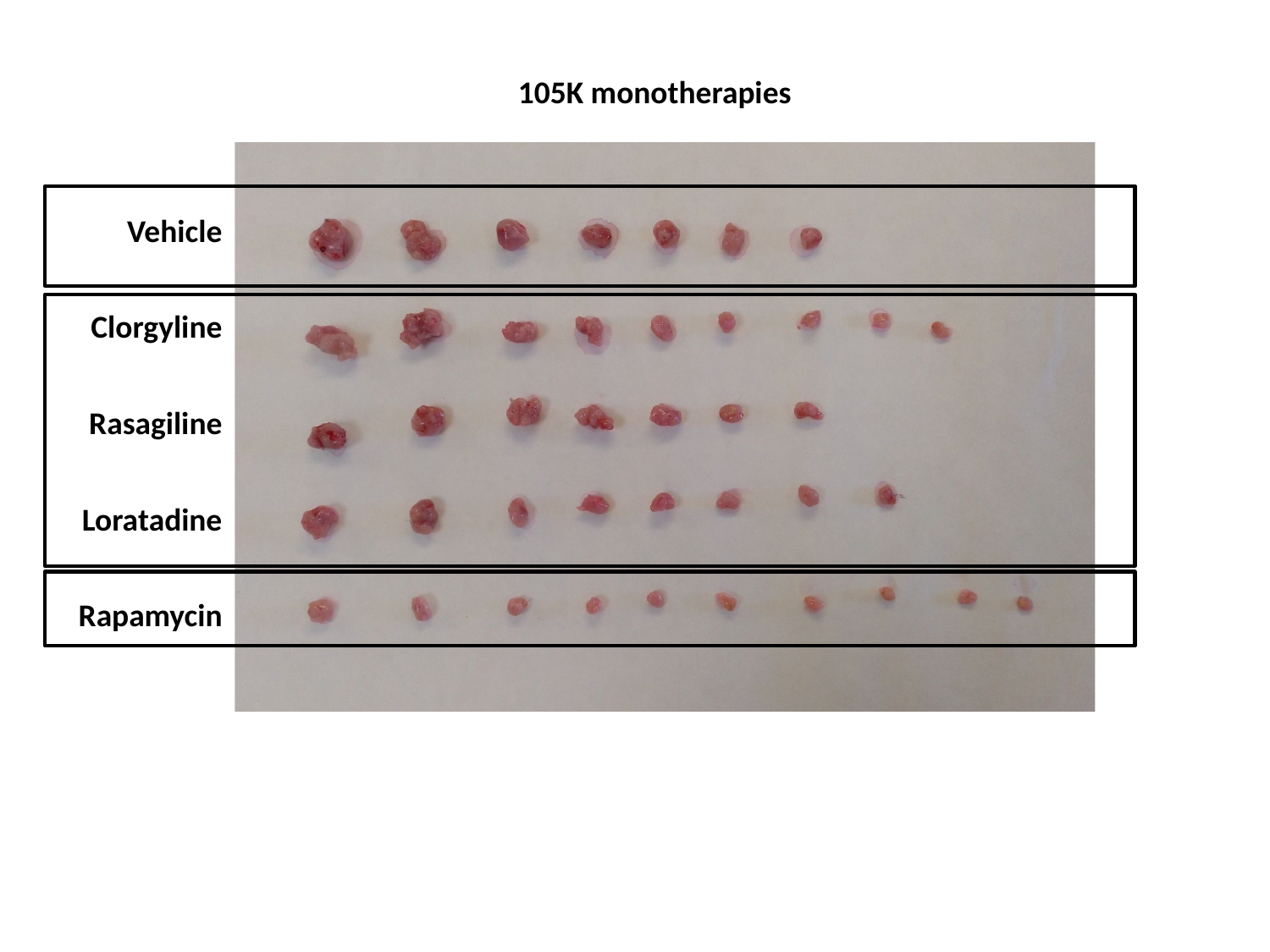

105K monotherapies
Vehicle
Clorgyline
Rasagiline
Loratadine
Rapamycin

## Slide 3
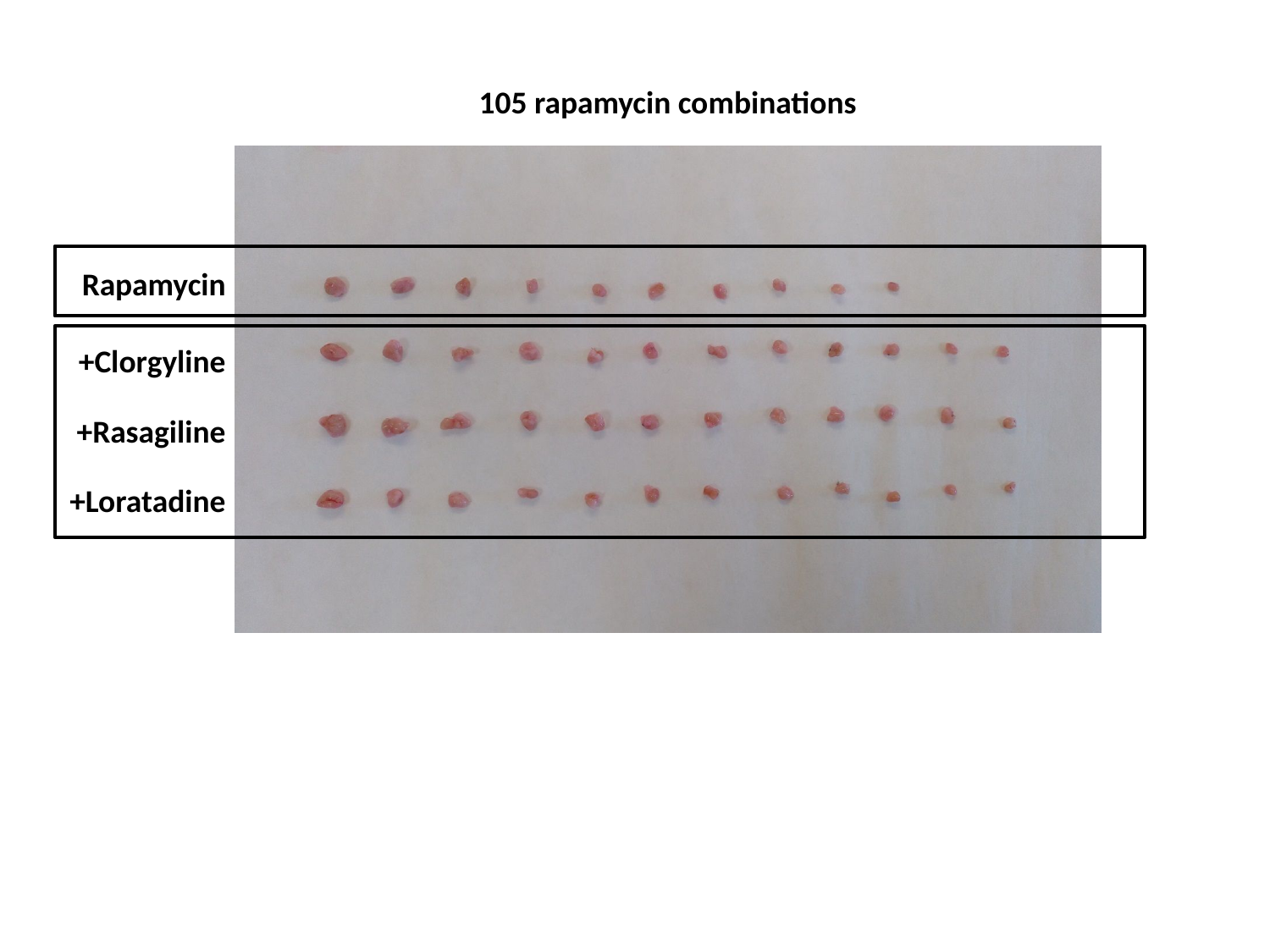

105 rapamycin combinations
Rapamycin
+Clorgyline
+Rasagiline
+Loratadine
