# Supplementary material for: Histamine signaling and metabolism identify potential biomarkers and therapies for lymphangioleiomyomatosis
Source: EMBO Mol Med. 2021 Aug 11;13(9):e13929. doi: 10.15252/emmm.202113929 (PMC8422079; doi:10.15252/emmm.202113929)
Supplement: Supplementary file 3 — Source Data for Expanded View and Appendix [file EMMM-13-e13929-s001.zip › EMM-2021-13929-V3-Figure_Source_Data_S1-S10-sd.zip!/EMM-2021-13929_Source_Data_SupplFigs_1-10/EMM-2021-13929_FigS4/EMM-2021-13929_FigS4A.docx]

````
## [1] "******[205579_at/HRH1]******"
## Call:
## coxph(formula = survModel ~ probeData)
##
##   n= 77, number of events= 13
##    (17 observations deleted due to missingness)
##
##             coef exp(coef) se(coef)    z Pr(>|z|)
## probeData 1.7324    5.6544   0.5499 3.15  0.00163 **
## ---
## Signif. codes:  0 '***' 0.001 '**' 0.01 '*' 0.05 '.' 0.1 ' ' 1
##
##           exp(coef) exp(-coef) lower .95 upper .95
## probeData     5.654     0.1769     1.924     16.61
##
## Concordance= 0.799  (se = 0.052 )
## Rsquare= 0.171   (max possible= 0.759 )
## Likelihood ratio test= 14.45  on 1 df,   p=1e-04
## Wald test            = 9.92  on 1 df,   p=0.002
## Score (logrank) test = 10.21  on 1 df,   p=0.001


---
title: "Replicate Masague survival checks"
author: Luis Palomero
date: "`r format(Sys.time(), '%d %B, %Y')`"
output:
  html_document:
    self_contained: no
    toc: true
    theme: united
params:
  affyExpr: '../data/20180316.nature.03799.minn/nature03799-s11.txt'
  survival: '../data/20180316.nature.03799.minn/nature03799-s12.txt'
  chipseqRef: '../data/20180301.archive/HG_U133_Plus_2.chip'
  genesToCompare: '../data/20180316.gene.list/gene.list.test.txt'
  outputFolder: '.'
---

```{r params, echo=FALSE}

if(exists('params')==FALSE){
  rm(list = ls())
  params = data.frame(
    affyExpr = '../data/20180316.nature.03799.minn/nature03799-s11.txt',
    survival = '../data/20180316.nature.03799.minn/nature03799-s12.txt',
    chipseqRef = '../data/20180301.archive/HG_U133_Plus_2.chip',
    genesToCompare = '../data/20180316.gene.list/20190109.set.hrh.txt',
    outputFolder = '.',
    stringsAsFactors = FALSE
  )

}

```

## Introduction

## Data preprocessing

```{r deps}

{
  library(circlize)
  library(ggplot2)
  library(ggrepel)
  library(ComplexHeatmap)

  library(limma)
  suppressMessages(library(knitr))
  suppressMessages(library(dplyr))
  suppressMessages(library(foreach))
  library(gtools)

  suppressWarnings(suppressMessages(library(survival)))

  opts_chunk$set(dev=c("png","postscript"))

  source('../src/r/tertilize.R')
}

```

### Loading Data

```{r data_load}

{
  affy = read.table(
    params$affyExpr,
    head = TRUE,
    row.names = 1
  )

  survival = read.table(
    params$survival,
    sep = '\t',
    header = TRUE
  )

  rownames(survival) = sub('-', '.', survival$Sample.Name)

  chipseq = read.table(
    params$chipseqRef,
    header = TRUE,
    sep ='\t',
    quote = "",
    stringsAsFactors = FALSE
  )

  genesToCompare = readLines(params$genesToCompare)
}

```

## Log2 scale and normalize affymetrix data

```{r log2_scale_affy}

affyNorm = affy %>% log(2) %>% normalizeQuantiles()

```

## Describe chipseq data

```{r chipseq_desc}

kable(
  head(chipseq),
  caption = "Chipseq refernce summary",
  format = 'pandoc'
)

```

## Preprocess data

###Find selected genes probe ids

```{r find_selected_genes_probe_ids}

chipseqFilt = chipseq %>%
  filter(GeneSymbol %in% genesToCompare) %>%
  arrange(GeneSymbol)

probes = chipseqFilt %>% .[['ProbeSetID']]

```

### Build complete dataset matrix

```{r build_ds_matrix}

{
  affyFilt = affyNorm[rownames(affyNorm) %in% chipseqFilt$ProbeSetID,] %>%
    t()
  probes = probes[probes %in% colnames(affyFilt)]


  survFilt = survival %>%
    select(
      Sample.Name,
      LMFS, LM.Event,
      BMFS, BM.Event
    )

  allData = merge(
    survFilt,
    affyFilt,
    by = 0

  )
  row.names(allData) = allData$Row.names
  allData = allData %>% select(-Row.names)
}

```

## Perform survival analysis

### Pipeline code

```{r perform_survivals}

buildSurvivalModel <- function(allData, modelName ){
  if(modelName == 'LMFS'){
    return(Surv(allData$LMFS, allData$LM.Event))
  } else { # 'BM'
    return(Surv(allData$BMFS, allData$BM.Event))
  }
}

iterateSurvivalModels <- function(allData, modelName, chipseqFilt, probes){

  fdrAdjust <- function(results){
    results %>% mutate(
      fdr = p.adjust(p, 'fdr') ,
      fdr.stars = stars.pval(fdr)
    )
  }


  colors<-c("forestgreen","blue","red")

  survModel = buildSurvivalModel(allData, modelName)

  survivals = foreach(probe = probes, .combine ='rbind', .final = fdrAdjust) %do% {
    geneName = chipseqFilt %>% filter(ProbeSetID == probe ) %>% .[['GeneSymbol']]

    print(sprintf('******[%s/%s]******', probe, geneName))
    probeData = allData[,probe]
    probeDataT = tertilize(probeData)
    geneName = chipseqFilt %>% filter(ProbeSetID == probe ) %>% .[['GeneSymbol']]

    theModel = coxph(survModel ~ probeData)
    sumModel = summary(theModel)
    print(summary(theModel))

    plot(
      survfit(survModel ~ probeDataT),
      col = colors,
      main = sprintf('%s(%s) | %s', probe, geneName, modelName),
      xlab = 'Time(yrs)',
      ylab = 'P(surv)'
    )
    legend(
      'bottomleft',
      legend = levels(probeDataT),
      col = colors,
      lty = 1,
      bty = 'n'
    )
    return(
      data.frame(
        disease = modelName,
        probeId = probe,
        gene = geneName,
        hr = exp(theModel$coefficients[1]),
        p = sumModel$coefficients[1,'Pr(>|z|)'],
        p.stars = stars.pval(sumModel$coefficients[1,'Pr(>|z|)'])

      )
    )
  }
  return(survivals)

}


```


### survivals for LMFS

```{r survivals_lmfs}

disease = 'LMFS'
survivalsSummaryL = iterateSurvivalModels(allData, disease, chipseqFilt, probes)
kable(
  survivalsSummaryL,
  caption = sprintf('HR summary at %s ', disease),
  format ='pandoc'
)

write.table(
  survivalsSummaryL,
  sep ='\t',
  quote = FALSE,
  row.names = FALSE,
  file = sprintf('%s/%s.hr.summary.tsv', params$outputFolder, disease)
)
```


### survivals for BMFS

```{r survivals_bmfs}

disease = 'BMFS'
survivalsSummaryM = iterateSurvivalModels(allData, disease, chipseqFilt, probes)
kable(
  survivalsSummaryM,
  caption = sprintf('HR summary at %s ', disease),
  format ='pandoc'
)

write.table(
  survivalsSummaryM,
  sep ='\t',
  quote = FALSE,
  row.names = FALSE,
  file = sprintf('%s/%s.hr.summary.tsv', params$outputFolder, disease)
)
```

### Display survivals results

```{r display_survivals}

survivalsSummary = merge(
  survivalsSummaryL %>% select(-disease),
  survivalsSummaryM %>% select(-disease),
  by = c('gene', 'probeId'),
  suffix = c('.LMFS','.BMFS')
)
kable(
  survivalsSummary,
  caption = sprintf('HR summary ', disease),
  format ='pandoc'
)

cor.test(survivalsSummary$hr.LMFS, survivalsSummary$hr.BMFS, method = 'pearson')


write.table(
  survivalsSummary,
  sep ='\t',
  quote = FALSE,
  row.names = FALSE,
  file = sprintf('%s/%s.hr.summary.tsv', params$outputFolder, 'both')
)

survivalsSummary$nSignif = as.numeric(survivalsSummary$p.LMFS < 0.05) +
  as.numeric(survivalsSummary$p.BMFS < 0.05)


ggplot(
  survivalsSummary,
  aes(
    x = hr.LMFS,
    y = hr.BMFS,
    fill = gene,
    label = sprintf('%s\n%s', gene, probeId),
    shape = as.factor(nSignif)
  )
) + geom_hline(
  yintercept = 1
) + geom_vline(
  xintercept = 1
) + geom_point(
  position = 'identity',
  aes(colour = gene)
) + scale_shape_manual(
  values = c('0' = 1, '1' = 16),
#  label =  c('N.S', '1 pval < 0.05'),
  name = 'pvals < 0.05'
) + geom_text_repel(
) + labs(
  title = sprintf('%s|%s (%s)', 'LMFS', 'BMFS', basename(params$genesToCompare))
) + scale_x_log10(
  limits = c(0.3, 5.7)
) + scale_y_log10(
  limits = c(0.3, 5.7)
) + scale_color_brewer(
  palette = 'Dark2',
  type = 'qual'
) + theme_classic()

ggsave(sprintf('%s/%s.hr.summary.png', params$outputFolder, 'both'))
ggsave(sprintf('%s/%s.hr.summary.eps', params$outputFolder, 'both'))


survivalsMatrix = survivalsSummary %>%
  select(hr.LMFS, hr.BMFS) %>%
  as.matrix()

rownames(survivalsMatrix) = sprintf('%s(%s)', survivalsSummary$gene, survivalsSummary$probeId )

survivalsSummary$p.stars.BMFS = factor(
  survivalsSummary$p.stars.BMFS,
  levels = c(' ', '.', '*', '**', '***')
)

survivalsMatrix = t(survivalsMatrix)

ha = columnAnnotation(
  df = survivalsSummary %>% select(
    LMFS = p.stars.LMFS,
    BMFS = p.stars.BMFS,
    gene = gene
    ),
  col = list(
    'LMFS' = c(
      ' ' = 'gray',
      '.' = 'cadetblue1',
      '*' = 'cadetblue2',
      '**' = 'cadetblue3',
      '***' = 'cadetblue4'),
    'BMFS' = c(
      ' ' = 'gray',
      '.' = 'darkseagreen1',
      '*' = 'darkseagreen2',
      '**' = 'darkseagreen3',
      '***' = 'darkseagreen4'
    )
  )
)

Heatmap(
  survivalsMatrix  ,
  col = colorRamp2(c(0.5, 1, 2), c('blue', 'white', 'red')),
  name = 'Hr',
  top_annotation = ha,
  column_title = basename(params$genesToCompare)
)

```
````
